# Bridging the Gap between Field Experiments and Machine Learning: The EC H2020 B-GOOD Project as a Case Study towards Automated Predictive Health Monitoring of Honey Bee Colonies

**DOI:** 10.3390/insects15010076

**Published:** 2024-01-22

**Authors:** Coby van Dooremalen, Zeynep N. Ulgezen, Raffaele Dall’Olio, Ugoline Godeau, Xiaodong Duan, José Paulo Sousa, Marc O. Schäfer, Alexis Beaurepaire, Pim van Gennip, Marten Schoonman, Claude Flener, Severine Matthijs, David Claeys Boúúaert, Wim Verbeke, Dana Freshley, Dirk-Jan Valkenburg, Trudy van den Bosch, Famke Schaafsma, Jeroen Peters, Mang Xu, Yves Le Conte, Cedric Alaux, Anne Dalmon, Robert J. Paxton, Anja Tehel, Tabea Streicher, Daniel S. Dezmirean, Alexandru I. Giurgiu, Christopher J. Topping, James Henty Williams, Nuno Capela, Sara Lopes, Fátima Alves, Joana Alves, João Bica, Sandra Simões, António Alves da Silva, Sílvia Castro, João Loureiro, Eva Horčičková, Martin Bencsik, Adam McVeigh, Tarun Kumar, Arrigo Moro, April van Delden, Elżbieta Ziółkowska, Michał Filipiak, Łukasz Mikołajczyk, Kirsten Leufgen, Lina De Smet, Dirk C. de Graaf

**Affiliations:** 1 Wageningen University & Research, 6708 PB Wageningen, The Netherlands; 2 BeeSources di Raffaele Dall’Olio, 40132 Bologna, Italy; 3 Institut National de la Recherche pour l’Agriculture, l’Alimentation et l’Environnement, 84914 Avignon, France; 4 Aarhus Universitet, 8000 Aarhus, Denmark; 5 Centre for Functional Ecology, Department of Life Sciences, TERRA Associated Laboratory, University of Coimbra, 3000-456 Coimbra, Portugal; 6 Friedrich-Loeffler-Institut, Bundesforschunginstitut für Tiergesundheit, 17493 Greifswald-Insel Riems, Germany; 7 Institute of Bee Health, University of Bern, 3012 Bern, Switzerland; 8 Stichting BEEP, 3972 LK Driebergen-Rijsenburg, The Netherlands; 9 Suomen Mehiläishoitajain Liitto, 00130 Helsinki, Finland; 10 Sciensano, 1180 Brussels, Belgium; 11 Ghent University, 9000 Ghent, Belgium; 12 Martin-Luther-Universitaet Halle-Wittenberg, 06120 Halle, Germany; 13 Universitatea de Stiinte Agricole si Medicina Veterinara Cluj Napoca, 400372 Cluj Napoca, Romania; 14 The Nottingham Trent University, Nottingham NG11 8NS, UK; 15 Uniwersytet Jagiellonski, 30-387 Krakow, Poland; 16 SCIPROM sàrl, 1025 Saint-Sulpice, Switzerland

**Keywords:** honey bee automated health monitoring, data collection method, data standardization and harmonization, work plans and protocols, stakeholder involvement in research, big data on honey bee colonies, bee data portal, beekeeping

## Abstract

**Simple Summary:**

Honey bees are very important for nature and food production. However, beekeepers’ work is continuously challenged by pests, pathogens, pesticides, and other impacts of the environment on their honey bee colonies, and, therefore, they would greatly benefit from up-to-date insights on the health condition of their bees. To disturb those bee colonies as little as possible, it is preferable that this information be collected in an automated way. In this article, we present the B-GOOD project as a case study to monitor the health of honey bee colonies in an automated, standardized way. The use of a similar approach by researchers in their future studies would allow the combination of different datasets on bee health. More data combinations would facilitate the use of machine learning to better and more accurately determine the thresholds for beekeeper interventions, the underlying mechanisms of honey bee colony health, and the prediction of health and colony losses, among other indicators.

**Abstract:**

Honey bee colonies have great societal and economic importance. The main challenge that beekeepers face is keeping bee colonies healthy under ever-changing environmental conditions. In the past two decades, beekeepers that manage colonies of Western honey bees (*Apis mellifera*) have become increasingly concerned by the presence of parasites and pathogens affecting the bees, the reduction in pollen and nectar availability, and the colonies’ exposure to pesticides, among others. Hence, beekeepers need to know the health condition of their colonies and how to keep them alive and thriving, which creates a need for a new holistic data collection method to harmonize the flow of information from various sources that can be linked at the colony level for different health determinants, such as bee colony, environmental, socioeconomic, and genetic statuses. For this purpose, we have developed and implemented the B-GOOD (Giving Beekeeping Guidance by computational-assisted Decision Making) project as a case study to categorize the colony’s health condition and find a Health Status Index (HSI). Using a 3-tier setup guided by work plans and standardized protocols, we have collected data from inside the colonies (amount of brood, disease load, honey harvest, etc.) and from their environment (floral resource availability). Most of the project’s data was automatically collected by the BEEP Base Sensor System. This continuous stream of data served as the basis to determine and validate an algorithm to calculate the HSI using machine learning. In this article, we share our insights on this holistic methodology and also highlight the importance of using a standardized data language to increase the compatibility between different current and future studies. We argue that the combined management of big data will be an essential building block in the development of targeted guidance for beekeepers and for the future of sustainable beekeeping.

## 1. Introduction

Honey bee colonies are at the center of great societal and economic concern because any problems related to their health and survival are experienced as a sign of the vulnerability of the environment and the service of crop pollination [1,2,3,4], the sensitivity of the beekeeping sector [5], and even potentially the susceptibility of human health [6]. In the past two decades, beekeepers that manage colonies of Western honey bees (*Apis mellifera*) have become increasingly troubled by colony losses caused by problems affecting the bees’ health, such as parasites, pathogens, reduction in floral resources, and exposure to pesticides. The constant crisis status caused by large and unsustainable honey bee colony losses has pushed beekeepers to continually monitor those losses throughout each season and over the winter, and also to identify potential risk factors that are generally analyzed in surveillance studies [7,8,9,10,11,12], or by experimental colony exposure [13,14,15,16]. Understanding the causes and mechanisms behind these colony losses is essential to preventing them and reversing this crisis. From a beekeeper’s perspective, nevertheless, they often consider the colonies healthy as long as the bees are entering and leaving the hives. For example, in a German monitoring program from 2004 to 2008, the majority of beekeepers (approximately 63%) reported no losses [8]. But “no losses” does not automatically translate into their colonies being healthy and performing optimally. Therefore, beekeepers are in dire need of information on what the real health condition of the colony is and how to keep the honey bees alive and healthy. But how can they check the colony’s health without disturbing the bees and creating further health decline due to harmful monitoring activities? And subsequently, how can they collect and interpret such information? The questions that arise for them are whether to act or not to act, when to do it, and how.

Currently, to estimate the health status of a honey bee colony, beekeepers need to open the hives manually. However, taking apart the hives for observation purposes means disturbing the colonies. Taking out the frames breaks the propolis envelope that is part of the bees’ immunity [17] and it increases the costs of thermoregulation, especially when the ambient temperature is low [18]. Furthermore, it may provoke robbing or defensive behavior in the bees. Also, queens may stop laying eggs or they may be accidentally killed by the beekeeper, which could be particularly problematic in autumn and winter when replacing injured or dead queens is difficult. To optimize colony resilience, and thus their potential healthy status, non- or low-disturbing monitoring methods should be prioritized [16,19,20]. Manual monitoring is also labor-intensive for beekeepers. To continue with it as a standard practice may result in beekeepers turning away from their hobby or business, as Potts et al. [5] showed in the beekeepers’ response to increased costs and labor related to the introduction of the Varroa destructor. Therefore, monitoring tools should be affordable and easy to apply for the beekeeper, as well as non-disruptive for the bees.

Consequently, we envision that the future of beekeeping will involve full-on implementation of technology as a holistic approach to better understand and improve the health of the honey bees and the sustainability of the beekeeping activity. Currently, the use of honey bee colony weighing scales is the most common of the new technologies for many professional beekeepers in the EU, so it is possible to foresee that in 20 years a large proportion of them will be using technology for day-to-day bee monitoring and computational guidance for their decision-making to keep those colonies healthy and high-performing (see Box 1). Automated systems allow monitoring from outside the hive, reducing disturbance for the colony, improving its resilience, and reducing labor for beekeepers. At the same time, automated monitoring enhances the harmonization and accuracy of the data collected. Large-scale harmonized data may result in a positive feedback loop that ensures reliable and optimized thresholds for tailor-made decision-making for beekeepers. The compilation of large datasets may also facilitate data mining to find general trends and solutions to honey bee services and colony losses related to stress, among others.

To make optimal use of new technologies, we need to understand how to interpret the raw data from a honey bee colony perspective [21,22,23,24]. Also, there may be markers, relations, or even predictors that are less obvious from a biological perspective, which could largely benefit from a machine learning or artificial intelligence approach [19,22,25]. As a result, there is a pressing need to collect frequently measured, large-scale, high-quality data, and also to test automated sensor technologies side by side with a more classical approach. If such high-quality data is combined with trained algorithms, it may be possible to evaluate the health status of a honey bee colony with accuracy, and to develop an early warning system to anticipate upcoming problems [22]. To that end, large datasets of high-quality data would greatly benefit from some form of labeling or annotation. Annotated datasets would allow supervised learning algorithms to be used for training, validating, and testing classification algorithms [22]. Unfortunately, the majority of the existing datasets are not sufficiently large, diverse, or continuous, and often lack annotations.

**Figure 1:**
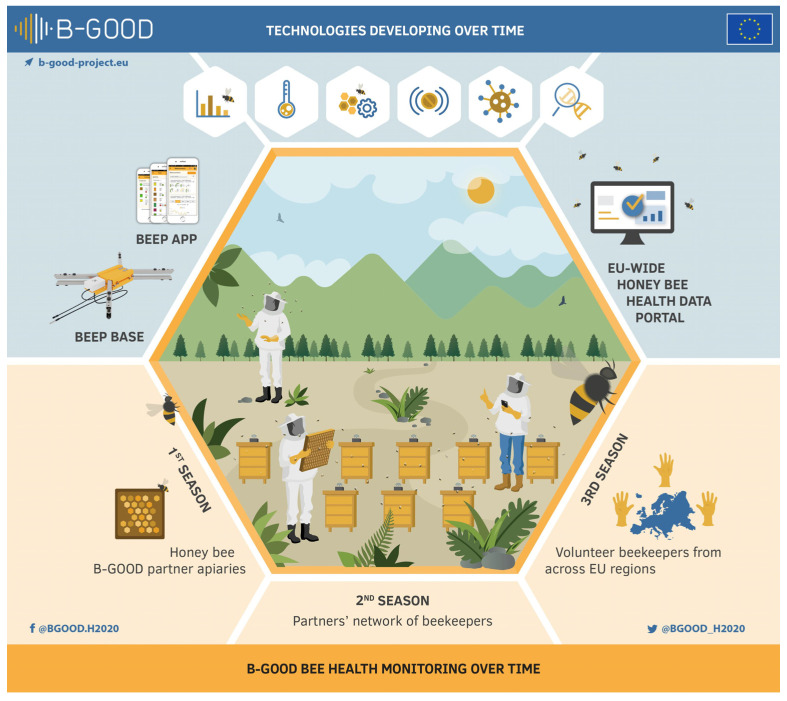
Infographic of the B-GOOD project. The project was setup in two steps: (1) development of new technology, and (2) setup of a main infrastructure for the collection of large-scale, frequently measured, high-quality data. The second step, a main infrastructure for large-scale data collection in a 3-tiered approach, was explained in more detail in this article and represented the backbone of bridging the gap between field work and machine learning.

An improved data model approach is needed to standardize and share information. A harmonized flow of data from various sources that can be linked at the colony level for different health determinants (colony, environmental, socioeconomic, and genetic condition) will ensure a new holistic way of data collection with an almost limitless sample size potential in time and space. Such an approach would maximize the usefulness of machine learning to gain insights into the complex underlying mechanisms of bee health, and to provide guidance for decision-making at local, regional, and international scales. In this article, we describe the B-GOOD project as a case study for such an improved approach, and to bridge the gap between field experiments and machine learning. The B-GOOD project was setup in two steps: (1) development of new technology, and (2) setup of a main infrastructure for the collection of frequently measured, large-scale, high-quality data (Figure 1).

Box 1 **B-GOOD’s envisioning of beekeeping.**

B-GOOD’s futuristic vision about the full-on implementation of technology as part of everyday beekeeping activities is speculative, although some technological advances like hive scales and monitoring devices have already generated interest and usage among beekeeping communities. However, since technoscience is still very much in its infancy, the question of how the rise of “bee tech” may benefit healthy beekeeping in the future remains open. To explore the anticipated role of digital technology in beekeeping, a B-GOOD workshop was organized with active project participants in June 2022. More than 60 people attended, including 25 B-GOOD beekeepers from the Netherlands, Belgium, Germany, and Switzerland. We explored the perceived challenges to beekeeping today and those associated with the health of honey bee colonies, and then we discussed potential solutions and how digital technology could help overcome those challenges in the future.

 

Dominating themes of discussion were the environmental factors and the multiple challenges beekeepers face to maintain the health and productivity of their colonies, such as strained floral resources, persistent diseases (Varroa mite), invasive species (Small hive beetle and Asian hornet), and intensive agriculture (green deserts), all viewed as adversely impacting the colony’s health. Often, beekeeping activities also disturbed the hives, involuntarily adding to those multiple environmental stressors and further impairing the health of the colony. There was consensus among participants that the new digital technologies (automated monitoring) could dramatically improve the beekeepers’ capacity to diagnose and respond earlier and quicker to those challenges by providing them with new methods, tools, indicators, and data. A variety of techno-scientific solutions were proposed during the workshop: automated image analysis (at the flight entrance, at the bottom board, or in the hive); bee and/or *V. destructor* counters; Lateral Flow Devices for pesticides and/or disease detection; predictive markers for colony health or condition change; threshold level detection with integrated pest management (IPM); automated alerts for guidance and actions; and the use of machine learning and artificial intelligence to analyze data of the hive, environment, resources, or pesticides in real time.

 

From this B-GOOD workshop, we gained the following insights:

The use of technology should lessen the reliance on manual inspection and monitoring, which are not only labor-intensive for beekeepers but also often detrimental to hive, and colony health.

The use of a standardized common language and an open-source digital platform will enhance data sharing and increase awareness of an average bee hive situation.

There is a need for a digital diagnostic tool set to help beekeepers ensure their bee colonies are healthy and beekeeping remains sustainable. By using an array of monitoring data and tools, it would be possible to better categorize the health of a bee colony and develop a Health Status Index.

Beekeepers could benefit from the data assessment and interpretation to make informed decisions and manage their hives accordingly. They could also better understand and confront some of the socioeconomic forces that threaten the health of their honey bees, for example, the prevalence and impact of pesticides.

 

From a B-GOOD perspective, we think that digital technology on its own will not solve the most urgent problems in the beekeeping sector and the honey bees’ environment. However, digital technology can be key in giving beekeepers, and other stakeholders closely connected to the beekeeping sector much-needed insights on the well-being of honey bees and their environment. And it could also enable them to better understand and interpret their experiences in specific situations, make pragmatic decisions, and take actions accordingly to ensure a sustainable future for their honey bees and new generations of beekeepers.

## 2. The B-GOOD Project as a Case Study for a Standard Health Monitoring Method of Honey Bee Colonies and the Development of a Health Status Index (HSI)

B-GOOD is an acronym that stands for Giving Beekeeping Guidance by cOmputatiOnal-assisted Decision making. It was a Horizon 2020 Framework Program (H2020) project funded by the European Commission and dedicated to healthy and sustainable beekeeping. The healthy beekeeping section mainly focused on finding a Health Status Index (HSI), a categorization of the health of a honey bee colony based on various indicators. Inspired by EFSA’s Healthy-B toolbox [26], we collected data from colony attributes (amount of brood and disease load), colony outputs (pollination service and honey harvest), and factors associated with external drivers (floral resource availability). Much of the data was collected automatically in a continuous stream, which formed the basis for determining and validating an algorithm to calculate the HSI. To ensure that the project ran smoothly, special attention was paid to quality assurance, information access, and the sharing of this big data. The main objective of developing a HSI in this project was to help guide beekeepers in their beekeeping management. If the HSI of a honey bee colony changes alarmingly, an alert could be sent to the beekeepers so that they could intervene on time. Other potential applications of the HSI were in the risk assessment of pesticide usage and in the impact of policy decisions on the welfare of honey bees. The sustainable beekeeping section combined the pursuit of a healthy bee colony, the safeguarding of the economic viability of the apicultural business, and the understanding of the ecological balance of the ecosystem.

A large data set was collected according to a 3-tiered structure in which our field of activity was gradually expanded. Tier 1 took place at the level of the partner institutions, i.e., eight B-GOOD partners who had the expertise and infrastructure to keep bees. At this level, the researchers themselves experienced any obstacles that arose and made the adjustments when necessary. Tier 1 was repeated every year alongside Tier 2 in the second bee season. In Tier 2, five B-GOOD partners guided eight neighboring beekeepers in their monitoring of up to three of their colonies. Tier 2 was also repeated the following bee season. Tier 3 took place with 58 beekeepers on a Pan-European scale. The data was collected in the following ways: (1) automated data flow through the BEEP Base Sensor System, (2) beekeeper observations from the hive logged through the BEEP app, and (3) lab analysis of samples collected several times a year regarding diseases, pesticides, and worker bees’ genotypes.

B-GOOD researchers were constantly looking to increase and diversify the data flow. Various innovative tools that were developed in an experimental setup found their way to the apiaries afterwards. Some of them were the measurement of vibrations, gas composition, and temperature in two dimensions in the beehive; a more advanced bee counter; and a molecular tool for honey bee genotyping. Work across the European Union (EU) was carried out on a dynamic landscape model (DLM), which captured where and when the most important flower resources (pollen and nectar) were available in each country. This model and data were then used to feed the EFSA’s ApisRAM model [27], a honey bee colony simulator, to predict changes in bee health conditions. B-GOOD researchers also assessed the socioeconomic factors of healthy and sustainable beekeeping, performed socioeconomic analysis using qualitative and quantitative research methods, and identified viable and sustainable business models for European beekeeping.

**Figure 2:**
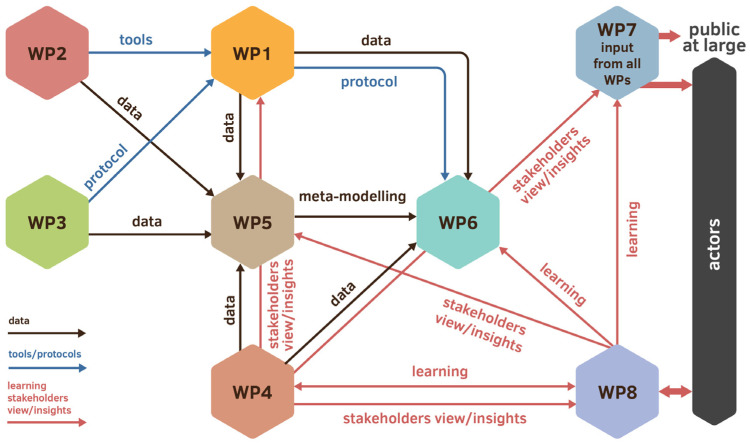
PERT chart of the B-GOOD project. This PERT chart shows the relationships between the different work packages (WPs) within the B-GOOD project based on the flow of data, protocols, and learning curves. The project consisted of six WPs dedicated to data collection and development of technology. WP1 provided the project with the main infrastructure for data collection; WP7 and WP8 focused on the communication and dissemination of information to the different actors. WP9 and WP10 were not included, as they involved project management and project ethics. See the main body of the text for more specific WP descriptions.

The B-GOOD project’s working structure consisted of 10 work packages (WPs). Work packages were sets of related tasks executed by different groups of collaborating project partners. The first six WPs were dedicated to the execution of the scientific program, and the four remaining provided administrative support (Figure 2). WP1 contributed to the operationalization of the HSI by collecting data from different health components of the bee colony under experimental and field conditions. To this end, WP1 developed detailed scenarios and protocols for end users to utilize and thus ensure the harmonization of the data and sample collection during and after the project. The collected data was then fed into WP5 and WP6, which were devoted to data analysis and decision-making, and to operationalization and application, respectively. Innovative tools were developed under WP2, which were eventually fed into WP1. WP3 was dedicated to ecological and environmental drivers, and was responsible for the collation of flower resources (nectar and pollen) data as the basis for developing a phenological model to integrate both (i) the dynamic landscape model developed in WP5 and used as input in the ApisRAM model to predict changes in bee health status, and (ii) the development of habitat suitability maps for beekeeping. WP4 focused on the socioeconomic factors of beekeeping. WP5 provided the data and analysis to establish the relationship between environmental, biological, and managerial drivers and bee health status. All these relationships were then incorporated into a holistic predictive simulation model of bee colonies in a large range of agricultural landscapes. In this scenario, various HSI components were validated to identify the most promising and relevant ones. WP6 operationalized the HSI in the digital bee data logbook and was responsible for the data streams, their storage, and sharing, as well as the integration of the decision-making support.

## 3. Infrastructure for Data Collection

The B-GOOD project facilitated and standardized a large-scale data collection of honey bee health indicators and gene pool characteristics according to EFSA’s Healthy-B toolbox [26]. All indicators selected had high relevance, high technical feasibility, and high priority. The data was obtained in different ways: classic, automated, semiautomated, and by lab analysis, and it covered different biogeographic regions of the EU.

**Figure 3:**
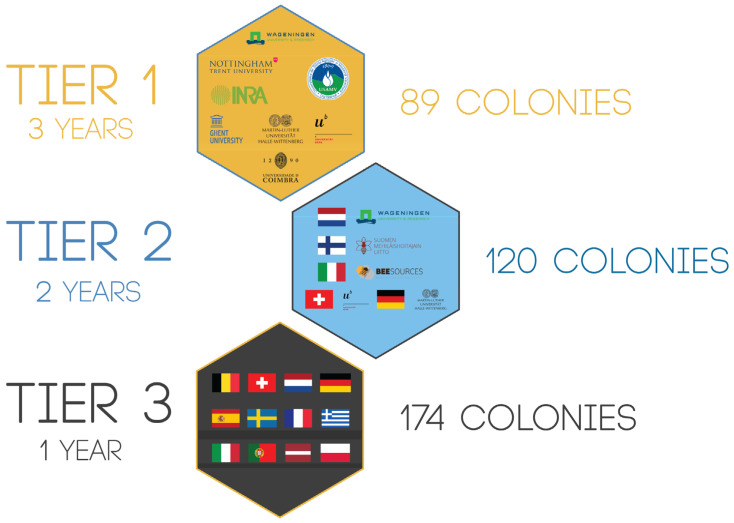
Data collection in a 3-tiered approach. The B-GOOD project collected data from variables inside and outside the hive related to the colony’s health status. The full data collection period spanned three bee seasons and ran through 12 countries. With the progression of the tiers, the EU’s geographical coverage increased, and the characteristics of beekeepers changed. In Tier 1, the research institutes performed the beekeeping; in Tier 2, specific beekeepers were selected and guided; and in Tier 3, beekeepers were randomly selected in the EU. In total, 106 beekeepers (including researchers from research institutes) and 383 colonies participated in the project.

The data collection occurred according to a 3-tiered process (Figure 3) that spanned three years (three bee seasons). The aim of this approach was threefold, and with each tier, we advanced toward the following goals:

1. More bee health (semi)automated monitoring in a bee and user-friendly fashion.
2. A ready to use end product of B-GOOD, validated by end users (primarily beekeepers) with a decreased amount of support.
3. A larger coverage of the EU territory with increased diversity of bees, hives, and business models, and a variety of environments (ecotypes) where the monitoring of the bee health took place.

In Tier 1 (2020–2022), eight apiaries were installed with eight honey bee colonies each. Because of their small size, they were called mini-apiaries, and each was kept by one of eight partner institutes across eight different countries (BE, NL, FR, DE, RO, PT, UK, and CH). In addition to these 64 healthy colonies, 25 honey bee colonies were added to NL and exposed to stressors in groups of five, one of which was the control group. For Tier 2 (2021–2022), 40 beekeepers were evenly selected over five countries (FI, NL, DE, CH, and IT), where each B-GOOD partner coordinated and guided the beekeeping activities. For Tier 3 (2022), an open call was placed within the B-GOOD Pan-European network (EU Bee Partnership, COLOSS Honey Bee Research Association, and national beekeepers’ associations), and from the more than 100 respondents, we selected 58 beekeepers from 12 countries. Each beekeeper in Tier 2 and Tier 3 was invited to participate with three honey bee colonies. With the progression of the tiers in time and space, more variables came into place. As the coverage level of testing shifted from a restricted local institutional EU country in Tier 1 to a North-South EU country axis in Tier 2 and to a Pan-European level in Tier 3, a large variation was represented in subspecies, ecotypes, gene pools, environmental conditions, beekeeping management practices, and business models. Whereas in Tier 2, experienced beekeepers that used predetermined business models were selected, in Tier 3, volunteers from across EU regions that used any business model were randomly selected. All these participating colonies within the apiaries conformed to the main body of research to operationalize the EFSA’s Healthy-B toolbox [26] and were used to validate if B-GOOD’s (semi)automated monitoring tools were technologically mature enough for use, described as a Technological Readiness Level (TRL) higher than 6 in de Graaf et al. [28].

Each B-GOOD apiary in Tiers 1, 2, and 3 started the experiment with local honey bee colonies that were assumed to be healthy. Healthy at the start of the project was defined as “not sick and performing within normal parameters related to their purpose, e.g., honey harvest” (see Box 2). The exception herein were the 25 additional colonies in Tier 1 that were exposed to stress in a similar way to what researchers described in van Dooremalen et al. [16]. The experimental stress exposure applied in Tier 1 in 2020 consisted of inducing the following changes: (i) reproductive status, (ii) natural parasite load, (iii) exposure to the neonicotinoid acetamiprid, and (iv) reduced pollen resource availability. In the second year of tier 1 (2021 and 2022), the pesticide-exposed group was replaced by a group of colonies that were V. destructor-tolerant, a Dutch local selection line called NSC, according to Claeys Boúúaert et al. [29].

Box 2 **B-GOOD’s healthy colonies (text adapted from de Graaf et al. [28]).**

Health is a very complex and anthropogenic concept, and nowadays it is thought of more as an “absence of disease.” For example, Huber et al. [30] described human health as a dynamic concept with 556 health indicators, categorized into six dimensions and 32 underlying aspects. In evolutionary biology, health may be more related to the survival and fitness of organisms, where fitness represents the quantitative reproductive success of a genotype or phenotype in a given environment. But fitness is also a very complex concept, especially in honey bee colonies, because, from the perspective of the evolution of eusociality [31] many individuals do not reproduce within the colony. Honey bee health, whether simply based on the absence or presence of disease, is the driver for survival or reproduction, it indicates the level of well-being, and it is influenced by the environment in which the colonies are located and by the beekeeper, who may choose or not to intervene. This complexity at any point in time and space often leads to a lack of a single cause for colony losses, and the subsequent conclusion is that many contributing stressors may act in concert [2].

We propose to designate the superorganism level, the colony, as the unit for health, and to distinguish between current and future health.

- Current health: No clinical symptoms of disease were detected by visual inspection and supported by laboratory analysis. In addition, when food resources are available, there should be brood in all stadia (BIAS) and foraging activity when weather permits it. When there are no food resources available, or foraging activity is hampered, there should be sufficient storage of resources for survival until this down period ends.
- Future health: Able to survive the winter or other long period of low resource availability, and to reproduce or to be willing to reproduce during the growing season.

Moreover, the perception of honey bee colony health may differ between different actors because the health status can be linked to beekeeping business models (social stratification); gene pool based on geographic location or local vs. imported bees (ethnicity); and season or resource availability (situational factors). From a health perspective, B-GOOD advocates that locally adapted honey bees will have an increased chance of survival compared to bees from elsewhere [32,33,34], and will have better intrinsic health.

The main technology implemented throughout the infrastructure of the B-GOOD project was a multi-sensor system installed in all 383 colonies under investigation. The BEEP Base Sensor System (https://beep.nl/index.php/measurement-system-2 (accessed on 19 January 2024)) consisted of 89 systems in Tier 1, 120 systems in Tier 2, and 174 systems in Tier 3. BEEP included sensors for weighing the hive, measuring the temperature near the brood, and recording the sound below the brood. A BEEP app (https://beep.nl/index.php/beep-app (accessed on 19 January 2024)) was used as a digital logbook to record manual inspections and the automatically acquired data from the sensors. A long-range, low-power (LoRa) gateway was installed for the wireless remote data transmission. Only in Tier 1 has a local weather system been installed. For validation purposes and to develop in WP2, some accelerometers and knock devices were installed in three mini-apiaries.

Not all measurements were fully performed at the local apiaries. Part of them required laboratory disease and gene pool analysis, for which logistics was necessary to ship some samples from the B-GOOD apiaries to the B-GOOD laboratory partners. The beekeepers were guided to do the sampling. To avoid transporting many parcels across EU borders, samples of live bees were shipped within a country to a central collection point (a local B-GOOD partner). At the national collection points, the bees were frozen and sent in batches on dry ice to the B-GOOD laboratories. In general, only material transfer agreements (MTAs) were needed as proper shipping documentation according to EU regulations. When necessary, some samples, derived materials, or products were shipped for further processing and analysis between different laboratories.

B-GOOD’s infrastructure was used to collect data to perform bee health assessments and validations at colonies and apiary levels, including different beekeeping business models. At the same time, this infrastructure was used to disseminate knowledge from researchers to beekeepers using a learning-by-doing approach. Learning by doing has been known to be highly effective [35], with end users, the beekeepers, being the best people to validate the methods. Direct communication between partners occurred through a digital platform (Microsoft Teams), with meetings on a monthly basis to discuss the project progression, tackle user problems, and gain feedback. All partners were continuously involved in updating the work plans and giving feedback about the processes and the content. The communication between the coordinating and guiding B-GOOD partners and the B-GOOD beekeepers was done in a tailor-made fashion for each country and local setting via email, WhatsApp, Slack, or Microsoft Teams.

## 4. Data Collected

The B-GOOD project collected raw data from 2020 to 2022. The data collection spanned three field seasons in three tiers: Tier 1 (2020–2022) with B-GOOD consortium members, Tier 2 (2021–2022) with selected and guided beekeepers, and Tier 3 (2022) with randomly selected beekeepers. Table 1 shows for each tier all the variables that we measured and logged for each B-GOOD colony. These variables involved data retrieved from sensors, annotations, experimental observations and inspections, laboratory analysis, management actions, and weather stations, and they also showed the frequency in which they were measured. Regarding management actions, due to the large number of options available, we only showed actions registered over 2020 and 2021. The raw data was checked, double-checked, and processed when necessary, and it was uploaded to the B-GOOD Bee Health Data Portal (https://beehealthdata.org/login (accessed on 19 January 2024)). The portal was used to store and share raw and preprocessed data sets for further analysis. Access to the data sets was setup per B-GOOD partner organization and was guided by a project data access policy. By the end of the project, all data sets were published and shared publicly.

To operationalize the HSI, researchers in Tier 1 collected data on indicators with high scores on relevance, technical feasibility, and priority, tabulated as H-HH in the Healthy-B Toolbox according to EFSA [26]. Novel health monitoring tools were added as they emerged from the technology development part of the B-GOOD project, and when they proved successful and their TRL was higher than 6. In order to move from classical labor-intensive and bee-invasive manual data collection to automated measurements, and to better interpret the data, both classical [36,37] and automated measurements were performed simultaneously. With the progression of the tiers, and based on feedback sessions and surveys, the classical measurements were gradually reduced in size or frequency. The data collection in Tier 1 was complex and labor-intensive, and hence executed by researchers. Due to its extensiveness, we expected that Tier 1 would most likely lead to new insights on essential indicators for bee health assessments at the end of the B-GOOD project. Other insights gained during Tier 1 were related to which monitoring tools and protocols were the most bee and user-friendly. Those tools and protocols were included in Tier 2 and, after a subsequent selection process, were also included in Tier 3. Beekeepers, as the main end users of the B-GOOD project, were responsible, with the guidance of B-GOOD partners, for implementing the data collection in Tier 2 and Tier 3, and thus the use of bee and user-friendly novel protocols and tools was particularly important in every colony. These beekeepers helped to validate the B-GOOD monitoring approach by testing the tools, giving feedback, and collecting manual data that was added to the automated data. The analysis of such integrated data would enable the subsequent development of guidance for their beekeeping activities.

The operationalization of the HSI was defined as the process of moving forward between tiers, increasing the coverage of European territory and end users’ B-GOOD activities. In each tier, the process was guided by periodic feedback sessions and informal winter surveys with the participants. The collected information was used to update the tier workflow, work plans, and protocols, and to prepare the workflow for the subsequent tier. After analyzing the information, some protocols were discarded for the next tiers, especially when those protocols were considered to be too laborious for beekeepers and too invasive for the bee colonies (for example, the Liebefeld measurements to estimate the number of bees, brood, and honey inside the colonies; see Table 1).

## 5. Harmonization and Standardization of the Workflow

To obtain high-quality data, monitoring was done in a harmonized and standardized way. The B-GOOD project used work plans, scientific protocols, and manuals that were adapted and optimized for the project’s own purpose, and included user support manuals for the BEEP system and the BEEP app. Field observations and sampling for lab analysis of diseases were performed synchronously according to detailed procedures in all apiaries. Technical support was also offered through a BEEP helpdesk.

Each tier had a work plan, a set of protocols, and digital manuals of the applicable technology and software (for all workplans and protocols, see Supplementary Information S1). In alignment with the setup, with the progression of the tiers, the following happened:

1. The guidance and standardization of beekeeping decreased.
2. The number of protocols and invasiveness for the honey bee colonies decreased.
3. The readability and user-friendliness of the protocols increased.
4. The reliance on automated sensors and digital logging of management actions increased.

**Figure 4:**
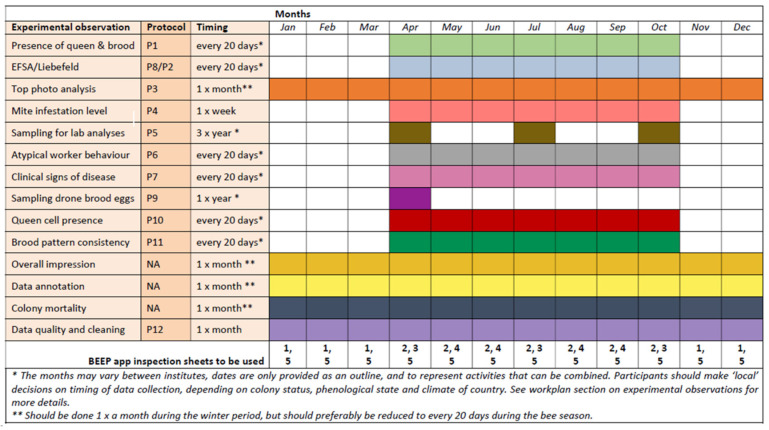
Screenshot of the harmonization table of Tier 1 work plan. It shows the timing of measurement for the different observations guided by the different protocols and the respective inspection checklists. Coloured cells show in which months experimental observations are expected to be performed. Five inspection sheets were prepared for use in the BEEP app, covering the different protocols: 1 Winter; 2 Varroa, 3 Summer+, 4 Summer, 5 Health. Wageningen Research, as the coordinating partner, prepared the inspection sheets in the BEEP app for the B-GOOD participants. (See Supplementary Information S1 for the full workplan of Tier 1, and Supplementary Information S2 for an overview of the inspection checklists used).

We based the work plans and protocols on the previous experience and expertise of B-GOOD partners [16,38,39]. The classical measurements were mostly based on articles from the COLOSS BEEBOOK [36,37,40,41] and the OIE guidelines [42]. Table 2 shows an overview of the protocols that were used in the different tiers to standardize the data collection. Figure 4 shows an example of a table (work plan for Tier 1) that facilitated the data harmonization. The work plans, protocols, and manuals were also uploaded to the online BEEP app (https://app.beep.nl (accessed on 19 January 2024)), where supporting inspection checklists were made freely available (see Supplementary Information S2). Inspection checklists were premade forms that helped the users report standard measurements and/or observations (see also the section “Standardization and implementation of data language”). The work plans and protocols of all tiers were made openly accessible via the B-GOOD Bee Health Data Portal (https://beehealthdata.org/ (accessed on 19 January 2024)) at the end of the project. Early versions of the protocols were prepared during the first two years of the project, and were published as practical abstracts on the EIP-AGRI platform in 2021 at https://ec.europa.eu/eip/agriculture/en/find-connect/projects/giving-beekeeping-guidance-computational-assisted (accessed on 19 January 2024).

A separation was made between the protocols for beekeepers and those for laboratories. Professional laboratory protocols to determine disease load in a sample taken by a beekeeper also needed to be standardized, harmonized, and optimized. Outcomes needed to be repeatable and of high quality, which could only be obtained by accredited laboratories. The pathogens and parasites of honey bees that were investigated through laboratory analysis were chosen according to EFSA’s Healthy-B toolbox [26] and recent scientific publications [43,44]. Standard laboratory protocols for the analysis of the level of infestation or infection with *Varroa destructor*, *Nosema apis*, *Nosema ceranae*, *Paenibacillus larvae*, *Melissococcus plutonius*, and *Malpighamoeba mellificae*, and the honey bee viruses acute bee paralysis virus (ABPV), black queen cell virus (BQCV), chronic bee paralysis virus (CBPV), deformed wing virus (DWV; genotypes A and B), and sacbrood virus (SBV) were established by two B-GOOD partners, the National Reference Laboratories of Belgium and Germany, according to standard procedures described by the European Reference Laboratory (EURL) for bee health. In summary, the level of infestation with the parasitic mite *V. destructor* was determined by washing a minimum of 100 bees in 100% ethanol, and separating the mites from the bees with a sieve of mesh size 3–4 mm [42]. For the pathogens *N. apis*, *N. ceranae*, *P. larvae* (causative agent of American foulbrood, AFB), *M. plutonius* (causative agent of European foulbrood, EFB), and the honey bee viruses ABPV, BQCV, CBPV, DWV A, DWV B, and SBV, DNA and RNA were extracted from a homogenate of 15 bees per sample (10 bees in 2020). These nucleotide extracts were used in real-time PCR for *N. apis*, *N. ceranae*, *P. larvae*, and *M. plutonius* and in Reverse Transcription real-time PCR for honey bee viruses [45,46,47,48,49,50], 2019; see Table 3 for the primers and probes used for the analysis). Additionally, the samples in which *P. larvae* DNA was detected were analyzed with bacteriological methods. Samples in which *Nosema* sp. DNA was detected were studied under the microscope to count the number of spores. With the first 25 samples, a laboratory proficiency test was performed between the two National Reference Laboratories. We decided that the laboratory that had the highest sensitivity for a certain analysis would perform this analysis on all samples in the project. All honey bee samples were taken alive in the field and were immediately frozen with dry ice or put in a −80 °C ultra-freezer. The samples remained in an uninterrupted cold chain until they were analyzed. From the country of origin, the samples were sent to the closest of both laboratories, where one part of the analysis was performed. After this laboratory finished the analysis, the subsample of homogenized bees was forwarded to the other laboratory for the second part of the analysis.

## 6. Standardization and Implementation of Data Language

The collection of high-quality data is a prerequisite for accurate data analysis, especially for scientific purposes, and requires high-quality collection methods. In the B-GOOD project, both scientists and beekeepers cooperated and collected data from their honey bee colonies using work plans and protocols. For such a collaboration between them to work, structured and standardized data collection was essential. The use of a standard and self-explanatory language was also necessary to optimize a high-quality collection method. The use of a consistent language was, and potentially will be, especially useful when implemented across all communication platforms, work plans, protocols, digital logbooks, and ultimately in the scientific reports and articles associated with this and other honey bee studies. For this purpose, we have used a list of categories as the basis for the standardization of the data language. The standardized list was developed by the BEEP Foundation, a B-GOOD partner that brought background knowledge to the project. The category list was further fine-tuned in the B-GOOD project with the help of the B-GOOD consortium, and it would remain open for additional input in the future. The use of a standardized list had the following advantages:

1. It enabled researchers to select and collect the data they needed for analysis.
2. It helped data collectors enter high-quality data by using standard options.
3. It facilitated structured data storage, enabling data comparison between research participants, colonies, locations, and potential meta-analysis research studies.

**Figure 5:**
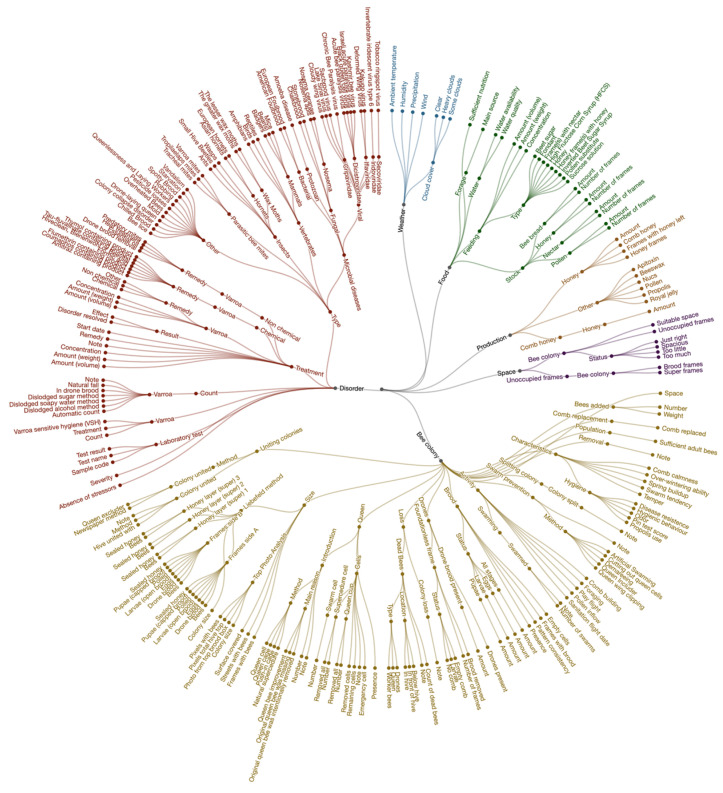
Graphical overview of some categories and their hierarchical relationships. The graphic shows a subset of the full list, namely, the subcategories under the “Inspection” category. Note that not all 24 frames of the Liebefeld method for determining colony size and composition were depicted. A high-resolution figure is available at https://beep.nl/storage/beep_inspection_categories_2022_06_02.png (accessed on 19 January 2024).

The standardized list and the definition of the terms were compiled by the BEEP Foundation after consultation with approximately 30 sources that included beekeeping logbooks, apiary management systems, digital record-keeping tools, the COLOSS BEEBOOK [36,37,41], the Healthy-B toolbox [26], FAO’s honey bee diseases guide [53], and various websites (Apiservices, Beebreed, Imkerpedia, and Wikipedia). The list was put in a hierarchical structure, as opposed to an alphabetical structure. A hierarchical order allowed the user to keep an overview of various key subjects and understand how the categories related to each other. This structure provided context for each category term and eased its practical application. The hierarchy could also be used to deepen the level of detail of the data collection; the further down you went in the hierarchy, the more detailed the type of data the term resulted in. Figure 5 shows a subset of the list in a diagram with a total of 395 terms in the category “Inspection” that included beekeeping concepts as well as general terms. The figure excluded all 24 frames from the “Liebefeld method”, which would increase the total subcategories to 368, leading to a total of 763 “Inspection” categories. The complete standardized list plus definitions and references were made available at https://beep.nl/beep-app/data-categories-2 (accessed on 19 January 2024) under a public license for reuse.

The standard list was implemented in the B-GOOD project for field data collection via the BEEP app in twelve languages. The BEEP app and the list were updated, expanded, and/or translated whenever needed to facilitate specific B-GOOD requirements or features. The following are the twelve main categories of more than 400 categories:

- Apiary
  ▪ Hive
    o Queen
      - Inspection
      - Bee colony
      - Disorder
      - Food
      - Overall
      - Production
      - Weather
    o Device
      - Measurement

In the BEEP app, each category of the standardized list had specific properties to enable high-quality data collection. These properties included the following:

- Category name (in English and translated)
- Parent (the identifier of the parent category)
- Definition (the meaning of the category name in English plus the source)
- Input type (the kind of input field, such as an option list, smiley, or number)
- Physical quantity (the unit)
- Long or short description (long meaning a data collection instruction with optional visuals, such as a top photo analysis [see protocol Tier 1, P3 in Supplementary Information S1]; short meaning a sentence).

Each category was, in practice, a data entry field in the BEEP app, a column in a table in the BEEP database where the app stored raw data, and various columns in data exports from the app. This organization allowed the scientists managing the data collection in the B-GOOD project to have a structured approach. There were 40 types of input fields that allowed the collection of quantitative and qualitative data. Some of them included Boolean options (to select yes or no), smileys (three types; green, red, or yellow), options lists (categories), labels (the name of an option list), numbers (natural, integers, or with decimals allowed), and scoring options (poor up to excellent).

From a front-end user perspective, the implementation of the standardized list required data collected both from personal inspections and automated sensors. The beekeeper could perform manual observations (e.g., in case of atypical behavior of the bees), the observations could be recorded automatically by the sensors (e.g., a change in hive weight), and a supporting algorithm could be used (e.g., when a threshold was crossed, an alert was sent out). In addition, the beekeeper could perform management actions manually (e.g., split or feed a colony), or an algorithm could potentially suggest specific actions in the future (e.g., to prevent swarming). In any of these cases, along with those observations and actions, it was of primary importance to also store metadata regarding the date, time, colony, hive type, apiary, location (address or GPS coordinates), and sensor device identification. If data is used for machine learning, additional data annotation could be required as well [22]. In general, this metadata collection would increase the data quality, but it would also provide context over the type of environment and beekeeper, and it would increase the opportunity for other researchers to use the full data set in other meta-analyses.

## 7. Managing High-Quality Data in an Open Science Platform

The definition of custom inspection checklists, either privately or as part of a research project, supports various types of beekeeping practices, for example, Varroa mite treatment, colony characteristics determination for queen selection, or swarm control. The standardized list to collect honey bee data was at the core of the BEEP platform; however, this list could be used for research or other purposes as well. In general, but with the BEEP system as an example, the development, maintenance, and support of digital tools may advance bee health research, and help beekeepers make data-based decisions to respond quickly to the needs of their honey bee colonies.

B-GOOD’s data was continually stored in the BEEP platform database. Research participants could see and download all the data linked to their user accounts, as well as that of others, after joint collaboration. Researchers could also use a research feature developed within the B-GOOD project, which allowed them the following: (1) to set up specific research, e.g., split in three tiers, (2) to give their consent to share the data they have entered, (3) to see an overview of the collective data shared and stored over time, and (4) to download the data. There were two ways to download data: via file format (csv or xlsx) or via the Application Programming Interface (API; in json format).

From a software development perspective, the standardized category list was dynamic in two ways. First, the front-end users of the software (BEEP app) could select the subcategories in the “Inspection” category they wanted to enter data for. And second, when new category terms were needed, they could be requested and added. The data architect added new category terms after doing a vetting process and using the administration user interface. The vetting process included a revision of the uniqueness of the new term compared to the already existing categories, desk research about the generally used terms among beekeepers, and the addition of the definition of the new category. After a category was used once, it could not be removed.

## 8. Conclusions

We have presented the B-GOOD working method as a case study on how to harmonize and standardize data collection to monitor the health of honey bee colonies across Europe. This method would potentially allow us to link field measurements with future predictive simulation models and machine learning. The infrastructure of the B-GOOD project allowed the continuous collection of frequently measured, diverse, large-scale, high-quality data with a multifaceted approach that encompassed many different biogeographical regions. We regarded this method as a step towards incorporating and generating datasets that could allow the monitoring of the current and future health status of honey bee colonies, and give beekeepers guidance in their decision-making processes.

In the B-GOOD project, we have highlighted the importance of using a holistic approach in which the honey bees’ colony parameters, the socioeconomic factors of the beekeeping sector, the genetics, the ecology, and the environment were integrated to capture the complex structure of honey bees’ health. The determination of a common honey bee health status index (HSI), based on the Healthy-B toolbox, would help risk assessors, authorities, and the plant protection and veterinary medicine industries assess the honey bees’ health condition in real time and across geographical locations, and measure the effect of beekeeping management decisions and actions. We argue that this type of standardized and harmonized methodology would be an essential building block for long-term research on honey bee colony health and for the development of targeted guidance for healthier and more sustainable beekeeping in the future. We expect that by sharing our methods, we will allow other researchers to build upon our efforts and thus expand the amount of honey bee data available for machine learning and predictive modeling.

## Figures and Tables

**Table 1:** Variables and frequency of measurements logged in B-GOOD colonies. The frequency of measurements has been split for Tier 1 (2020–2022), Tier 2 (2021–2022), and Tier 3 (2022). NA = not applicable.

Variable	Category	Data/Units	Tier 1	Tier 2	Tier 3
Weight	Automated data	kg	15 min	15 min	15 min
Ambient temperature	Automated data	°C (Celsius degrees)	15 min	15 min	15 min
In-hive temperature	Automated data	°C (Celsius degrees)	15 min	15 min	15 min
Sound	Automated data	Frequency count (122–583 Hz)	15 min	15 min	15 min
Battery	Automated data	Volt	15 min	15 min	15 min
Signal strength (data transmission)	Automated data	dBm	15 min	15 min	15 min
Signal noise (data transmission)	Automated data	dB	15 min	15 min	15 min
Sufficient adult bees	Data annotation	Yes/no	7–30 days	7–30 days	7–30 days
Brood in all stages	Data annotation	Yes/no	7–30 days	7–30 days	7–30 days
Presence of queen	Data annotation	Yes/no	7–30 days	7–30 days	7–30 days
Suitable space	Data annotation	Yes/no	7–30 days	7–30 days	7–30 days
Absence of stressors	Data annotation	Yes/no	7–30 days	7–30 days	7–30 days
Sufficient nutrition	Data annotation	Yes/no	7–30 days	7–30 days	7–30 days
General impression	Experimental observation	Good, average, bad (smileys)	7–30 days	7–30 days	7–30 days
Eggs	Experimental observation	Estimated number of cells	Every 21 days (beekeeping season)	NA	NA
Larvae	Experimental observation	Estimated number of cells	Every 21 days (beekeeping season)	NA	NA
Bees	Experimental observation	Estimated number of cells	Every 21 days (beekeeping season)	NA	NA
Pollen	Experimental observation	Estimated number of cells	Every 21 days (beekeeping season)	NA	NA
Sealed honey	Experimental observation	Estimated number of cells	Every 21 days (beekeeping season)	NA	NA
Pupae (capped brood)	Experimental observation	Estimated number of cells	Every 21 days (beekeeping season)	NA	NA
Drone brood	Experimental observation	Estimated number of cells	Every 21 days (beekeeping season)	NA	NA
Atypical behavior	Experimental observation	Yes/no	Every 21 days (beekeeping season)	NA	NA
Colony loss	Experimental observation	Yes/no	When necessary	When necessary	When necessary
Dead bees	Experimental observation	Yes/no	Every 21 days (beekeeping season)	Every 30 days (beekeeping season)	NA
Varroa natural fall	Experimental observation	mites/day	Once a week	optional	NA
Clinical signs of disease	Experimental observation	Categorized by type	Every 21 days (beekeeping season)	Every 30 days (beekeeping season)	NA
Presence of eggs	Experimental observation	Yes/no	Every 21 days (beekeeping season)	Every 30 days (beekeeping season)	Every 30 days (beekeeping season)
Presence of larvae	Experimental observation	Yes/no	Every 21 days (beekeeping season)	Every 30 days (beekeeping season)	Every 30 days (beekeeping season)
Presence of pupae	Experimental observation	Yes/no	Every 21 days (beekeeping season)	Every 30 days (beekeeping season)	Every 30 days (beekeeping season)
Queen presence	Experimental observation	Yes/no	Every 21 days (beekeeping season)	Every 30 days (beekeeping season)	Every 30 days (beekeeping season)
Top photo analysis	Experimental observation	Estimated number of bees	Every 30 days during winter, and every 21 days during beekeeping season	Every 30 days	NA
Queen cell presence	Experimental observation	Yes/no	Every 21 days (beekeeping season) *	Every 30 days (beekeeping season) **	NA
Queen cell type	Experimental observation	Supersedure, emergency, cup, swarm	Every 21 days (beekeeping season) *	Every 30 days (beekeeping season) **	NA
Brood pattern	Experimental observation	Brood spottiness rating. Scale 1–5	Every 21 days (beekeeping season) *	NA	NA
Drone presence	Experimental observation	Yes/no	NA	Every 30 days (swarming season) **	NA
Suppressed in ovo virus infection	Lab analyses	PCR data	Once every queen *	NA	NA
Viral diversity Deformed wing virus	Lab analyses	Sequencing data	Select number of samples within each country **	NA	NA
*Varroa destructor*	Lab analyses	Mites/100 bees	3 times a year (spring, summer, fall)	3 times a year (spring, summer, fall)	3 times a year (spring, summer, fall)
Deformed wing virus A	Lab analyses	PCR data	3 times a year (spring, summer, fall)	3 times a year (spring, summer, fall)	3 times a year (spring, summer, fall)
Deformed wing virus B	Lab analyses	PCR data	3 times a year (spring, summer, fall)	3 times a year (spring, summer, fall)	3 times a year (spring, summer, fall)
Acute bee paralysis virus	Lab analyses	PCR data	2 times a year (spring, fall)	2 times a year (spring, fall)	2 times a year (spring, fall)
Chronic bee paralysis virus	Lab analyses	PCR data	2 times a year (spring, fall)	2 times a year (spring, fall)	2 times a year (spring, fall)
American Foulbrood	Lab analyses	PCR data	Once a year (fall)	Once a year (fall)	Once a year (fall)
European Foulbrood	Lab analyses	PCR data	Once a year (fall)	Once a year (fall)	Once a year (fall)
*Nosema ceranae*	Lab analyses	PCR data	2 times a year (spring, summer)	2 times a year (spring, summer)	2 times a year (spring, summer)
*Nosema apis*	Lab analyses	PCR data	2 times a year (spring, summer)	2 times a year (spring, summer)	2 times a year (spring, summer)
Sacbrood virus	Lab analyses	PCR data	3 times a year (spring, summer, fall)	3 times a year (spring, summer, fall)	3 times a year (spring, summer, fall)
Black queen cell virus	Lab analyses	PCR data	3 times a year (spring, summer, fall)	3 times a year (spring, summer, fall)	3 times a year (spring, summer, fall)
*Malpighamoeba* *mellificae*	Lab analyses	PCR data	3 times a year (spring, summer, fall)	3 times a year (spring, summer, fall)	3 times a year (spring, summer, fall)
Foundationless frame	Management actions	Yes/no	When necessary	When necessary	When necessary
Drone brood removal	Management actions	Yes/no	When necessary	When necessary	When necessary
Brood layers	Management actions	Number	When necessary	When necessary	When necessary
Frames per layer	Management actions	Number	When necessary	When necessary	When necessary
Honey super	Management actions	Number	When necessary	When necessary	When necessary
Comb replaced	Management actions	Number	When necessary	When necessary	When necessary
Nutrition/sugar feeding	Management actions	Weight/volume	When necessary	When necessary	When necessary
Swarming prevention	Management actions	Method	When necessary	When necessary	When necessary
Queen introduction	Management actions	Reason and method	When necessary	When necessary	When necessary
Queen marking	Management actions	Colour	When necessary	When necessary	When necessary
Queen cell removal	Management actions	Number	When necessary	When necessary	When necessary
Colony split	Management actions	Yes/no	When necessary	When necessary	When necessary
Colony united	Management actions	Yes/no	When necessary	When necessary	When necessary
Colony feeding	Management actions	Volume/weight	When necessary	When necessary	When necessary
Honey harvest	Management actions	Weight/volume	When necessary	When necessary	When necessary
Varroa treatment	Management actions	Method	When necessary	When necessary	When necessary
Temperature	Weather (from weather service)	°C (Celsius degrees)	15 min	NA	NA
Wind speed	Weather (from weather service)	m/s	15 min	NA	NA
Humidity	Weather (from weather service)	% RH	15 min	NA	NA
Rainfall	Weather (from weather service)	mm/h	15 min	NA	NA

* added in 2021; ** added in 2022.

**Table 2:** Overview of the protocols used in the B-GOOD project. See the main text and the Supplementary Information S1 for more details.

Label	Protocol	Description	Tier 1/ Researchers	Tier 2/ Beekeepers	Tier 3 ****/ Beekeepers
P1	Queen & BIAS	Finding the Queen and checking Brood In All Stages	20210129	20220513	20220225
P2 *	Liebefeld	How to apply the Liebefeld method for counting bees, amount of brood and food resources	20220513		
P3	Top Photo Analysis	Analyses of colony sizes by taking pictures of brood from the top	20220513	20220513	
P4	Varroa	Counting natural Varroa mitefall to measure mite infestation level	20220513		
P5	Lab analyses	How to sample bees sent for Lab analysis for diagnostic purposes	20220513	20220513 **	20220225
P6	Atypical behaviour	Visually assess colony behaviour	20220204		
P7	Clinical signs	How to visually check colonies for clinical signs of disease	20220204	20220202	
P8 *	EFSA protocol	Performing the EFSA to estimate colony size, amount of brood and food resources	20220513		
P9	Drone eggs	How to collect drone eggs	20220513		
P10	Queen cell presence	Checking for colony cells and explaining the four different queen cell types	20220204	20220224	
P11	Brood pattern	How to measure brood pattern consistency	20220204		
P12	Data quality	Checking and cleaning up data on the BEEP app	20220204		
P13	Drone Presence	Checking colonies for presence of drone brood		20220513 ***	

* P2 and P8 were linked; partners were asked to choose either P2 or P8. Protocol P8 was added in 2021, year 2 of Tier 1; ** Split into two protocols; for sampling by beekeepers and for shipment by B-GOOD partners; *** Presence of drone brood for researchers in Tier 1 is included in the protocols for Liebefeld/EFSA; **** Tier 3 protocols were directly integrated into the workplan to increase user friendliness.

**Table 3:** The primers and probes used for the laboratory analyses. The table shows the forward (Fwd) and reverse (Rev) primers and probes of the honey bee viruses acute bee paralysis virus (ABPV), black queen cell virus (BQCV), chronic bee paralysis virus (CBPV), deformed wing virus (DWV; genotype A and B), and sacbrood virus (SBV), as well as the pathogens *Nosema apis*, *Nosema ceranae*, *Paenibacillus larvae* (causative agent of American foulbrood = AFB), *Melissococcus plutonius* (causative agent of European foulbrood = EFB), and *Malpighamoeba mellificae*. Analyses were performed according to standard protocols based on recent publications and on methods described by the European Reference Laboratory (EURL) for bee health.

Target	Primers (5′-3′)	Probe (5′-3′)	Reference
DWV A	Fwd: GCGGCTAAGATTGTAAATTG Rev: GTGACTAGCATAACCATGATTA	CCTTGACCAGTAGACACAGCATC	[50]
DWV B	Fwd: GGTCTGAAGCGAAAATAG Rev: CTAGCATATCCATGATTATAAAC	CCTTGTCCAGTAGATACAGCATCACA	[50]
ABPV	Fwd: CATATTGGCGAGCCACTATG Rev: CTACCAGGTTCAAAGAAAATTTC	ATAGTTAAAACAGCTTTTCACACTGG	[48]
CBPV	Fwd: CGCAAGTACGCCTTGATAAAGAAC Rev: ACTACTAGAAACTCGTCGCTTCG	TCAAGAACGAGACCACCGCCAAGTTC	[45]
*Nosema apis*	Fwd: CCATTGCCGGATAAGAGAGT Rev: CCACCAAAAACTCCCAAGAG	ATAGTGAGGCTCTATCACTCCGCTG	[47]
*Nosema ceranae*	Fwd: CGGATAAAAGAGTCCGTTACC Rev: TGAGCAGGGTTCTAGGGAT	CGTTACCCTTCGGGGAATCTTC	[47]
*Melissococcus* *plutonius* (EFB)	Fwd: TGTTGTTAGAGAAGAATAGGGGAA Rev: CGTGGCTTTCTGGTTAGA	AGAGTAACTGTTTTCCTCGTGACGGT	[46]
*Paenibacillus larvae* (AFB)	Fwd: TACGCTTTTCGATTCTCTG Rev: GTCTGTACTGAACCAAGTC	ATCTGCTTCCACTTGTTCACTCACCA	[49]
BQCV	Fwd: GGTGCGGGAGATGATATGGA Rev: GCCGTCTGAGATGCATGAATAC	TTTCCATCTTTATCGGTACGCCGCC	[51]
SBV	Fwd: AACGTCCACTACACCGAAATGTC Rev: ACACTGCGCGTCTAACATTCC	TGATGAGAGTGGACGAAGA	[52]
*Malpighamoeba* *mellificae*	Fwd: TATACAGATTGTGTAAAAGCG Rev: TTAGCCTCTATCTAACCTACC	TACAAGAGGATCTGCCCTATCAACTAT	[44]

## Data Availability

All work plans, protocols, and lists were either included in the main body of text of this manuscript or as supplementary information. The underlying data of the B-GOOD project would become publicly available through the B-GOOD Bee Health Data Portal (https://beehealthdata.org/login).

## Supplementary Materials

The following supporting information can be downloaded at: https://www.mdpi.com/article/10.3390/insects15010076/s1, S1: Tier 1 Workplan and protocols; Tier 2 Workplans and protocols; Tier 3 Workplan and protocols; S2: Table checklist.
