# Supplementary material for: Bridging the Gap between Field Experiments and Machine Learning: The EC H2020 B-GOOD Project as a Case Study towards Automated Predictive Health Monitoring of Honey Bee Colonies
Source: Insects. 2024 Jan 22;15(1):76. doi: 10.3390/insects15010076 (PMC10816039; doi:10.3390/insects15010076)
Supplement: Supplementary file 1 [file insects-15-00076-s001.zip › S1_tier 1_workplans and protocols.pdf]

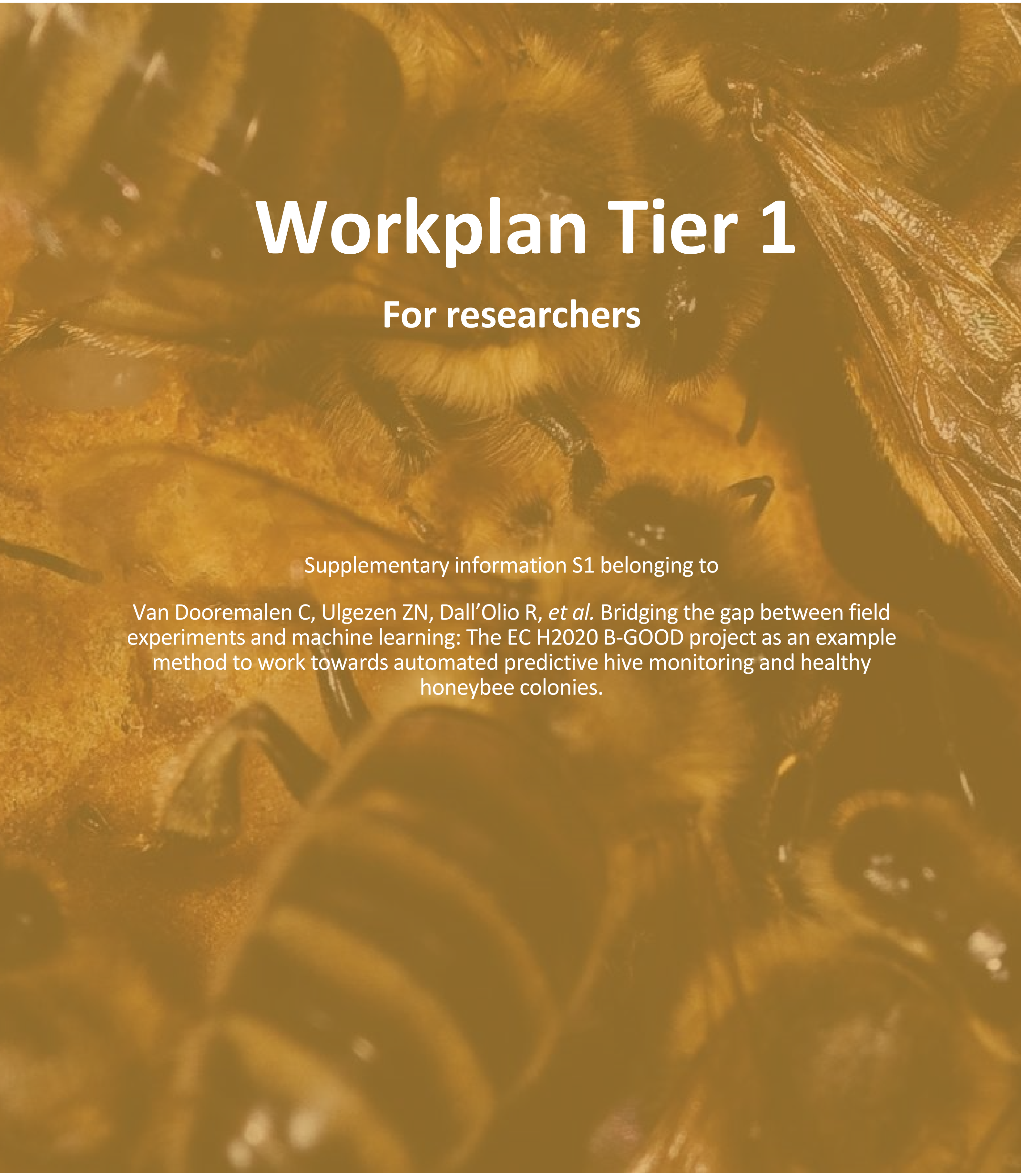

Workplan Tier 1

For researchers

Supplementary information S1 belonging to

Van Dooremalen C, Ulgezen ZN, Dall'Olio R, *et al.* Bridging the gap between field experiments and machine learning: The EC H2020 B-GOOD project as an example method to work towards automated predictive hive monitoring and healthy honeybee colonies.

This workplan was developed for B-GOOD project. B-GOOD stands for Giving Beekeeping Guidance by computational-assisted Decision making. The B-GOOD project received funding from the European Union's Horizon 2020 research and innovation programme under grant agreement number 817622.

B-GOOD workplan title: Workplan Mini-Apiaries B-GOOD WP1

Version: 20220204

B-GOOD Tier: Tier 1

Target group: Researchers

Comment: This is an exact copy of the workplan as it was used in the B-GOOD project

Workplan may be updated during the project period. Always ensure you have the latest version of the workplan.

Experiment Set-up

Eight partners with honey bee keeping facilities are involved in pilot study A (Table 1). The experiment will run for three years from January 2020 till April 2023. Each institute will participate in the experiment with eight (presumably) healthy colonies at their B-GOOD mini-apiary.

Mini-apiaries → 8 institutes x 8 colonies = 64 colonies

This workplan facilitates the methods and protocols for all preparations and measurements during the experiment. We will use classical methods of measuring bee health by visual inspection, where colony traits such as colony size and food resources are recorded in order to:

1) Determine the most important parameters for bee health;
2) Validate new technologies for automated measurements of health status.

For automated measurements, the BEEP system (BEEP app and BEEP base) will be the first technology to be used and validated in TIER1, starting from the beginning of experiments, as it has a TRL high enough (>6) to enter the validation process. BEEP is a system built to support beekeepers, where observations of the hive can be digitally registered using a record keeping app, complemented by automatic observations using sensors. Other new tools originating (most likely) from WP2 or WP3 may be entered into TIER1 of WP1.

Table 1: List of participants.

Participant organisation name	Abbreviation
Universiteit Gent	UGENT
Stichting Wageningen Research	WR
Institut National de la Recherche Agronomique	INRAE
Martin-Luther-Universitaet Halle-Wittenburg	MLU
Universitatea de Stiinte Agricole Medicina Veterinara Cluj Napoca	UCLUJ
Universidade de Coimbra	UCOI
The Nottingham Trent University	TNTU
Universitat Bern	UBERN

BEEP System

BEEP app

See Appendix 1 for information on how to register in the BEEP app. We will use the BEEP app to collect and store data in a standardized way for:

1. Meta data entry on the apiary level, e.g. location, colony numbers, type of hives, number of brood boxes.
2. All management actions related to beekeeping that are done throughout the period January 2020 to April 2023, e.g. adding or removing of brood boxes or honey supers, splitting colonies, queen replacement, feeding (see 'Beekeeping management'). As these actions are very dependent on the location, inspection sheets should be made by the user.

3. All experimental observations, e.g. colony size using top photo (see 'experimental observations'). For the experimental observations standard inspection sheets are prepared. The inspection sheets can be found in the BEEP app (Figure 1). In the section 'experimental observations', you can find information on when and which protocols and inspection sheets should be used throughout the measurement period January 2020 – April 2023.

Please see BEEP manual for instructions on data entry and how to make inspection sheets for management actions.

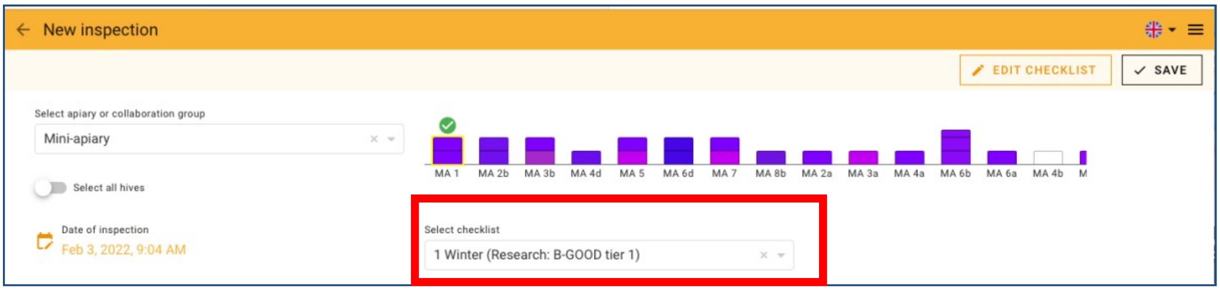

Figure 1: The section for selection of checklist for data entry. Select the appropriate checklist for data collection depending on type of experimental observation and timing of data collection (see Appendix 2).

We will make use of the 'web app' of the BEEP platform to log all information on the apiaries. Web app means that you can open the app on your mobile phone, tablet and computer and that you do not have to download anything. The app is also automatically updated. In the manual you can find out how you can open the BEEP app on your phone or tablet. You only need one account and with that account you can access BEEP on multiple devices.

All data must be recorded electronically. Use the appropriate fields to enter the data and only use the notes field if there are no other options, in order to ease data analysis. Notes must be entered in English only. If needed, the BEEP app categorisation will be extended. When you click the save button in the BEEP app, the data will be stored on the BEEP server. Each participant will have continuous access to their own collected and stored data. WR will have access to all data collected from the mini-apiaries and will process the data for further use together with the BEEP team.

At each hive inspection, the BEEP app on your phone or computer must be connected to the internet to send data to the server. The BEEP app automatically registers the date and time when you enter data into the system. Information to the app can also be added at a later date. However, it is important to change

and correct for the date of inspection, and adjust it to the original moment of data collection if data is entered at a later date (Figure 2).

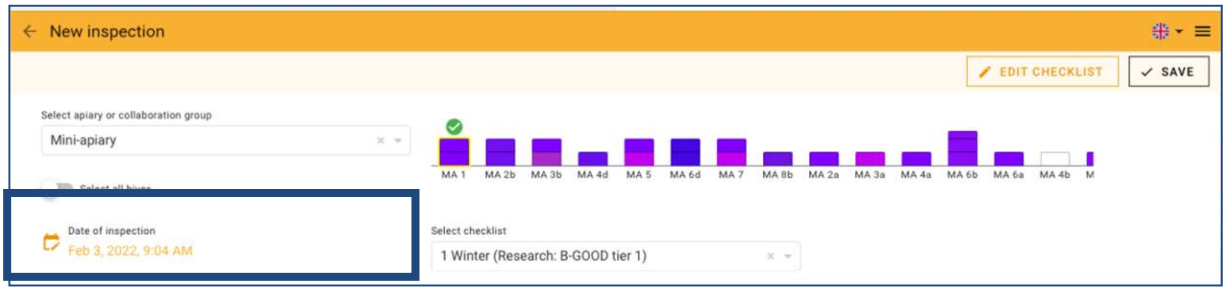

Figure 2: The section for entry of date of inspection (observations and actions) in the BEEP app. If data is entered at a later date adjust it to the original moment of data collection.

BEEP base and sensor placement

At the start of the experiment, you will get 8 BEEP bases (Figure 3) to install under your selected hives. During the experiment we will continuously monitor hives with the BEEP base, which measures weight, temperature and sound in the hive. BEEP will provide an installation manual that includes information on the set-up of the BEEP base (for online version click here). See below for sensor placement in the hive.

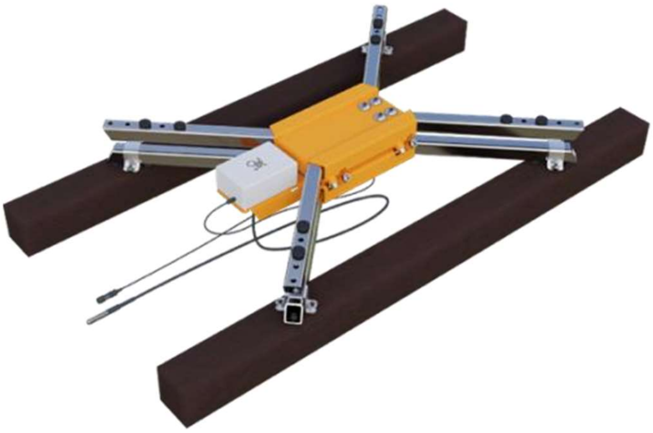

Figure 3: The BEEP base that will be placed under each of the 8 hives in your apiary after you installed the colonies. Note that the computer (white box) is placed to the side. The BEEP base dimensions are such that most hives fit well and stable when the BEEP base is placed this way. Turning it 45 degrees is not preferred.

Scale. A weight sensor is at the centre of the steel construction of the BEEP base. The BEEP base needs to be placed underneath the hive for continuous weight measurements (Figure 4).

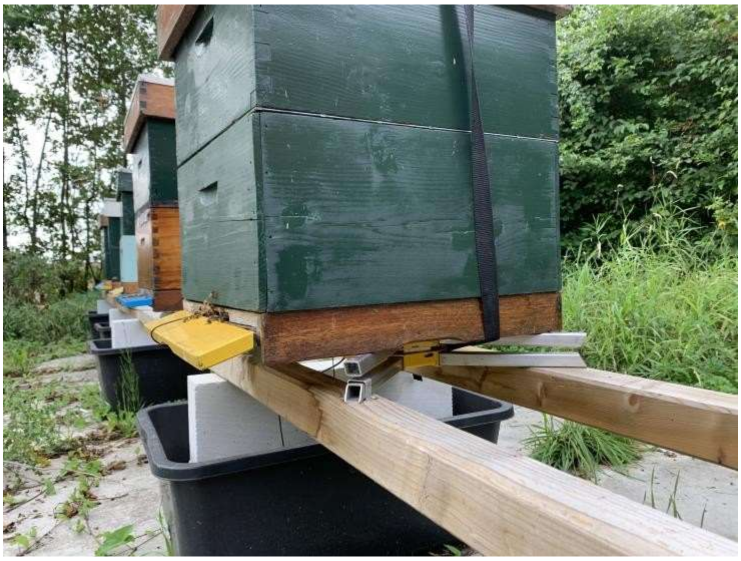

Figure 4: Placement of weight sensor underneath the hive.

Thermo-sensor. A thermo-sensor is connected through a cable to the BEEP base, and will be used for continuous temperature measurements. The sensor needs to be placed in the brood box, on top, between the mid-frames (Figure 5). If the hive has several brood boxes, and there is a queen excluder, keep the temperature sensor in the box with the queen. If there is no queen excluder, always keep the sensor in the top brood box.

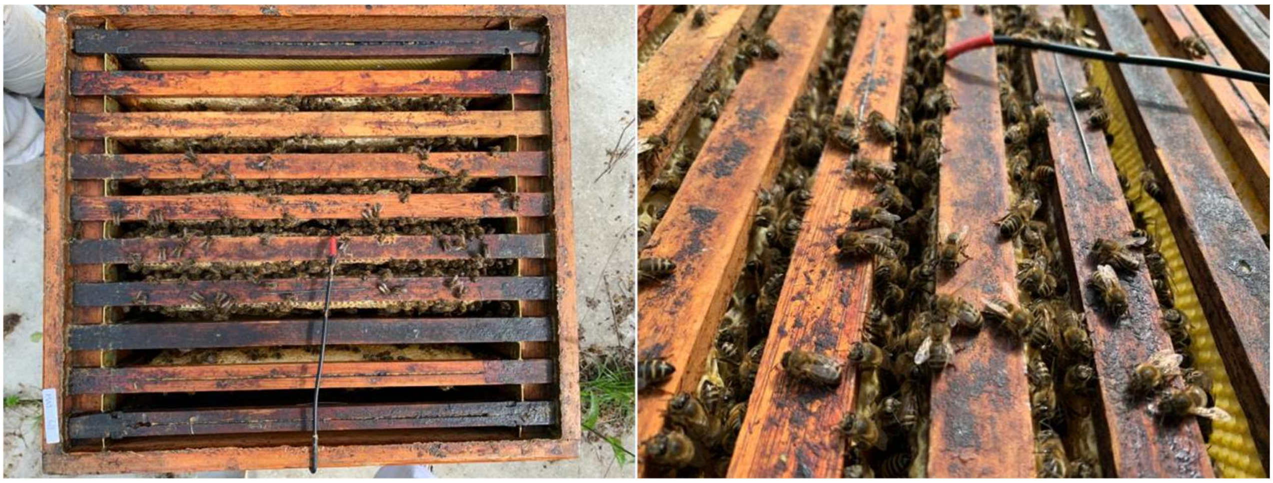

Figure 5: Placement of temperature sensor in the hive. It is approximately 9 cm from the red tape to the tip of the temperature sensor.

Microphone. A microphone is connected through a cable to the BEEP base, and will be used for constant sound measurements. The sensor needs to be placed at the center of the hive bottom, facing the back of the hive, opposite to the hive entrance (Figure 6).

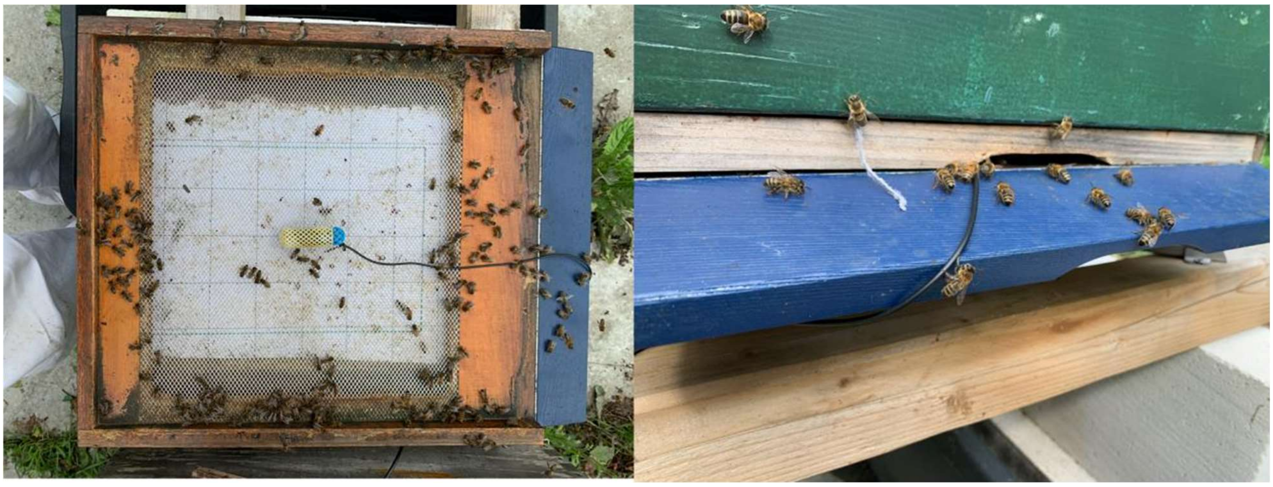

Figure 6: Placement of microphone in the hive. The cable can be placed through the flight entrance.

Beekeeping management

The basic principle of management actions within Pilot A will be to: maintain healthy colonies; no traveling (unless with offspring), while at the same time respecting the nature of the bees; and providing care accordingly.

We will not provide you with standard beekeeping protocols, but suggest general guidelines and conditions required for actions on several beekeeping practices, such as controlling swarms and varroa mites. Participants should make 'local' decisions on timing of practice, depending on colony status, phenological state and climate of country. If somehow, you cannot/will not meet the suggested guidelines, we ask you to discuss with WR, preferably before taking action (using the Teams WP1 channel). Always register all your decisions and argumentations, actions, interventions and observations in the BEEP app. As we will only give guidelines for beekeeping in TIER1 of WP1, no protocols are being provided other than the text below.

Reproduction (queen renewal) and swarm control

As soon as drone brood starts to occur in the colony, check for queen cells every time the hive is opened. If present, remove queen cells and keep a record in the BEEP app of removal and number of cells (in Inspection under Bee colony > Queen > Cells > Removed cells) with the correct inspection date. When the colony is growing and there is congestion in the hive, swarming tendencies can be reduced or prevented by

1) providing extra space to the hive,
2) by making 'cuttings',
3) by splitting.

Cuttings and splitting will help to

- Facilitate treatment against *V. destructor* (see '*Varroa Control*'),
- Create offspring or 'spare parts' for fixing main colonies or queen renewal

We suggest that each participant prioritizes step 1-3 according to the honey flow to maximize honey yield (for NL that would be 1, 2, 3, and postponing splitting till after flowering of Linden). The timing for swarm control intervention can be determined by using an indicator frame; add an empty brood frame (so no comb or foundation) at one of the most outer sides of each brood box in the hive. The colony needs more space when bees start building on this empty indicator frame. Always record instances of adding space, 'cutting', splitting, merging, and replacing, locations of 'splits' and the identity of the remaining queen in the hive after merging in the BEEP app (in Inspection under Bee colony > Splitting colony and Bee colony > Uniting colonies. Adding or removing space can be entered in Overall > Notes).

Ad 1. Additional space. Provide extra space to the colony by adding a second brood box on top of the first one. If possible, we suggest doing this as follows. Place the queen with two frames of brood (young open brood and/or eggs) from the original brood box into the new box, above a queen excluder. Fill up the rest of the box with new frames of comb foundation. Swarming tendency will be greatly reduced by this extra space and is further prevented by the queen excluder, as the queen will be unable to join the swarming bees. In case of heavy honey flow, an alternative could be to first place an extra honey super on top of the hive and then if the colony keeps on growing to add the second brood box in between the honey super and the brood box as described above.

Ad 2. Cuttings. When the second brood box becomes overpopulated[1], indicated by bees building on the empty indicator frame, we suggest to make a cutting[2]. Cutting will lead to a new colony with a young queen. For cuttings move one or two frames with a lot of capped brood and a few eggs or very young larvae to a new brood box. Place one or two frames with food stores next to the brood frames. All 2-4 frames should be well populated by worker bees (but NO queen). Fill up the rest of all empty spaces with new frames of comb foundation. Move the cutting to another location in the apiary or to another (temporary) location or a mating area. Make sure there are drones in the area for mating. The bees in this cutting will raise a new queen. While it is unlikely that the cutting will swarm due to its small size, to be certain, check the hive after 13 days. When there is a new queen, remove the remaining queen cells. If the 'old' colony keeps on growing, this process can be repeated. The BEEP base should be left underneath the original 'old' colony at the B-GOOD apiary.

Ad 3. Splitting. After the main honey flow (ca. mid-summer), if the original 'old' colony is large enough it can be split into two colony parts. Remove the queen from the top box (see Ad1.) and place her in the bottom box. Move the top brood box to another location in the apiary or to another (temporary location, with drones), or a mating area. That split part (top box) will raise a new queen. Check this top box after 13 days. When there is a new queen, remove the remaining queen cells. Keep the BEEP base underneath the split part (bottom brood box) that remained at the B-GOOD apiary with the 'old' original queen. As long as no merging is needed, keep the original queens in the B-GOOD hives.

[1] We suggest to keep the colony at a maximum of 2 brood boxes and 1 honey super. This is because of the measurements that need to be done each month and the labor intensity required. Change the protocols and measurements accordingly when you exceed the suggested number of boxes in use.

[2] Like making cuttings of a plant: cut off a small part of the plant, put it in water till it grows roots, put it in soil and you have got a new (baby) plant.

Depending on the swarm control actions that have been performed for each colony, additional actions need to be taken in autumn. However, the specific actions required should be considered based on the management at the apiary level. Management actions to consider and how to perform them are listed below.

Reducing the number of brood boxes. Before winter (at winter preparation time), try to reduce the number of brood boxes as much as possible. This is for thermoregulation efficiency. Compact the colony into (preferably) one brood box when possible, with frames of food and bees, and remove the surplus brood box and frames.

Merging colonies. Weak colonies can be merged before and/or after winter to strengthen colonies. In colonies that will be merged, if there are no visible problems with either one of the queens, the choice of queen can be left to the bees. For merging, we suggest to use the newspaper method.

Replacing colonies. In the unfortunate event of colony loss, the dead colony can be replaced by a 'spare part' colony (cuttings/split part with newly mated queen), preferably with a related one ('daughter' or 'granddaughter'). If there is a sudden loss of queen, e.g. due to experimental handling of the colonies, the 'spare part' can be (re)merged with the queenless 'old' colony. In cases where there are no spare parts available, and the colony cannot replace the queen by building emergency cells (no open brood), the queen will not be replaced for the remainder of the year. Depending on the timing of loss (year 1 or 2), the empty place can be filled, by using spare parts of the remaining colonies, but that colony should get a new number (e.g. #9, daughter of #…).

Varroa Control

To keep the colonies as healthy as possible, varroa control is necessary. As thresholds for control are difficult to determine, we opted for a standardized control method (better-safe-than-sorry-method) for TIER1. Oxalic acid is suggested for varroa control, and for maximum effectiveness treatments should always be in the absence of capped brood. For each colony, record dates of oxalic acid treatment, amount used, and type in the BEEP app (in Inspection under Disorder > Varroa > Treatment).

Summer treatment. Colonies will be treated against Varroa with oxalic acid spraying in summer (concentration 30 g oxalic acid dihydrate in 1 L water). With 1L you can spray approx. 12 full brood boxes. To see how to spray a small colony: link.

Timing in summer. The timing of oxalic acid treatment for colonies can vary depending on actions taken for reproduction and swarm control. There are several methods of spraying that can be applied listed below.

1) **Cutting.** The 'cutting' colony with the young queen can be sprayed 21-24 days after splitting, when all of the brood has emerged and only open brood (or no brood yet) is present.

2) **Splitting.** When the brood boxes are separated (making two separate colonies), both colonies will have a period without brood. This will create an opportunity for oxalic acid spraying in both colonies (top and bottom brood box), but at different time points. The colony with the

old queen (bottom brood box) will be broodless when the boxes are split. Therefore, it can be sprayed with oxalic acid at the moment of splitting or latest within one week. The new colony (top brood box), can be sprayed 21-24 days after splitting when all of the brood has emerged and only open brood (or no brood yet) is present.

3) **No cuttings or splitting.** If resources are scarce, colonies may not have swarming tendencies, preventing splits and/or artificial swarms. In such cases the queen can be caged for 21-24 days and the colony can be sprayed when all of the brood has emerged and only open brood (or no brood yet) is present.

Winter treatment. Colonies will be treated against Varroa with oxalic acid trickling in winter (ca. December). The timing of oxalic acid treatment should be when (1) there is no brood and (2) the colony has formed a cluster. 5ml of oxalic acid solution should be trickled per seam of bees (35g/L oxalic dihydrate in sugar syrup). Frames should not be removed from the hive in winter due to low ambient temperatures. Brood production may continue in countries where the weather is favourable all year long. In such cases colonies can be treated with oxalic acid spraying in winter by caging the queen. To see how to trickle a colony: link (sorry Dutch written text available only in between the movie)

Nutrition

Colonies may be supplemented with sugar dough when there is a shortage of resources. In cases of cuttings and splitting, always provide sugar dough to the part that will get the new queen (for information on splitting see section 'Reproduction and swarm control'). In fall, before the wintering period, supplement bees with bulk e.g. APIinvert (sugar syrup, contains 70% fructose/glucose and 30% water), as much as they want to uptake.

Honey harvest

If honey is harvested, then record how much honey is harvested per hive and the date of harvest. Measuring honey harvest can be done by weight calculations. Pre-weigh the honey super before placing onto the hive. Place a queen excluder below the honey super to restrict brood in a separate hive body. Remove and weigh the bee-free honey super and subtract values to determine honey yield. Alternatively, report the kg of honey after harvest for each hive separately and record this the BEEP app (in Inspection under Production > Honey). You may exclude the weight of wax seals from the calculations (automatically adding that weight to the honey harvest)

Queen marking

Before the start of experiments and at each supersedure the queen should be marked. Marking helps with identification and tracking the age of the queen. Queens can be systematically labelled with a colour code, such as international code of colour (Table 2). Alternatively, queens can be labelled with a unique identifier (e.g. Opalith discs with numbers). It is fine to use any kind of queen marking as long as the queen can be tracked. Enter the queen marking and colour in the BEEP app (click on the hive to open settings, scroll down for Queen details). In the overview of hives per apiary, you will see the queen colour displayed.

Table 2: International colour code used for marking queens by year of birth (Human et al. 2013).

Colour	Birth years ending in
White	1 or 6
Yellow	2 or 7
Red	3 or 8
Green	4 or 9
Blue	5 or 0

Data collection: experimental observations

Data collection methods throughout the experiment are detailed below (with the related protocol number between brackets). Please see Appendix 2 for an overview of measurements over time, the relevant protocol for each method and more information on data collection. Detailed information about methods for data collection are provided in the protocols. Try combining activities to minimize colony disturbance.

Presence of queen and brood (P1)

Every 20 days starting from the end of the winter period till the end of the beekeeping season (essentially, the period of honey bee foraging activity), check the hive comb surface for the presence of queen and of all stages of brood (BIAS, brood in all stages). The presence of worker brood gives information on queen fecundity, viability of worker force and the ability of the colony to rear the eggs until adulthood. Keep a record in BEEP app of queen presence, brood presence and queen supersedure (in Inspection under Bee colony > Queen and Bee colony > Brood).

Estimation of brood size/food resources/colony size- EFSA method/Liebefeld (P8 or P2)

The amount of food resources (honey and pollen), brood, and colony size (amount of bees) are key determinants of colony development and survival. For the estimation of these parameters please consider using the EFSA protocol, which uses digital photography and photo analysis (P8). This is the preferred method for machine learning in WP5. If EFSA is too laborious and is found to be too stressful on colonies, mini-apiaries can switch to the Liebefeld method. Frequency of measurements should take precedence over method. However, consistency is also important, therefore within a bee season please stick to the same method. Please notify WR if you would like to switch between methods.

Measurements should be done every 20 days, starting in spring (when bees start to forage), until the end of the bee season (before the overwintering). It is important that data is collected within 21 days of the last visit, in order for the sampling frequency to cover the developmental time of workers (21 days from egg to adult). However, hive monitoring should be adapted to weather conditions (not possible when raining, and precautions should be taken when temperatures are low (<14 degrees). If you lack the resources, alternatively you can use the Liebefeld method (P2).

These colony traits are estimated with each measurement:

- Colony size (bees)
- Pollen stores

- Honey (sealed)
- Capped brood (pupae)
- Open brood (larvae)
- Eggs
- Drone brood

Top photo analysis (P3)

Colony size will also be estimated by taking a photo of the topside of the hive. There are several benefits of this method as it is less invasive, faster, and easier, mainly: (1) it can be used during winter when temperatures are too low for removing frames (2) it is easy enough for beekeepers to apply. This measurement should be done once a month during the wintering period (preferably first week of the month), and every 20 days during the beekeeping season. At times of temperatures below 5°C, it may be better to postpone or even skip this measurement.

Mite infestation level (P4)

Mite infestation level of each colony will be measured by quantifying naturally falling mites. Preferably, the infestation levels should be counted once a week, throughout the beekeeping season. However, if not possible (apiary in remote area/difficult access), it is acceptable to reduce the frequency of this measurement to 1x a month. If you measure mite fall during winter as well, please also enter this data in the BEEP app.

Although we apply standard Varroa control and no Integrated Pest Management (IPM)[3], it is still important to measure mite infestation levels of the hive as Varroa is considered to be one of the most harmful stressors for honey bees and treatments against it are not 100% effective. Therefore, before, during and after varroa treatments, we urge to increase the number of measurements as such that they will give you a good indication of the effects of the mite treatment. In such cases, frequency of counting should depend on the treatment method, the duration of treatment used, but potentially also the remoteness of the apiary.

Sampling bees for lab analyses (P5)

For lab analysis on genotyping and diseases, samples of bees should be collected three times a year. First in spring, when the bees start to forage; second time in summer, when the colonies have reached their maximum size; and a third time in autumn, before the over-wintering. As spring, summer and autumn will be shifted between participating institutes, the sampling moments need to be adjusted by participants depending on the climate of country. The first (spring) visit should take place as soon as major pollen producing plants are flowering. Look out e.g. for the genus *Salix (S. caprea or S. cinerea*). The spring visit should take place within three weeks from the moment these plants start flowering. The second (summer) visit should take place about 2 weeks after midsummer, or if known, the moment of peak colony size that naturally occurs in region. The third (autumn) visit should take place before it gets too cold to open the

[3] IPM is the use of available pest control techniques and appropriate integration of measures for reducing the development of pest population, while minimizing the risks to human health, beneficial non-target organisms, and the environment

colonies (< 10°C). Depending on the location of the apiary, the visiting time can be from around the end of September until the end of November. The first and the third sampling moments should coincide with the start and stop of the intensive colony size measurements (i.e estimation of brood size/food resources/colony size)

Analysis on genotyping will be done by Universiteit Gent (UGENT), Belgium and analysis on diseases will be done by FriedrichLoeffler Institut – Bundesforschungsinstitut für Tiergesundheit (FLI), Germany and Sciensano (SCIEN), Belgium. Part of sample material will be shared with INRAE, France for development of an LFD for virus in WP2. Diseases of main interest are: Varroa mites, Deformed Wing Virus (DWV), *Nosema spp.*, American foulbrood (AFB), European foulbrood (EFB), Acute Bee Paralysis (ABPV) and Chronic Bee Paralysis (CBPV) (see Table 3 for the timing of analyses). If colonies are suspected to have Sacbrood virus (see 'Clinical signs of disease'), then this disease will also be included in the lab analyses. If there is a particular disease suspicion at the moment of sampling (that will not be measured according to Table 3, like sacbrood), please indicate the suspicion on the individual samples. as well as in the BEEP app.

Table 3: List of diseases that will be analysed by FLI and SCIEN and timing of sampling by institutes. The moment of sampling is same as the moment for the Liebefeld method (see 'Estimation of brood size/food resources/colony size'). Therefore, we suggest that you combine these two activities in the field.

Disease	Sampling
Varroa	3 x a year
DWV-A and -B	3 x a year
Nosema	spring & summer
AFB	autumn
EFB	autumn
ABPV	autumn
CBPV	spring
Sacbrood	clinical signs of disease

Atypical worker behaviour (P6)

Atypical behaviour by workers is one of the first signals of diminished health within the colony. This indicator will be measured by visual inspection of the hive every 20 days during beekeeping season

Clinical signs of disease (P7)

During the beekeeping season, check the hive every 20 days for clinical signs of disease. If you have the suspicion that a disease is present in the hive, record the information in the BEEP app (in Inspection under Disorder > Type). Potential diseases that you may observe in colonies are: varroosis (Varroa mites), AFB (*Paenibacillus larvae*), EFB (*Melissococcus plutonius)*, nosemosis (*Nosema* spp.), ABPV or CBPV, Black Queen Cell Virus, DWV, Sacbrood virus and maybe (but hopefully not) small hive beetle (*Aethina tumida*).

Sampling drone brood eggs (P9)

The 'suppressed in ovo virus infection' (SOV) trait describes the virus free state of drone eggs. Recent research found that this trait is heritable and that colonies expressing this trait are more resilient to virus

infections as a whole with fewer and less severe DWV infections in most developmental stages, especially in the male caste (De Graaf, et al. 2020).

Information on the SOV trait of each hive in the mini-apiaries will be linked with the overall health status index and the pathogen prevalence by the University of Ghent. In order to do so, 10 drone eggs should be collected from each hive in the mini-apiaries.

Samples will be collected once a year, combined with the spring visit for sampling bees for lab analyses. If drone brood is not present in some colonies during spring, these colonies should be sampled the next visit that drones are present. In case the queen is replaced, new samples should be collected from the moment that drone eggs are present.

Queen cell presence (P10)

Presence of queen cells in colonies provides insight on reproduction (swarming tendency) and/or queen quality. Once a month during the beekeeping season, check colonies for presence of queen cells. Indicate in the BEEP app the type of queen cell present (queen cup, emergency cell, swarm cell, supersedure cell). If queen cells are removed, please include this information as a management action.

Brood pattern consistency (P11)

Brood pattern consistency gives information about the quality of the brood in a colony. If the brood is 'spotty', this may suggest the presence of disease, or low sperm quality. This measurement will be every 20 days during the beekeeping season.

Colony mortality

Information on colony mortality and observations on dead colonies should be recorded. A colony is considered dead if 1) the hive is absent of any living bees 2) the colony is too weak to recover in spring because (i) less than two frames are occupied by winter bees or (ii) the queen is dead and the hive cannot replace queen by building emergency cells (no brood). In case of

- 1 → replace by related 'spare part' colony
- 2i → merge with 'spare part' colony (max 2 attempts)
- 2ii → replace by related 'spare part' colony

See also the section 'Reproduction and swarm control' for information on merging and replacing colonies.

Overall impression

Information on the overall impression of the health of each colony will be collected throughout the whole year. There are three different categories in the BEEP app for the health status (Figure 7). Description or definition of categories will not be provided as this is a subjective measurement by the observer. Entry of data should be done at least once a month, but preferably reduced to every 20 days during the beekeeping season. This measurement is included at the end of each inspection sheet (see Appendix 2). If you use multiple inspection sheets during one inspection, filling in the overall impression on only one of the inspection sheets will suffice. Where the data annotation (see section 'Data annotation for machine

learning') uses 6 Boolean criteria to subjectively determine health status, the overall impression uses only 1 criterion, but with 3 possible values. Both levels of complexity need to be determined, for different purposes.

This measure is included because:

- If the health status of a colony changes, this measure allows us to go back in time and see if the beekeeper confirms this status change (assuming the beekeeper will know; TIER1 may be more reliable than TIER2 or 3).
- From a social science perspective, to test if there is a positive learning curve for estimating colony (health) status.

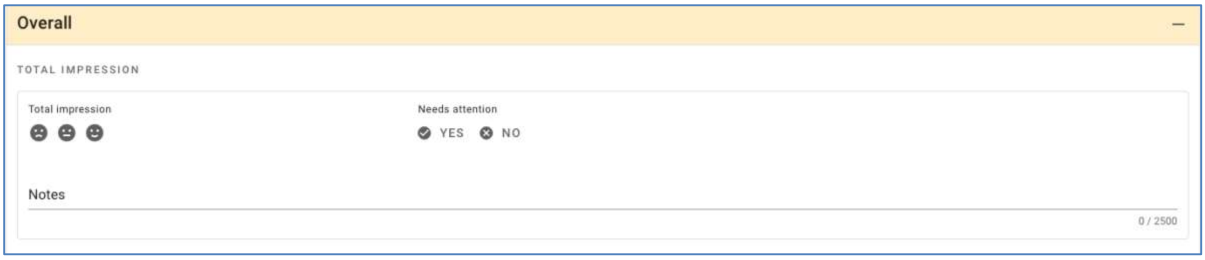

Figure 7: Data entry for overall impression on the health of colonies in the BEEP app.

Weather data

Weather data needs to be collected at each apiary. Ideally, we would like to collect hourly data on wind velocity and direction, air temperature, air relative humidity, rain and solar radiation.

For apiaries that have a weather station installed, BEEP will support with data collection. It is preferred that data is transmitted by the weather station onto the internet automatically and that the supplier provides automatic technical access to the data via e.g. an API (application programming interface).

If you do not have a weather station, you can purchase one. An affordable station which can send data wirelessly and works on batteries is the Meteohelix by Barani Design. See link here. When choosing for this station, please include the rain gauge.

If a weather station is not an option, you can also use a localized interpolated weather service that gives information on weather conditions (and forecasts). This is however, less precise than local weather stations.

Data annotation for machine learning

In order to do machine learning, WP5 needs the data to be annotated. For the machine to learn about colony status, we need to tell it what the health status of the colony is. This means that each time data is collected in WP1, the data needs to be linked to subjective information for it to be useful in WP5.

WP5 has selected the Healthy Colony Checklist for the annotation to enable comparison with Braga et al. 2020 (link). BEEP has prepared a special inspection sheet to be used each time data is entered in the BEEP app (Figure 8, inspection Health). Similar to the *Overall impression*, a description or definition of categories

will not be provided as this is a subjective measurement by the observer (an experienced beekeeper assessment). Entry of data should be preferably every 20 days during beekeeping season, and at least once a month throughout the year. At some time points you will have more information available (e.g. after doing colony parameter estimates) and can give a more reliable beekeeper assessment, compared to other time points (e.g. top photo during winter). Both types of time points are valuable.

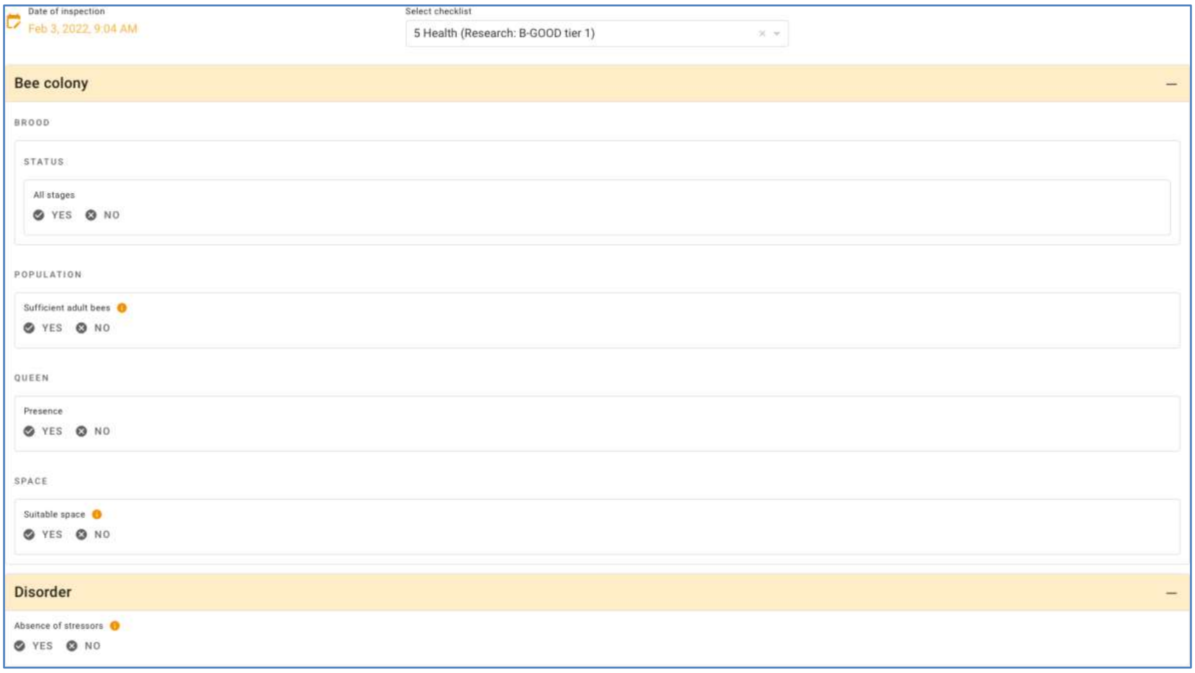

Figure 8: Part of the data entry for data annotation in the BEEP app (inspection sheet Health).

Data quality and cleaning (P12)

In order to detect deviation in data collection in an early stage, from 2021 onwards, WP5 will download reports of entered data frequently and give feedback to WP1. Please follow protocol P12 on data quality and cleaning for efficient workflow.

Acknowledgments

The authors of this workplan thank the B-GOOD Tier 1 and Tier 2 partners for their constructive feedback during the project duration to optimize the content for high quality data collection in the project. The B-GOOD project received funding from the European Union's Horizon 2020 research and innovation programme under grant agreement number 817622.

B-GOOD partners:

This workplan was developed for B-GOOD project. B-GOOD stands for Giving Beekeeping Guidance by computational-assisted Decision making. The B-GOOD project received funding from the European Union's Horizon 2020 research and innovation programme under grant agreement number 817622.

Appendix 1 – BEEP app registration

To start using BEEP for the B-Good project, please follow these steps:

Register in BEEP

- REGISTER: Go to the BEEP app, using a web browser on a computer or alternatively on a mobile phone via this link for the English version. As a new user, click on the login screen on 'No account yet? Register as a new user'. Register with your work email address and follow the instructions. See the login support article for more information on this step.
- APIARY: When logging in for the first time, you will see the 'Create new apiary' screen where you can add the B- GOOD apiary details. When you are done, click on 'Create new apiary' button to save the data. See the Create a new apiary article for more information on this step.
- HIVES: Open the apiary you created. You can change the settings per hive, by clicking on the hives. You can change the configuration and enter the details on the queens per hive. See this article for more information on this step.
- INSPECTIONS: By clicking on the pen icon under each hive, you can add inspections for that hive. This is also further described here.
- RESEARCH: An important step is to link your account to the B-GOOD research program. You only need to do this once. You can click on 'Research' in the menu on the left and select the B-GOOD program by following the on-screen instructions (Figure 9). This way the data can be accessed for analysis in WP1.

COLLABORATION: Multiple people can edit the data for the mini apiary. You can see here how you can set this up for your group.

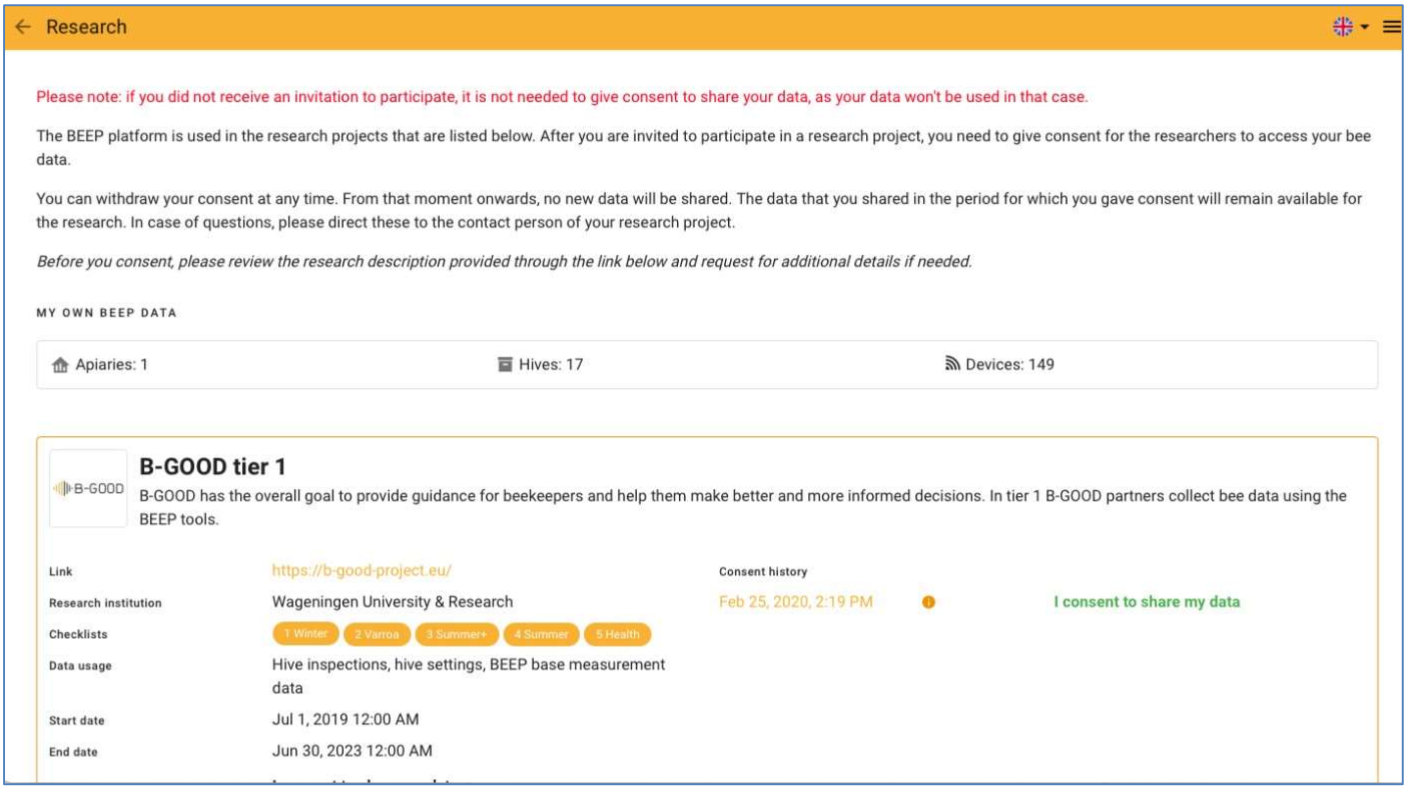

Figure 9: After creating your account on the BEEP platform, you can link your account to the B-GOOD programme by clicking on 'Research' in the menu.

Appendix 2 – Overview of experimental observations over time

Table 1: Overview of experimental observations over time. The protocol number, the timing (frequency) of measurements and the months in which they are to be performed are provided for each activity. Coloured cells show in which months experimental observations are expected to be performed. Five inspection sheets were prepared for use in the BEEP app, covering the different protocols: 1 Winter; 2 Varroa, 3 Summer+, 4 Summer, 5 Health.

			**Months**											
**Experimental observation**	**Protocol**	**Timing**	*Jan*	*Feb*	*Mar*	*Apr*	*May*	*Jun*	*Jul*	*Aug*	*Sep*	*Oct*	*Nov*	*Dec*
Presence of queen & brood	P1	every 20 days*												
EFSA/Liebefeld	P8/P2	every 20 days*												
Top photo analysis	P3	1 x month**												
Mite infestation level	P4	1 x week												
Sampling for lab analyses	P5	3 x year *												
Atypical worker behaviour	P6	every 20 days*												
Clinical signs of disease	P7	every 20 days*												
Sampling drone brood eggs	P9	1 x year *												
Queen cell presence	P10	every 20 days*												
Brood pattern consistency	P11	every 20 days*												
Overall impression	NA	1 x month **												
Data annotation	NA	1 x month **												
Colony mortality	NA	1 x month**												
Data quality and cleaning	P12	1 x month												
**BEEP app inspection sheets to be used**			**1, 5**	**1, 5**	**1, 5**	**2, 3 5**	**2, 4 5**	**2, 4 5**	**2, 3 5**	**2, 4 5**	**2, 4 5**	**2, 3 5**	**1, 5**	**1, 5**

** The months may vary between institutes, dates are only provided as an outline, and to represent activities that can be combined. Participants should make 'local' decisions on timing of data collection, depending on colony status, phenological state and climate of country. See workplan section on experimental observations for more details.*

*** Should be done 1 x a month during the winter period, but should preferably be reduced to every 20 days during the bee season.*

]

Queen and BIAS

For researchers

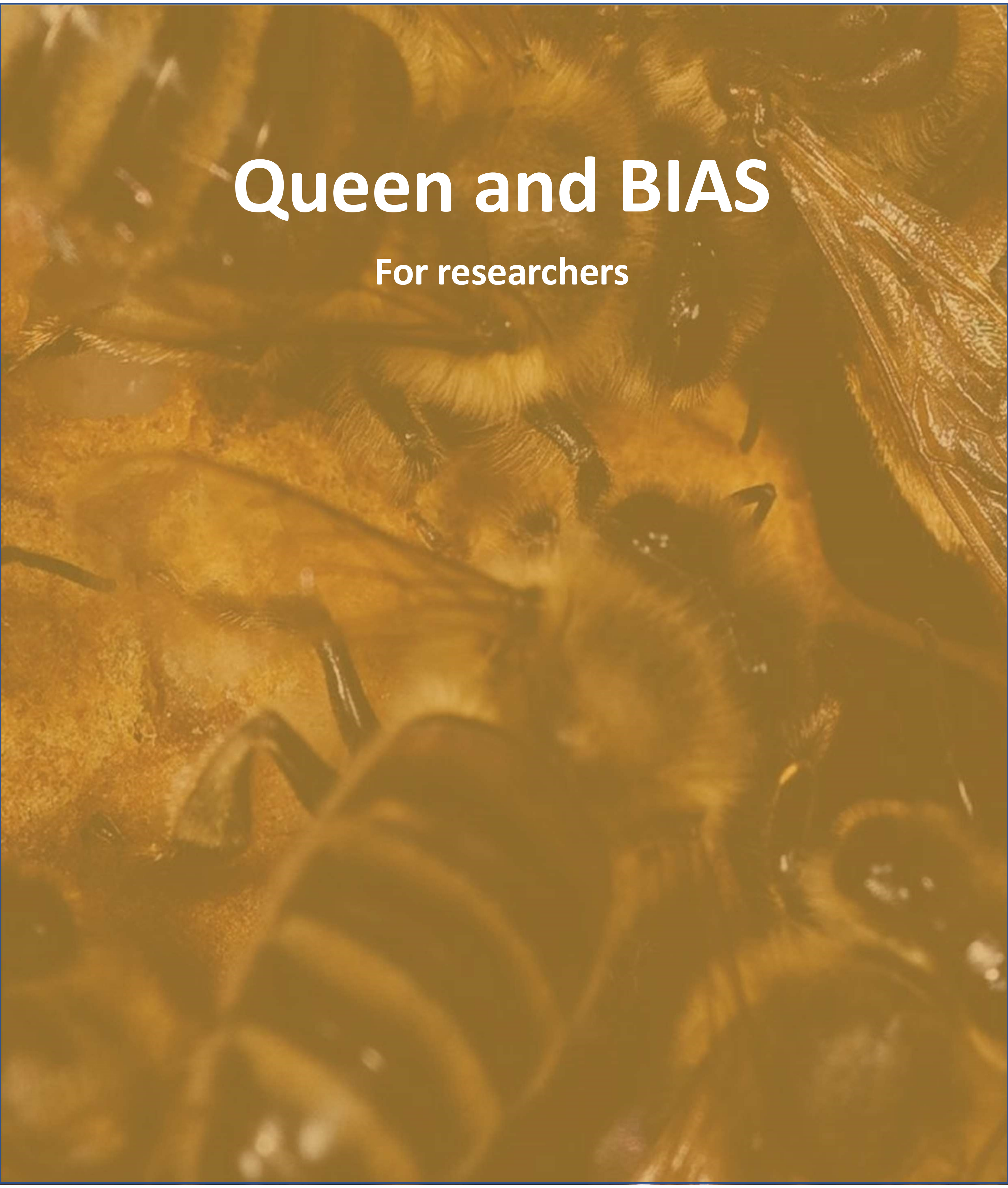

This protocol was developed for B-GOOD project. B-GOOD stands for Giving Beekeeping Guidance by computational-assisted Decision making. The B-GOOD project received funding from the European Union's Horizon 2020 research and innovation programme under grant agreement number 817622.

B-GOOD protocol title and code: Queen and BIAS (P1)

Description: Finding the queen and checking brood in all stages

Version: 20210129

B-GOOD Tier: Tier 1

Target group: Researchers

Presence of the queen and brood

Every 20 days starting from the end of the winter period till the end of the beekeeping season (essentially, the period of honey bee foraging activity), check the hive comb surface for the presence of queen and of all stages of brood. The presence of worker brood gives information on queen fecundity, viability of worker force and the ability of the colony to rear the eggs until adulthood.

Field methods

- Open a colony and sequentially remove frames from the hive.
- Check the hive comb surface until the presence of queen and of all life stages of brood – eggs, larvae, pupae – are verified. The queen should be labelled for easy detection.
- If queen is not found and/or no open brood is present, queen failure is assumed (after rechecking in 1 week). Queen presence can be presumed, if eggs are still present.
- Please be aware that the queen might stop laying eggs prior to swarming, in early winter and during extreme weather events.
- Record queen presence, brood presence and any replacement queens in the BEEP app.

References

Delaplane KS, Van Der Steen J, Guzman-Novoa E (2013). Standard methods for estimating strength parameters of *Apis mellifera* colonies. Journal of Apicultural Research 52: 1-12.

EFSA AHAW Panel (EFSA Panel on Animal Health and Welfare) (2016). Scientific opinion on assessing the health status of managed honeybee colonies (HEALTHY-B): a toolbox to facilitate harmonised data collection. EFSA Journal 14: 4578, 241 pp.

Human H, Brodschneider R, Dietemann V*, et al*. (2013). Miscellaneous standard methods for *Apis mellifera* research. Journal of Apicultural Research 52: 1-53.

Acknowledgments

The authors of this protocol thank the B-GOOD Tier 1 and Tier 2 partners for their constructive feedback during the project duration to optimize the content for high quality data collection in the project. The B-GOOD project received funding from the European Union’s Horizon 2020 research and innovation programme under grant agreement number 817622.

B-GOOD partners:

This protocol was developed for B-GOOD project. B-GOOD stands for Giving Beekeeping Guidance by computational-assisted Decision making. The B-GOOD project received funding from the European Union’s Horizon 2020 research and innovation programme under grant agreement number 817622.

Liebefeld method

For researchers

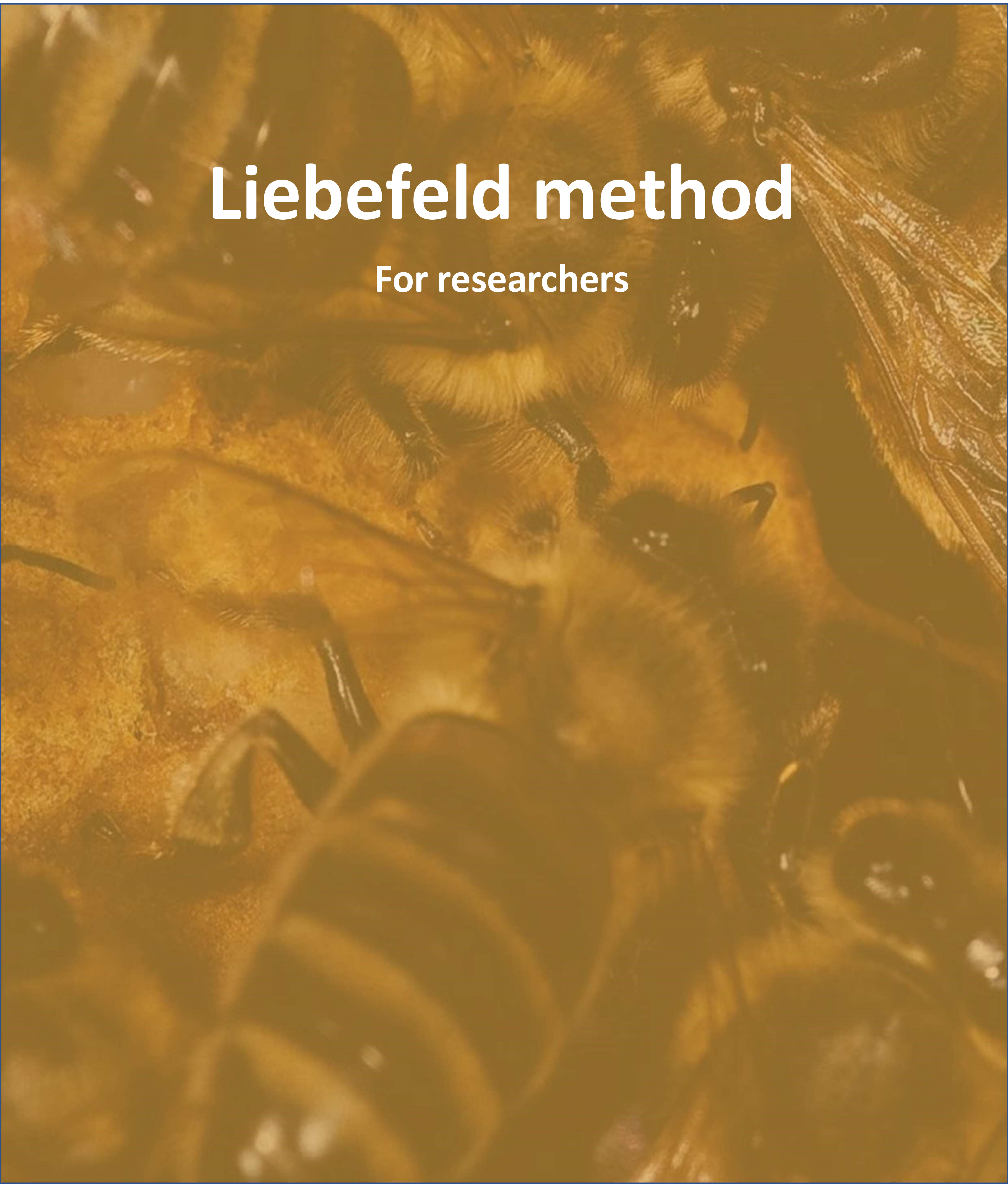

This protocol was developed for B-GOOD project. B-GOOD stands for Giving Beekeeping Guidance by computational-assisted Decision making. The B-GOOD project received funding from the European Union's Horizon 2020 research and innovation programme under grant agreement number 817622.

B-GOOD protocol title and code: Liebefeld method (P2)

Description: How to apply the Liebefeld method to estimate the amount of bees, brood and food resources

Version: 20220513

B-GOOD Tier: Tier 1

Target group: Researchers

Liebefeld method

Estimation of colony strength by assessing the amount of comb surface covered by bees/brood/honey/pollen with a grid.

The amount of food resources (honey and pollen), brood size and colony size are key determinants of colony development and survival. As an alternative to the digital photography and analysis method (P8), the Liebefeld method can be used to estimate colony traits. Preferably, the same person(s) should conduct the Liebefeld measurement to keep the experimenter bias variability at a minimum.

Measurements should be every 20 days during the bee season. The first measurement should start in spring, when the bees start to forage; the last measurement should be in autumn, before the over-wintering.

Materials

- Pre-marked grid

Making the pre-marked grid:
For convenience, we recommend to make an ‘open’ grid with metal wires by using a standard hive frame of the same dimensions with the frames used in the apiary (Figure 1).

- Drill small holes at 5 cm distance on the wooden frame on all four sides.
- Put wires through the holes and stretch them across the frame to connect holes and create squares.
- Measure the squares that are not 5x5 cm, so you know the surface area and are better able to estimate how much of a 5x5 cm square would be covered with bees, brood, etc.

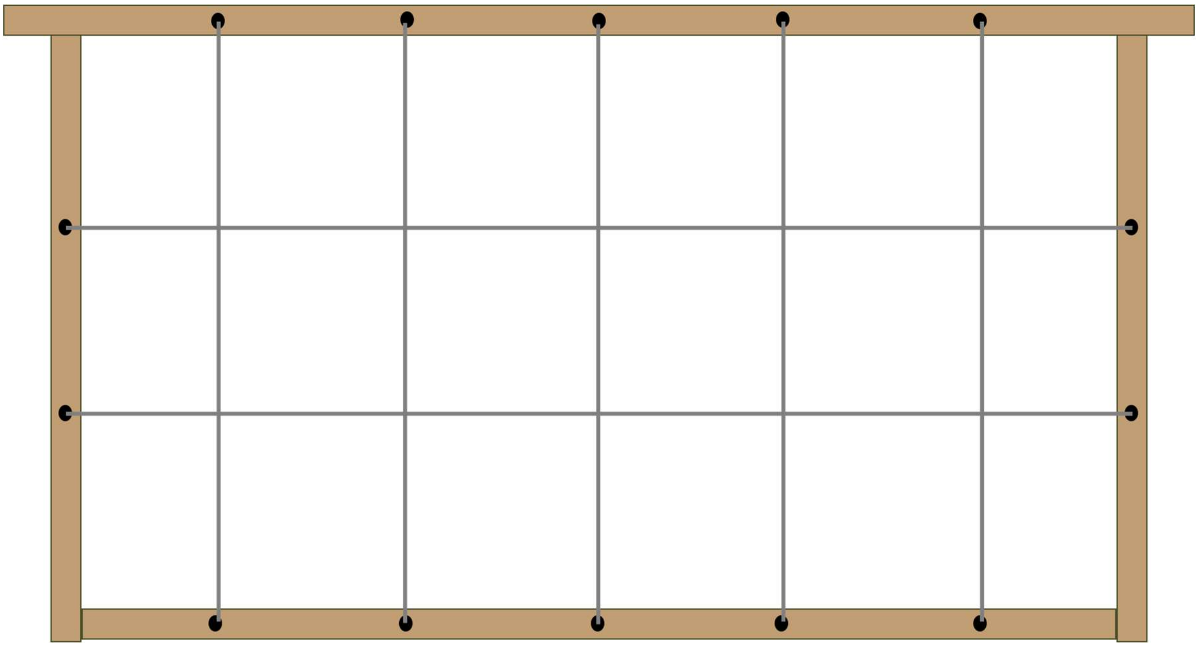

Figure 1: Scheme of grid for Liebefeld method. Squares should be 5x5 cm^2.

Field methods

- Colony traits that need to be measured are: colony size (bees), capped brood (pupae), open brood (larvae), eggs, drone brood, pollen stores, and honey (sealed only). When estimating, the separation between capped brood, open brood and eggs only applies to worker bees. When estimating drone brood, include brood in all stages (eggs, larvae and pupae).
- Open a colony and sequentially remove combs of bees (frames).
- Overlay each side of every comb in a hive with a grid pre-marked in 5x5 cm^2 (Figure 1).
    1. Measure the area covered with bees: count the total number of squares covered with bees per frame side. This includes the number of squares fully covered and the ones partially covered. The partially covered squares should be estimated as the proportion covered, up to one decimal point. Record the number of squares in the BEEP app for each side of all frames in the hive. The BEEP app will automatically calculate the sum of bees.
    2. Remove the bees from the frame and estimate the other parameters. For removing the bees, hold a frame above (or half in) the brood box and remove the bees by 1) moving the frame downwards with a sudden stop, 2) holding the frame by one 'ear' and tapping with your free hand on the hand holding the ear (a little rough on the eggs), or use a feather or soft brush (not very hygienic). Frames do not need to be free of bees completely. It is fine if bees stay on the frame as long as the brood and/or food reserves are visible and can be estimated. Repeat the counting of squares for all colony parameters and record the information in the BEEP app.
- In the BEEP app, take care to record the number of squares covered on both sides of each frame in the hive. If a parameter is not present, score it as 0. When entering the data, please keep the same sequence of frames for all parameters, such that in theory we could 'rebuild' the colony frame by frame.

- In case of honey super(s):
    - Colony traits that need to be measured in the supers are: bees and capped honey
    - The same procedure with brood box applies for estimation of these parameters in the honey super. The only difference is that, in the BEEP app instead of recording information per side of frame, record total amount of honey and/or bees for each super.

References

Delaplane KS, Van Der Steen J, Guzman-Novoa E (2013). Standard methods for estimating strength parameters of *Apis mellifera* colonies. Journal of Apicultural Research 52: 1-12.

EFSA AHAW Panel (EFSA Panel on Animal Health and Welfare) (2016). Scientific opinion on assessing the health status of managed honeybee colonies (HEALTHY-B): a toolbox to facilitate harmonised data collection. EFSA Journal 14: 4578, 241 pp.

Acknowledgments

The authors of this protocol thank the B-GOOD Tier 1 and Tier 2 partners for their constructive feedback during the project duration to optimize the content for high quality data collection in the project. The B-GOOD project received funding from the European Union's Horizon 2020 research and innovation programme under grant agreement number 817622.

B-GOOD partners:

This protocol was developed for B-GOOD project. B-GOOD stands for Giving Beekeeping Guidance by computational-assisted Decision making. The B-GOOD project received funding from the European Union's Horizon 2020 research and innovation programme under grant agreement number 817622.

B-GOOD

WAGENINGEN UNIVERSITY & RESEARCH

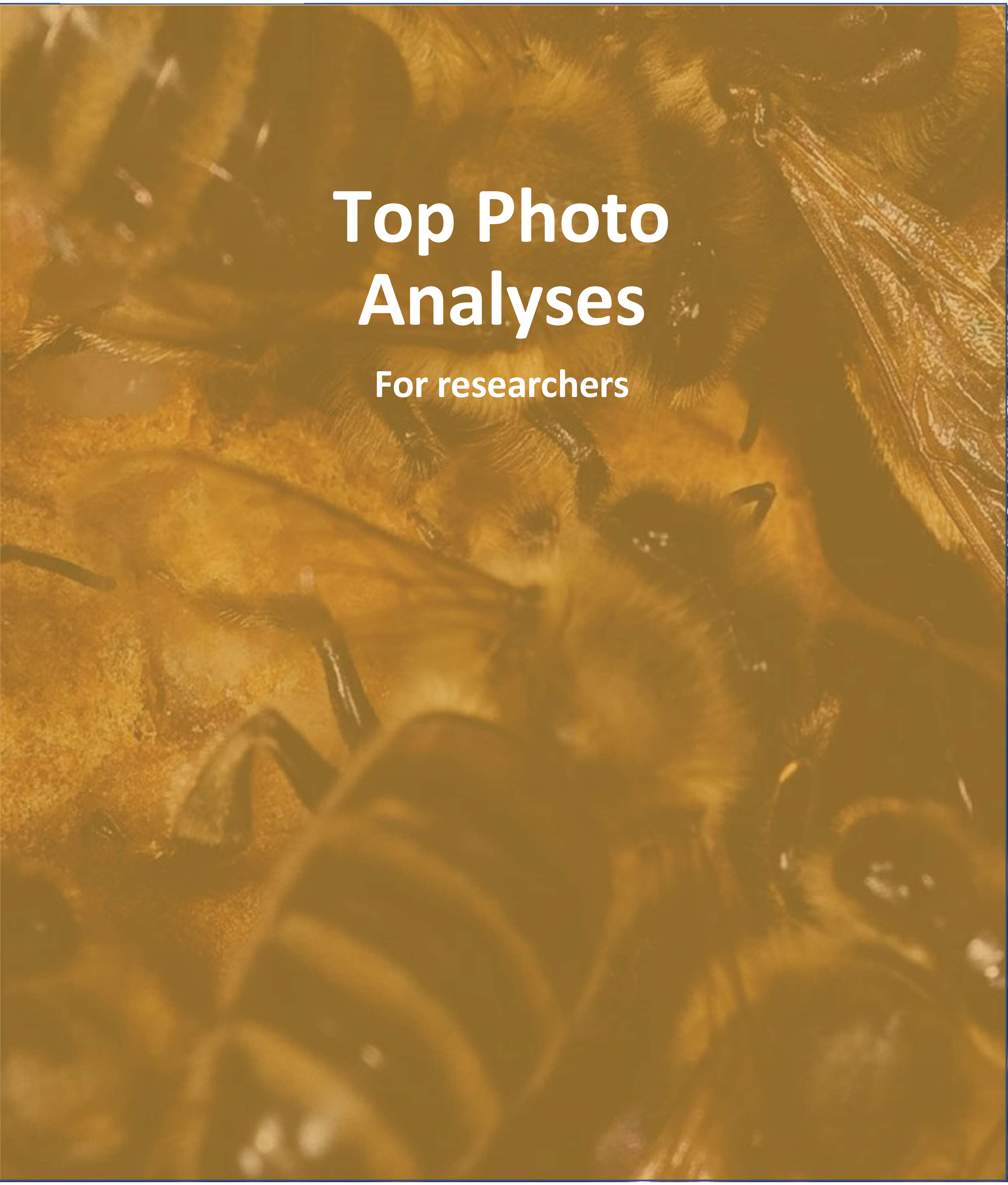

Top Photo Analyses

For researchers

This protocol was developed for B-GOOD project. B-GOOD stands for Giving Beekeeping Guidance by computational-assisted Decision making. The B-GOOD project received funding from the European Union's Horizon 2020 research and innovation programme under grant agreement number 817622.

B-GOOD protocol title and code: Top photo analyses (P3)

Description: Analyses of colony sizes by taking pictures of the broodbox from the top

Version: 20220513

B-GOOD Tier: Tier 1

Target group: Researchers

Top photo analysis

Colony size will also be estimated by taking a photo of the top-side of the hive. This will be done every first week of each month during winter, and every 20 days during the bee season. There are several benefits of this method over Protocol 2 and Protocol 8 as it is less invasive, faster, and easier, but particularly: (1) it can be used during winter when temperatures are too low for removing frames, (2) it may be easy enough for beekeepers to apply in Tier 2. However, it is a relatively rough and subjective estimation of the number of bees and should be interpreted accordingly.

This method was first published in: Van Dooremalen C, Cornelissen B, Poleij-Hok-Ahin C, *et al*.: **Single and interactive effects of *Varroa destructor*, *Nosema spp*., and imidacloprid on honey bee colonies (*Apis mellifera*)**. Ecosphere. 2018; 9(8): e02378.

Materials

- Camera – preferably DSLR
- Bee smoker

Field methods

- Blow a puff of smoke into the hive from below.
- After a minute, remove the lid and take first a high-resolution photo of your label/number/code of the hive and then of the top-side. For the accuracy of the photo analysis, take care to include the entire set of top frames in the photo, and use a standard angle lens (Figure 1).
- If there is more than one brood box:
    - Only take a photo of the top box and make sure that the number of boxes entered in the BEEP app is correct.
    - If you suspect that one of the brood boxes is empty of bees, then reduce and adjust the number accordingly in the app.

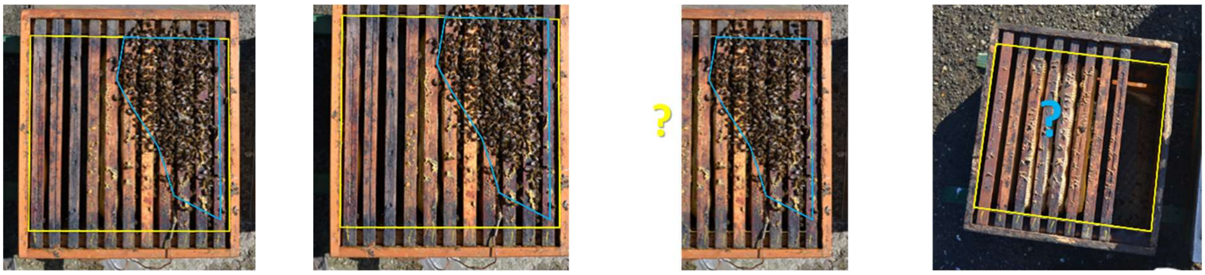

CORRECT | CORRECT | WRONG (does not include all the frames) | WRONG (bees are too deep and not visible)

Figure 1: Correct position of hive top in photograph and potential mistakes to avoid. Yellow lines show size of the top side of the box and blue lines show subjective estimation of top side of the bee cluster, if possible to estimate. Both lines are necessary to estimate the number of bees in the colony.

Computer Analysis

- Measure the available area and area occupied by bees using the software ImageJ. (https://imagej.nih.gov/ij/). Calculate the fraction covered in bees based on the number of pixels in a colony. To do so, calculate the ratio between the number of pixels of the area covered with bees (on top and visible between the frames) and the overall number of pixels of the top area of the box that represents the inner side of the box (see also yellow markings in Figure 1).
- Please see B-GOOD tutorial for a how-to video. (click here for tutorial)
- Enter the information of ‘pixels with bees’ and ‘pixels total top’ into the BEEP app.
- Upload the photo when entering data onto the BEEP app.

References

Van Dooremalen C, Cornelissen B, Poleij-Hok-Ahin C, *et al*. (2018). Single and interactive effects of *Varroa destructor*, *Nosema spp*., and imidacloprid on honey bee colonies (*Apis mellifera*). Ecosphere 9: e02378.

Van Dooremalen C, Van Langevelde F (2021). Can colony size of honeybees (*Apis mellifera*) be used as predictor for colony losses due to *Varroa destructor* during winter? Agriculture 11: 529.

Acknowledgments

The authors of this protocol thank the B-GOOD Tier 1 and Tier 2 partners for their constructive feedback during the project duration to optimize the content for high quality data collection in the project. The B-GOOD project received funding from the European Union's Horizon 2020 research and innovation programme under grant agreement number 817622.

B-GOOD partners:

This protocol was developed for B-GOOD project. B-GOOD stands for Giving Beekeeping Guidance by computational-assisted Decision making. The B-GOOD project received funding from the European Union's Horizon 2020 research and innovation programme under grant agreement number 817622.

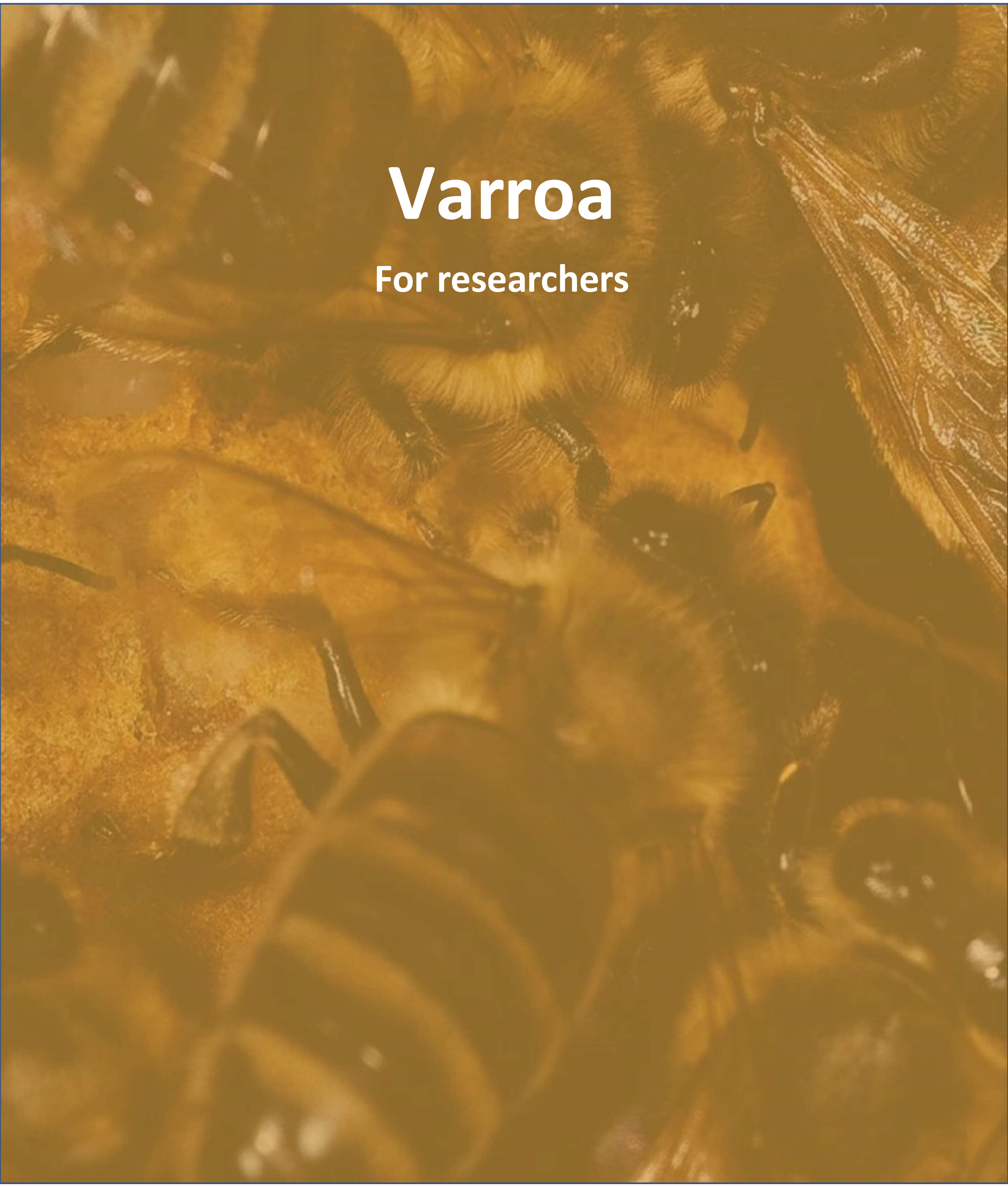

Varroa

For researchers

This protocol was developed for B-GOOD project. B-GOOD stands for Giving Beekeeping Guidance by computational-assisted Decision making. The B-GOOD project received funding from the European Union's Horizon 2020 research and innovation programme under grant agreement number 817622.

B-GOOD protocol title and code: Varroa (P4)

Description: Counting natural Varroa mitefall to measure mite infestation level

Version: 20220513

B-GOOD Tier: Tier 1

Target group: Researchers

Counting *Varroa* mites after (natural) fall

Mite infestation level of each colony will be estimated by quantifying naturally falling mites. The infestation levels will be counted preferably once a week, or at least once a month, throughout the beekeeping season. Although we apply standard *Varroa* control in the mini-apiaries and no Integrated Pest Management, it is still important to measure mite infestation levels of the hive as *Varroa* is considered to be one of the most harmful stressors for honey bees and treatments against it are not 100% effective.

Before, during and after Varroa treatments, we suggest to increase the number of measurements as such that they will give you a good indication of the effects of the mite treatment. In such cases, frequency of counting should depend on the treatment method, the duration of the treatment used, but potentially also the remoteness of the apiary.

When you apply varroa control methods, please record the date and treatment type in the BEEP app.

Materials

- Sticky board
- Guide for counting mites (Figure 1)

Field methods

- Use a screened bottom board for the hive, and place a sticky surface on the upper side of the board (the sticky surface should entirely cover the bottom of the hive).
- After one week, remove the sticky surface and count the number of mites. Place a guide above the board (Figure 1) to avoid counting the same mites. If dead bees are present on the board, check them as they act as magnets to fallen live mites.
- Divide the number of mites over the days that the sticky surface is left underneath the hive to obtain daily mite fall.
- If measurements will be continued, clean and replace the sticky board.

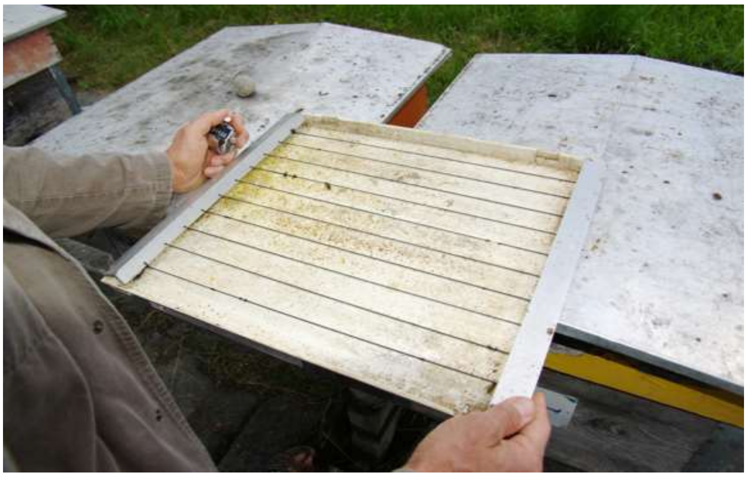

Figure 1: A guide placed on top of the *Varroa* sticky trap to help with mite counts (from Dietemann et al. 2013)

References

Delaplane KS, Van Der Steen J, Guzman-Novoa E (2013). Standard methods for estimating strength parameters of *Apis mellifera* colonies. Journal of Apicultural Research 52: 1-12.

EFSA AHAW Panel (EFSA Panel on Animal Health and Welfare) (2016). Scientific opinion on assessing the health status of managed honeybee colonies (HEALTHY-B): a toolbox to facilitate harmonised data collection. EFSA Journal 14: 4578, 241 pp.

OIE, World Organization for Animal Health (2021) Terrestrial Animal Health Code. Chapter 3.2.7. Varroosis of honey bees. (link) Accessed 15 July 2022

Acknowledgments

The authors of this protocol thank the B-GOOD Tier 1 and Tier 2 partners for their constructive feedback during the project duration to optimize the content for high quality data collection in the project. The B-GOOD project received funding from the European Union's Horizon 2020 research and innovation programme under grant agreement number 817622.

B-GOOD partners:

This protocol was developed for B-GOOD project. B-GOOD stands for Giving Beekeeping Guidance by computational-assisted Decision making. The B-GOOD project received funding from the European Union's Horizon 2020 research and innovation programme under grant agreement number 817622.

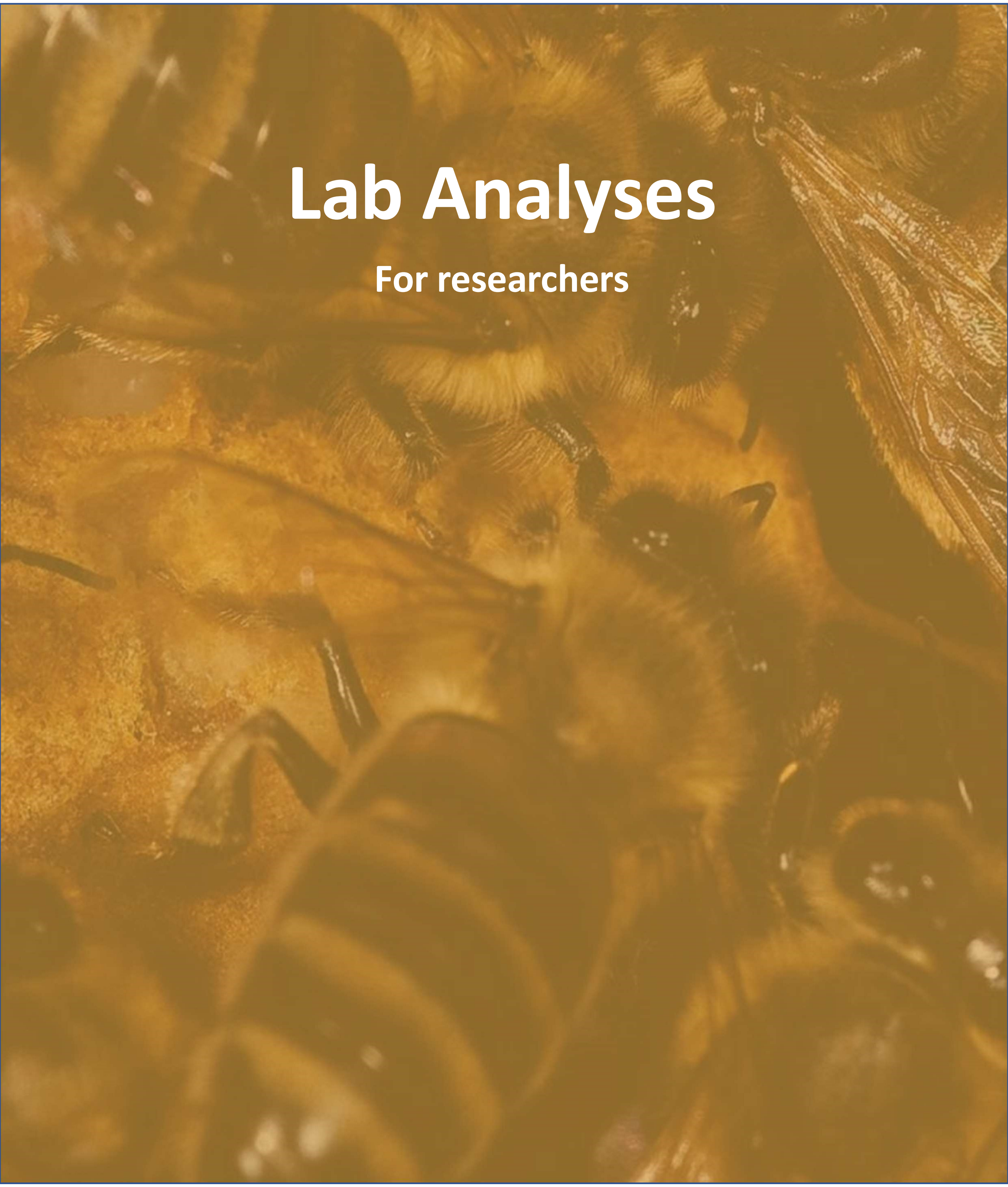

Lab Analyses

For researchers

This protocol was developed for B-GOOD project. B-GOOD stands for Giving Beekeeping Guidance by computational-assisted Decision making. The B-GOOD project received funding from the European Union's Horizon 2020 research and innovation programme under grant agreement number 817622.

B-GOOD protocol title and code: Lab analyses (P5)

Description: How to sample bees for lab analysis for diagnostic purposes

Version: 20220513

B-GOOD Tier: Tier 1

Target group: Researchers

Sampling bees for lab analyses

For diagnosis of bee diseases each colony will be sampled three times per year: 1) first in spring, when the bees start to forage; 2) second in summer, when the colonies have reached their maximum size; 3) and a third in autumn before the over-wintering.

BEEP app

- For anonymization purposes, the BEEP app will generate a unique identifier per sample for laboratory analysis. The samples should be labelled with these IDs. Please label the samples on the container and additionally place a label inside the container (on paper with a pencil), to freezer proof sample and ensure correct identification in case the outside label gets damaged or disappears during transport.
- Make sure the samples collected are correctly linked to the IDs to ensure correct feedback from the lab.
- Record the date of sample collection and the date that samples are sent to the reference labs in the BEEP app.
- Check that the date and time of the inspection containing the sample code in the BEEP app corresponds with the actual sample collection date and time.

Materials

- 8 x 100 ml cups
- 8 x perforated lids
- 8 x solid lid (no holes)

Collection of samples

- To collect samples, first open the colony and check the combs starting with the frames on the outer edge.
- Remove the first frame fully occupied by bees (most likely this will be a frame at the periphery of the brood nest).
- Make sure that the queen is not present on the comb; if present, return her to the hive (or take another frame).
- Fill a cup of 100ml with bees; it will be around 300 bees when the cup is full.
- One of the best methods to do this is to shake the bees from the frame onto a sheet, then bend or fold the sheet and use it to fill the bees into the cup. Many colonies have a covering

foil on top underneath the lid. One can use this sheet but you can also bring a sheet or use a sheet of newspaper. The cup should be placed on the top of the open colony so all bees that spill out of the cup fall directly back into the colony. With this method, you can easily fill the cup to the top.

- Alternatively, if the bees are calm, fill the cup by scraping the bees off the comb, holding the cup vertically and the comb at 45°.
- To keep bees alive until freezing, make sure that there are holes on the lid.
- It is fine if drones are included in the sample but less is better.
- Once in a lab, freeze bees as quickly as possible, by either using liquid nitrogen, dry ice or placing cups in a freezer at -80°C.
- After freezing, place a solid lid (no holes) on the cup to prepare samples for storage and transport.

Storage and transport

- As soon as the sample is frozen, it should never defrost until the moment of RNA or DNA extraction. Therefore, it is important to establish a continuous cold chain for the samples.
- The transfer of samples to the B-GOOD labs for lab analysis must be done on dry ice, by courier services that enable fast transport. Preferably, samples will arrive within 72h after sampling in the field at the B-GOOD labs. Arrival of samples should be within 48h from the moment that they are sent by courier.
- Apiaries should coordinate the sending date of samples with the B-GOOD labs before arranging transport. Make sure that samples do not arrive at the labs on weekends. Suggested planning:
  - Sampling on Monday/Tuesday
  - Send samples to the B-GOOD labs on Monday/Tuesday/Wednesday (latest)
- Samples should be sent together with the signed MTAs and a list of sample codes.

Dead colonies

A colony is considered dead if 1) the hive is absent of any living bees 2) the colony is too weak to recover in spring because (i) less than two frames are occupied by winter bees or (ii) the queen is dead and the hive cannot replace queen by building emergency cells (no brood).

- If colonies are dead, and there are still bees present in the hive, collect samples. Store them until the moment of regular sampling and send them to the reference labs with the same batch. Indicate on the sample that it involves a dead colony.
- If possible, collect and store the queen separately.
- Record in the BEEP app under ‘Loss’

References

Human H, Brodschneider R, Dietemann V*, et al*. (2013). Miscellaneous standard methods for *Apis mellifera* research. Journal of Apicultural Research 52: 1-53.

Matthijs S, Sciensano, BE-NRL Bee diseases. Pers. Comm. 2020

Schäfer MO, Friedrich-Loeffler-Institut, DE-NRL für Bienenkrankheiten. Pers. Comm. 2020

Van Dooremalen C, Wageningen Research. Pers. Comm. 2020

Acknowledgments

The authors of this protocol thank the B-GOOD Tier 1 and Tier 2 partners for their constructive feedback during the project duration to optimize the content for high quality data collection in the project. The B-GOOD project received funding from the European Union's Horizon 2020 research and innovation programme under grant agreement number 817622.

B-GOOD partners:

This protocol was developed for B-GOOD project. B-GOOD stands for Giving Beekeeping Guidance by computational-assisted Decision making. The B-GOOD project received funding from the European Union's Horizon 2020 research and innovation programme under grant agreement number 817622.

B-GOOD

WAGENINGEN UNIVERSITY & RESEARCH

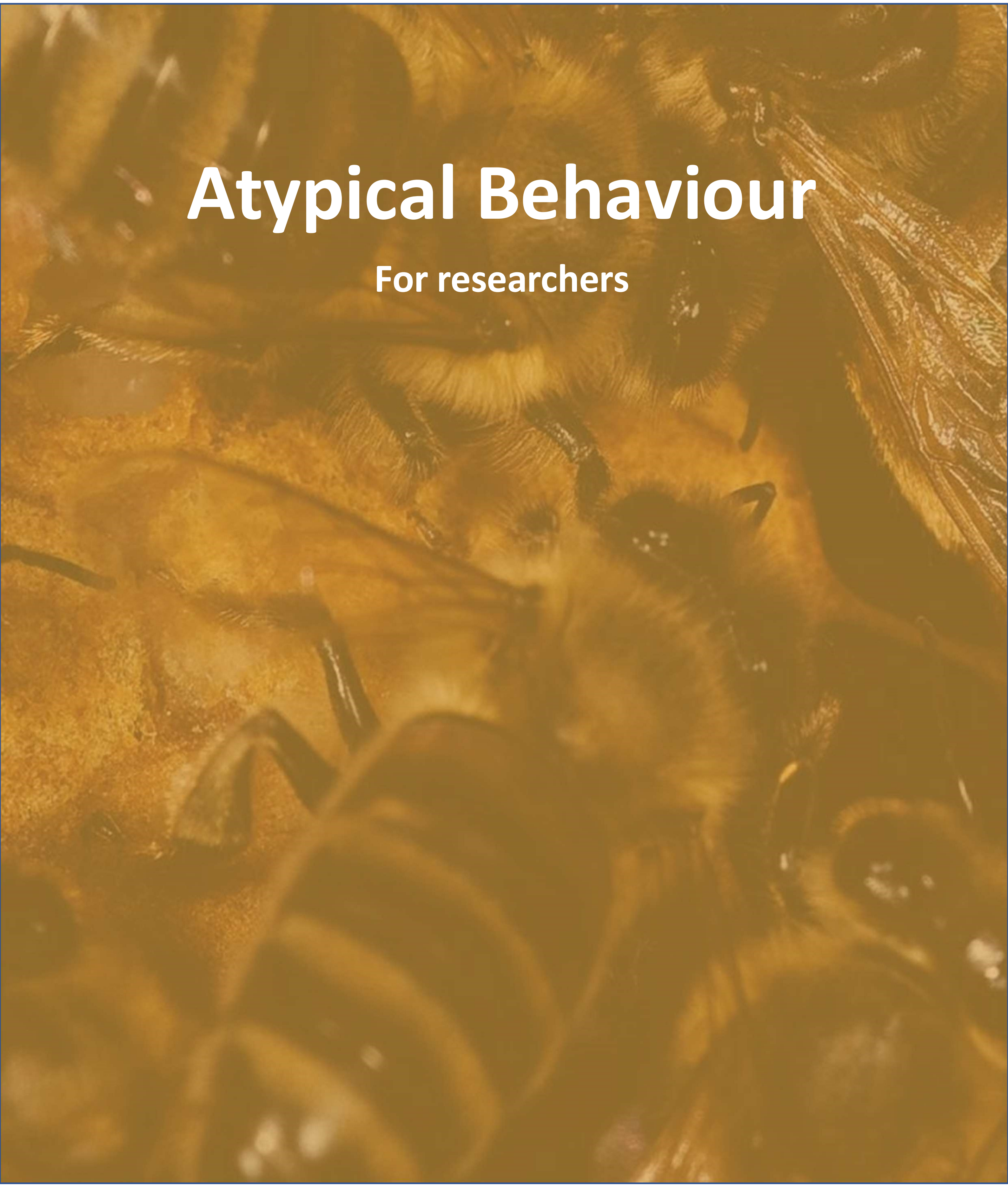

Atypical Behaviour

For researchers

This protocol was developed for B-GOOD project. B-GOOD stands for Giving Beekeeping Guidance by computational-assisted Decision making. The B-GOOD project received funding from the European Union's Horizon 2020 research and innovation programme under grant agreement number 817622.

B-GOOD protocol title and code: Atypical behaviour (P6)

Description: Visually assess colony behaviour

Version: 20220204

B-GOOD Tier: Tier 1

Target group: Researchers

Atypical worker behaviour

Atypical behaviour by workers is one of the first signals of diminished health within the colony. This indicator will be measured by visual inspection of the hive every 20 days during bee season. It assumes a basic level of normal typical behaviour of honeybees.

Field methods

- Visually assess several combs inside of the hive for the presence/absence of worker bees showing atypical behaviour.
- A majority (> 60%, including brood comb) of frames containing workers inside the hive should be inspected.
- Each frame side should be observed for 1 min for atypical behaviour.
- See Table 1 for honey bee behaviours; any behaviour outside of the normal repertoire of bees can be considered as atypical
- Some examples of atypical behaviours include: running quickly over the comb for long periods, trembling (aside from the trembling dance) or shaking.

Table 1: Honey bee worker behaviour catalogue (from Scheiner et al. 2013)

Task	Description
cell cleaning	removing debris from used brood cells (cocoons, larvae excretion), cleaning cell walls. Takes place in a cell not currently being used
general nest sanitation	removing debris from nest (mouldy pollen, old cappings, dead brood, and dead adults)
brood care	feeding larvae (head in brood cell > 1.3 min), attending queen
construction	smoothing wooden hive parts with mandibles and manipulating wax and propolis in cracks and corners of the hive
fanning wings	flapping wings while standing in hive/at entrance
food care	insertion of head into a cell containing nectar, receiving nectar-on bridge
grooming a nestmate	running nest mate body parts through mandibles
grooming self	running own body parts through mandibles
inspecting a cell	momentary insertion of the anterior portion of the head into an empty cell
nest care	manipulating wax of cells (not cappings), building new empty cells
patrolling	walking around nest
standing and chaining	standing stationary or hanging while stationary on nestmates
brood cap manipulation	trimming or smoothing wax cappings on brood cells and capping brood with wax
honey cap manipulation	trimming or smoothing wax cappings on cells of honey and capping honey with wax
trophallaxis	nestmate exchange of food (not near entrance), receiver thrusts tongue at donators mouthpart, donator opens mouthparts pushes tongue forward, and regurgitates a drop which is lapped up
vibrating	fast rhythmic body vibrations (non-dance)
head in pollen	insertion of head into a cell containing pollen
inspecting brood	head in brood cell, < 1.3 min
dancing	dancing without/with pollen
washboarding/ plaining	standing and rocking back and forth with mouthparts open
attending dance	dance attendance without/with pollen

References

EFSA AHAW Panel (EFSA Panel on Animal Health and Welfare) (2016). Scientific opinion on assessing the health status of managed honeybee colonies (HEALTHY-B): a toolbox to facilitate harmonised data collection. EFSA Journal 14: 4578, 241 pp.

Human H, Brodschneider R, Dietemann V, *et al*. (2013). Miscellaneous standard methods for *Apis mellifera* research. Journal of Apicultural Research 52: 1-53.

Scheiner R, Abramson CI, Brodschneider R, et al. (2013). Standard methods for behavioural studies of Apis mellifera. Journal of Apicultural Research 52: 1-58.

Acknowledgments

The authors of this protocol thank the B-GOOD Tier 1 and Tier 2 partners for their constructive feedback during the project duration to optimize the content for high quality data collection in the project. The B-GOOD project received funding from the European Union's Horizon 2020 research and innovation programme under grant agreement number 817622.

B-GOOD partners:

This protocol was developed for B-GOOD project. B-GOOD stands for Giving Beekeeping Guidance by computational-assisted Decision making. The B-GOOD project received funding from the European Union's Horizon 2020 research and innovation programme under grant agreement number 817622.

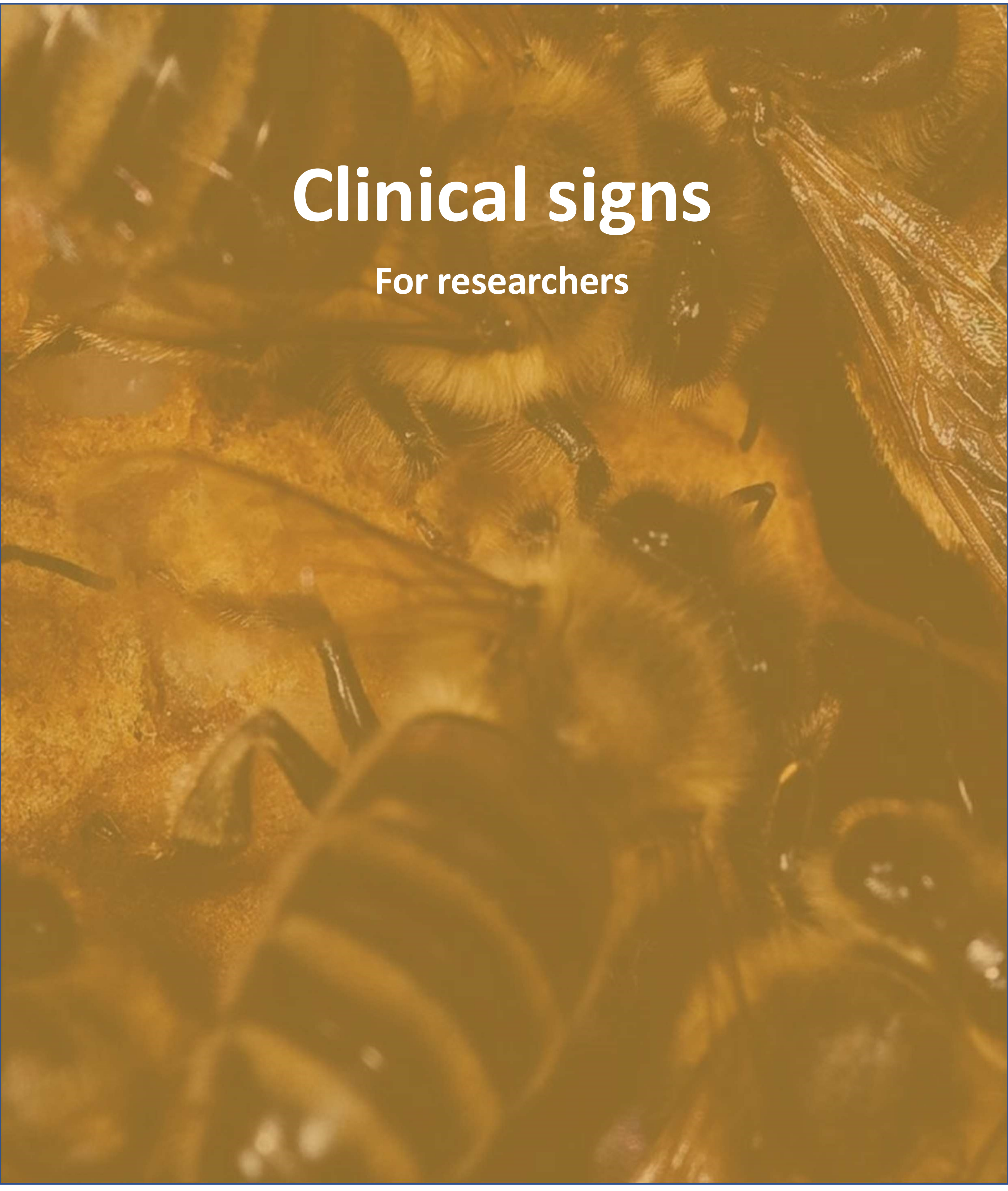

Clinical signs

For researchers

This protocol was developed for B-GOOD project. B-GOOD stands for Giving Beekeeping Guidance by computational-assisted Decision making. The B-GOOD project received funding from the European Union's Horizon 2020 research and innovation programme under grant agreement number 817622.

B-GOOD protocol title and code: Clinical signs (P7)

Description: How to visually check colonies for clinical signs of disease

Version: 20220204

B-GOOD Tier: Tier 1

Target group: Researchers

Clinical signs

During the beekeeping season, check the hive every 20 days for clinical signs of disease.

Field methods

- Visually observe colonies to assess the presence of clinical signs in brood and adult bees. A decision-making tree for clinical signs of diseases observed in European honey bee colonies can be found below (page 2 for brood and page 3 for adult bees). If you have the suspicion that a disease is present in the hive, record the type of disease in the BEEP app.

References

EFSA AHAW Panel (EFSA Panel on Animal Health and Welfare) (2016). Scientific opinion on assessing the health status of managed honeybee colonies (HEALTHY-B): a toolbox to facilitate harmonised data collection. EFSA Journal 14: 4578, 241 pp.

Kunneke A. Diagnoseboom. Imkerpedia. Published April 2022. Website: https://www.imkerpedia.nl/wiki/index.php/Bijenziekten_en_plagen

Kunneke A. Diagnoseboom. Newsletter Bees@wur, published April 2022. Website: http://mymeasuremail.com/880/Actions/Newsletter.aspx?historymessageid=30982

Diagnostic tree of honeybee and brood disease

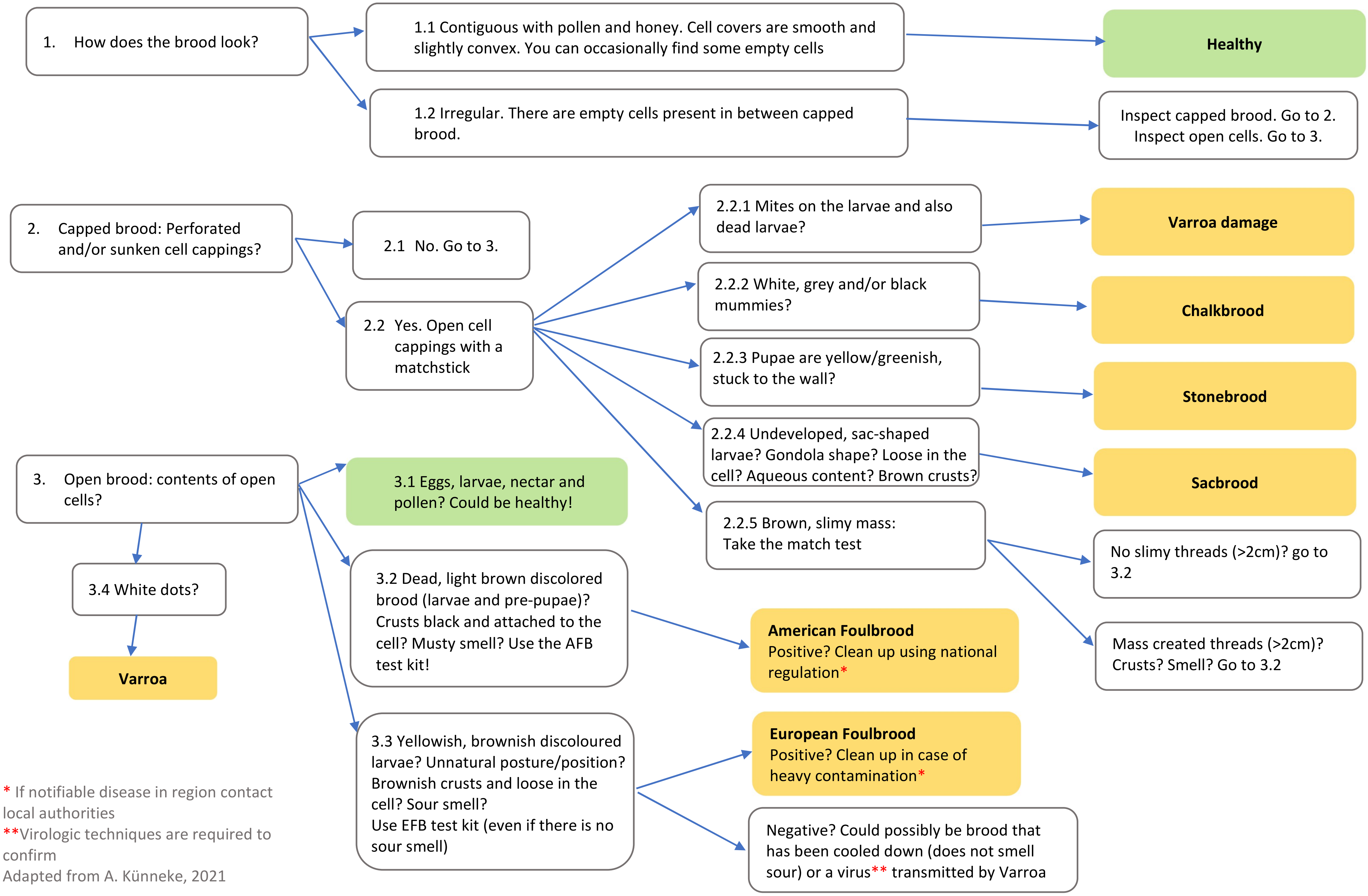

* If notifiable disease in region contact local authorities

**Virologic techniques are required to confirm

Adapted from A. Künneke, 2021

5. Bees with swollen abdomen?
- **Constipation** (May disease) paste like, yellow, brownish faeces. During spring. swollen abdomens (compared to healthy bees), paralysis, bees crawling on the outside of the hive. Low amount of dead bees. Apply pressure to the abdomen: yellow, solid paste. A swollen abdomen could also be a virus infection **

4. Bees with short abdomen?
- Varroa damage (could be in combination with a virus**) See 2.2.1 and 3.4

6. Low to high amount of black, hairless, shiny bees?
- Robbers or old, disembodied bees without hair. Black colour. Could be caused by certain forage sources.
- Trembling (sometimes black) bees on frames or at the entrance? Crawling bees? Low amount to many dead bees?
  - **ABPV, CBPV****

7. Deformed wings?
- Multiple possible causes: **DWV**** (see 2,3,5). **Poisoning** or **pesticides** (see 8). **Inbred. Hypothermic brood**

8. Many dead bees with protruding tongues (yes/no) in front and in the hive?
- Yes. Soaring, aggressive bees at the entrance and/or paralysis? Sudden increase of dead bees? Pupae with coloured eyes? In spring and summer.
  - Could be **poisoning** or **pesticides.** Contact national regulations service.
- No. Bees that are buried deep in frame cells?
  - Bees **starved** due to lack/shortage of food or due to robbery.

9. Hive empty? Colony gone?
- Are the contents of open cells empty? Check contents and go to 3.
- Did the colony swarm?
  - Colony could be empty due to **swarming.**

10. Damage/observations to/on the hive, frames and/or bottom board
- Fallen heads, abdomen or wings on the bottom board
  - **Mice**
- Bee excrement stains?
  - Multiple possible causes
    - **Amoeba**. Yellow faeces in winter and in spring. Swollen abdomen, crawlers. Lots of dead bees***
    - **Nosema**. With or without excrement stains (diarrhoea) on or in the hive. Droplet shaped. Swollen abdomen. Crawlers. Struggling colony. Especially in spring, but sometimes during winter. Lots of dead bees**
    - **Constipation**. Brown faeces on frames, hive and entrance. Diarrhoea from the winter bees. Agitation. Crawling bees. Low amount to lots of dead bees.***
    - **Viruses** could also cause diarrhoea.**
- Holes or webbing in the frames?
  - **Waxmoth larvae**
- Holes in the hive?
  - **Woodpecker**

** Virologic techniques are required to confirm
*** Microscopic inspection is needed to confirm
Adapted from A. Künneke, 2021

Acknowledgments

The authors of this protocol thank the B-GOOD Tier 1 and Tier 2 partners for their constructive feedback during the project duration to optimize the content for high quality data collection in the project. The B-GOOD project received funding from the European Union's Horizon 2020 research and innovation programme under grant agreement number 817622.

B-GOOD partners:

This protocol was developed for B-GOOD project. B-GOOD stands for Giving Beekeeping Guidance by computational-assisted Decision making. The B-GOOD project received funding from the European Union's Horizon 2020 research and innovation programme under grant agreement number 817622.

EFSA Protocol

For researchers

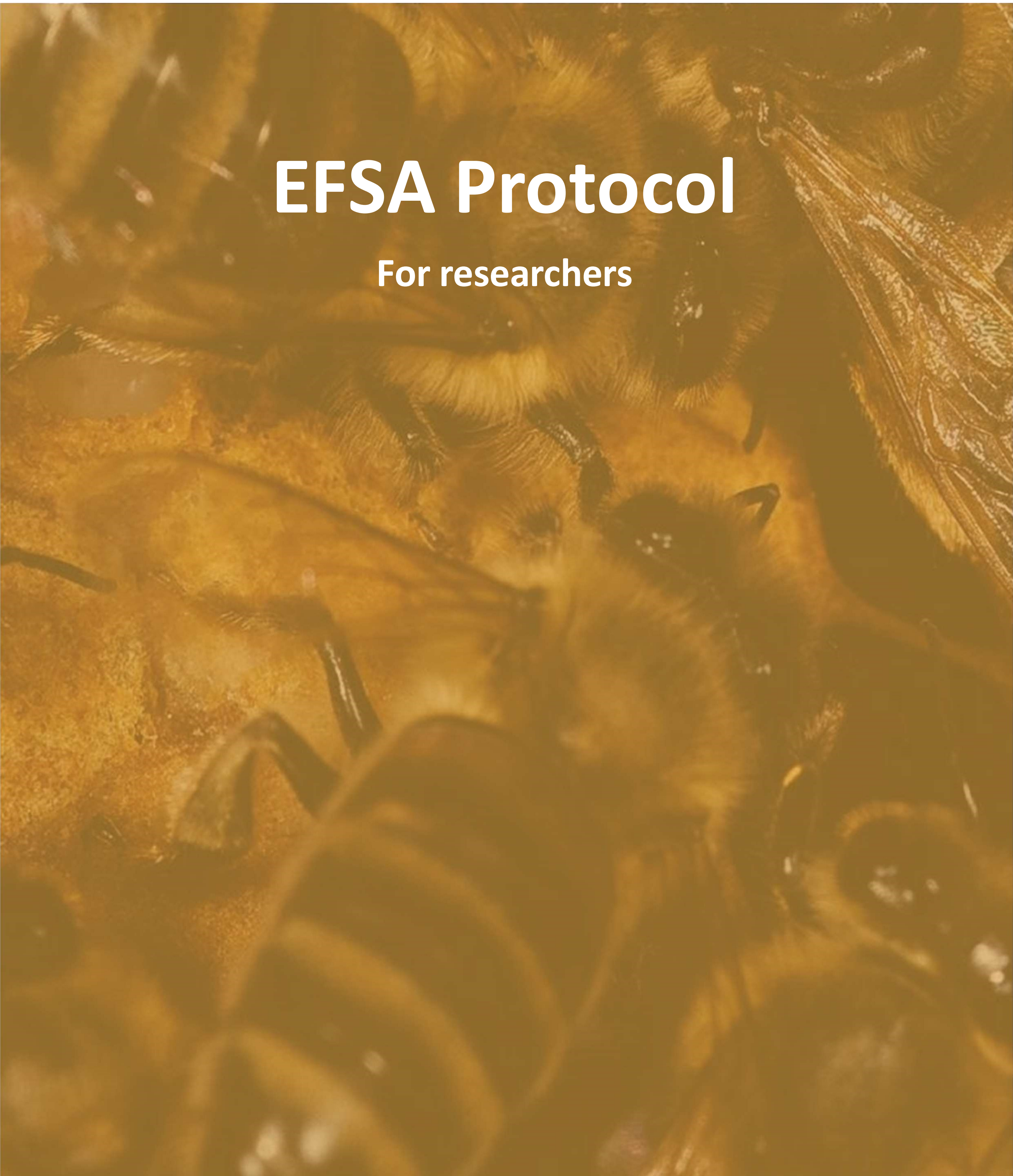

This protocol was developed for B-GOOD project. B-GOOD stands for Giving Beekeeping Guidance by computational-assisted Decision making. The B-GOOD project received funding from the European Union's Horizon 2020 research and innovation programme under grant agreement number 817622.

B-GOOD protocol title and code: EFSA protocol (P8)

Description: Performing the EFSA to estimate colony size, amount of brood and food resources

Version: 20220513

B-GOOD Tier: Tier 1

Target group: Researchers

Estimating colony size, resources and amount of brood (EFSA protocol)

This protocol was based on and validated within the study: Capela N, Dupon YL, Samento A, et al. **High accuracy monitoring of honey bee colony development by a quantitative method**. J Apicult Res. 2022 (accepted)

Objective

This field protocol describes the data collection used in the EFSA project (OC/EFSA/SCER/2017/02). The protocol was re-adapted for the B-GOOD mini-apiaries.

Materials

Experimental material

- Field forms
- Empty super
- Scale (max. weight 5kg, d=5g), e.g. a hook scale with a support (for a good working height)
- Extra batteries for scale
- Camera (for photographing combs)
- Tunnel + LED lights + support
- Battery for LEDs
- Electrical wire/cable drum
- Marker (for marking frames)

Beekeeping material

- Queen cage
- Queen marker
- Hive tool
- Bee brush
- Smoker
- Lighter
- Smoker fuel
- Beekeeping protective equipment

Experimental setup

All hives are marked with permanent numbers. Each super is numbered (bottom super =1), and each frame is marked (with a thick marker on the wood) on both sides (side A and B) and on top with a unique code. For instance, hive 1, super 2, frame 7 the code will be: PT.1.2.7.A

Timings and frequency of in hive monitoring

Each site will be visited once every 20 days. Monitoring should be done once every 20 days starting in early spring (data from hive scales can be used to decide when to start the monitoring). It is extremely important that data is collected within 21 days of the last visit, in order for the sampling frequency to cover the developmental time of workers (21 days from egg to adult). However, hive monitoring should be adapted to weather conditions (not possible when raining, and precautions should be taken when temperatures are low (<14 degrees).

Timing of observations may be important, because some foragers will be in the field. Sequence of hive monitoring and visits to study sites should be randomized.

Considerations before starting the in-hive monitoring

Precautions during cold conditions in spring:

- It is important that the brood and queen will not be cold, because they are very sensitive to cooling down, and in spring the colonies contain only few worker bees to generate heat. Hence, food combs should be photographed first, and bees on the brood combs should only be removed as the last task before closing the hive. Adult bees should be brushed directly into the original nest. Handling should be as fast as possible during the photographing of brood, in order for the colony not to lose heat.

Precautions during hot periods:

- It is important that the brood and queen will not be overheated. Hence, the caged queen should be kept in the shade.

Precautions during late summer:

- Opening the colonies for a prolonged period, particularly during late summer, may result in honey robbing. In order not to leave the experimental colonies vulnerable to honey robbing during the monitoring, the assessment should be done as early in the morning as possible.
- Another option could be closing the entrance of the colonies that are not being evaluated with a sponge.

1 Monitoring of experimental hives

1.1 Photographing of combs

1.1.1 Material installation

Prepare the material as follows:

- Install the camera in the tunnel and turn on the lights (set the LED lights into the maximum).
- Put the empty super next to the hive that will be measured
- Turn the scale on and tare it
- Wear latex gloves (like those that are used in the lab) on top of the thick gloves. Operators should change the latex gloves between hives. This prevents cross contamination between hives, if there is a diseased colony.

Important:

- Always check the scale tare between each weighing

1.1.2 Hive opening

Record the time the hive is handled (i.e. starting time and ending time), as this will affect the automatically collected hive scale data.

1.1.3 Frames weight with bees

Dispense smoke at the entrance before you start to keep bees calm and on the frame. Most likely, you would want to combine this analysis with Protocol P3 TPA ; if so, we suggest to first take a picture from the top.

Start with top super
Take out the frames **one by one** from the hive.

- First grab **frame 1**
- Weigh the frame with bees (record the weight of the frame **with bees**)
- Rest it **softly** in the empty super (without dropping the bees)
- Afterwards, take out **frame 2**
- Proceed the same way as in frame 1 (as well as for all the remaining frames from **frame 2 to frame 10**)
- if bees are left in the super, also weigh the super with and without bees

Start measuring from the top box and repeat the process until you weigh all the boxes from that colony. This way, your **bottom box** will be on the **top** when you finish this first task.

Important:

- Always start from the frame at your **left (frame 1)** and finish with the **rightmost (frame 10)** knowing that right and left are defined by the operator's position, who will be positioned behind the hive (**see Figure 1**).
  It is essential to put the frames in the empty super in exactly the **same order and direction** as in the hive. To do this, put a mark on every frame always on the same side.

- Encage the queen as soon as possible to protect it from potential clumsiness (and mark it if necessary).
- It is important to know the weight of "frame 5" with bees and without them. So, if we exchange the position of the frames we need to be careful writing the data because "frame 5" could be in the position of the 7th position.

Figure 1: Hive's scheme

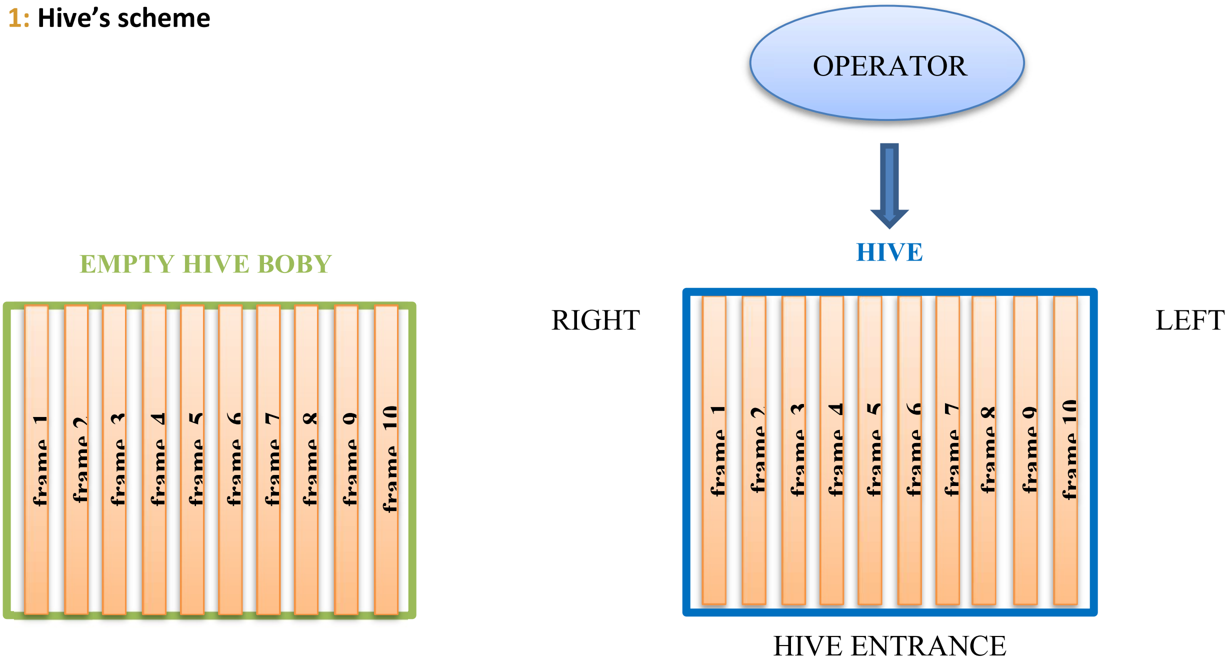

All frames should be labelled properly before you start taking photos

1.1.4 Frames weight 'without bees' and brood measurements

Once the hive is empty, weigh the remaining bees on the hive bottom if possible. If not possible, make an estimation on the number of bees considering that a full side of the box is equivalent to approximately 1000 bees.

Afterwards, grab **frame 10** (from the 1st box – it is the **original bottom box**):

- Brush the bees into the beehive in order to remove all the bees
- Weigh the frame (record the frame weight **without bees**)
- Take two pictures using remote control of the camera from both sides for honey, beebread, capped brood, larvae and eggs analysis. **Check if the first photos are in focus and capturing the whole frame!**
- Put it back in the hive in the initial location and side
- Grab **frame 9** and proceed the same way as in the frame 10 (as well as for all the remaining frames from **frame 9 to frame 1**)
- Photos should be checked in the end (after the hive is closed) if in focus. If the tunnel moves, the focus should be checked again.

1.2 End of operations

When all the frames are placed identically in the hive:

- release the queen in the hive
- close the hive while taking care not to crush the queen
- register the ending time of operations for each hive
- all field sheets should be kept in a folder
- check photos to see if they are out of focus

When back in lab:

- Transfer image files to computer. Organizing image files, it is important to keep track of date, locality, hive number and frame number.
- Data from field data sheets are entered and stored in a platform.

References

Alves TS, Pinto MA, Ventura P, *et al*. (2020). Automatic detection and classification of honey bee comb cells using deep learning. Computers and Electronics in Agriculture 170: 105244.

Capela N, Dupon YL, Samento A, *et al*. (2022) High accuracy monitoring of honey bee colony development by a quantitative method. Journal of Apicultural Research (accepted)

Delaplane KS, Van Der Steen J, Guzman-Novoa E (2013). Standard methods for estimating strength parameters of Apis mellifera colonies. Journal of Apicultural Research 52: 1-12.

EFSA AHAW Panel (EFSA Panel on Animal Health and Welfare) (2016). Scientific opinion on assessing the health status of managed honeybee colonies (HEALTHY-B): a toolbox to facilitate harmonised data collection. EFSA Journal 14: 4578, 241 pp.

Acknowledgments

The authors of this protocol thank the B-GOOD Tier 1 and Tier 2 partners for their constructive feedback during the project duration to optimize the content for high quality data collection in the project. The B-GOOD project received funding from the European Union's Horizon 2020 research and innovation programme under grant agreement number 817622.

B-GOOD partners:

This protocol was developed for B-GOOD project. B-GOOD stands for Giving Beekeeping Guidance by computational-assisted Decision making. The B-GOOD project received funding from the European Union's Horizon 2020 research and innovation programme under grant agreement number 817622.

Drone eggs

For researchers

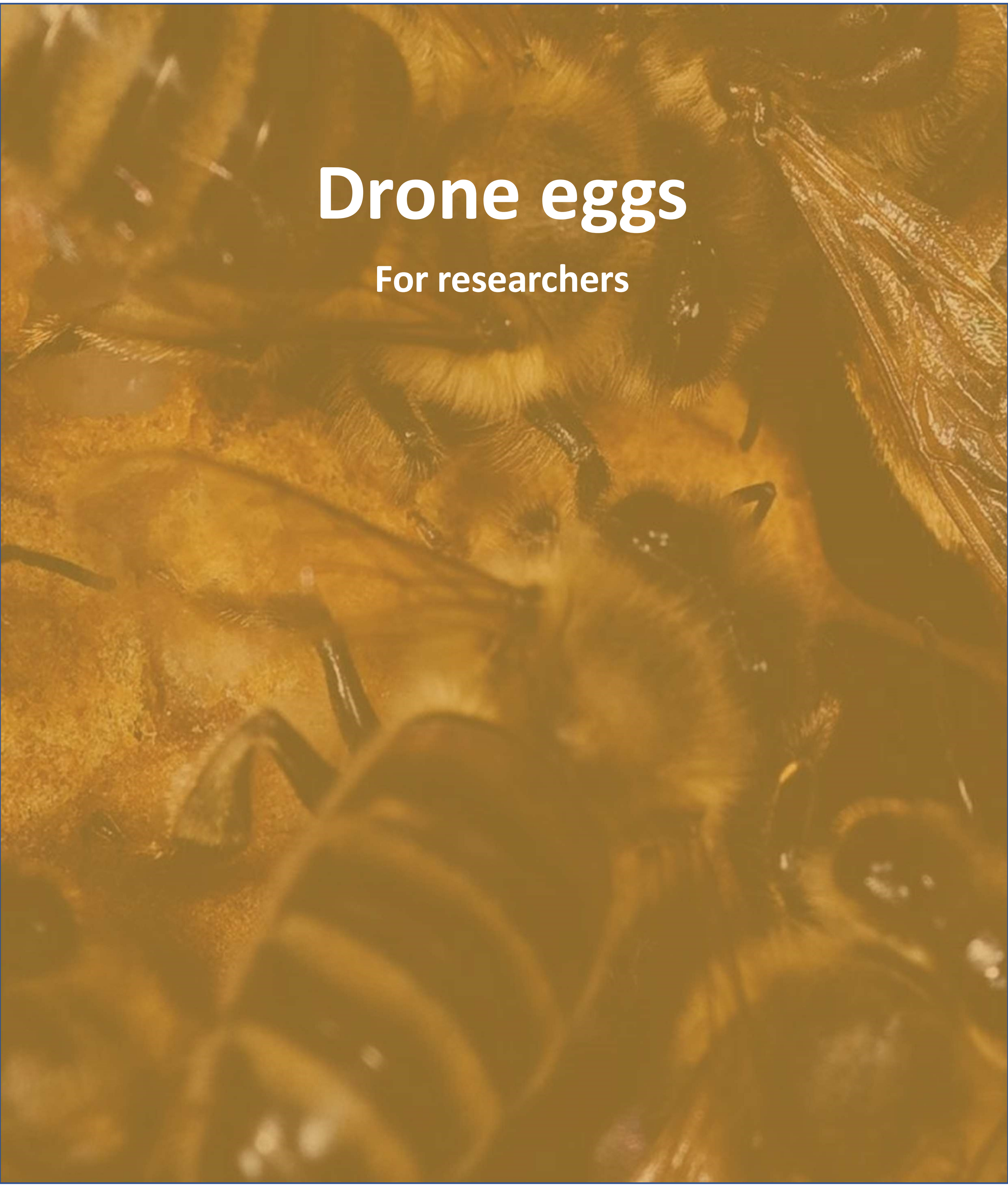

This protocol was developed for B-GOOD project. B-GOOD stands for Giving Beekeeping Guidance by computational-assisted Decision making. The B-GOOD project received funding from the European Union's Horizon 2020 research and innovation programme under grant agreement number 817622.

B-GOOD protocol title and code: Drone eggs (P9)

Description: How to collect drone eggs for lab analyses

Version: 20220513

B-GOOD Tier: Tier 1

Target group: Researchers

Sampling drone brood eggs

This protocol related to the previous work: De Graaf DC, Laget D, De Smet L, *et al*. **Heritability estimates of the novel trait 'suppressed in ovo virus infection' in honey bees (*Apis mellifera*).** Sci Rep. 2020; 10, 14310.

Samples will be collected once a year, combined with the spring visit for sampling bees for lab analyses. If drone brood is not present in some colonies during the spring, these colonies should be sampled the next visit that drones are present. In case the queen is replaced, new samples should be collected from the moment that drone eggs are present.

Materials

- 8 toothpicks
- 8 Eppendorf tubes with screw cap
- Fridge with cold packs or freezer

Field methods

- Open a hive and search for freshly laid drone eggs.
- Unscrew the Eppendorf tube and keep it ready.
- To collect the eggs, scoop the toothpick under the egg and lift it up (Figure 1.1). If it is difficult to see the eggs, cutting out a part of the wax can help (Figure 1.2).
- Take the Eppendorf tube and place the eggs approximately 1 cm deep in the tube (Figure 1.3 and 1.4).
- Place the egg flat against the tube and turn the toothpick a quarter stroke to release the egg from the toothpick (Figure 1.5 and 1.6).
- When inserting the toothpick, pay attention not to damage previously collected eggs.
- Repeat these steps until 10 drone eggs are collected and firmly close the Eppendorf tube.
- Write the unique ID of the hive on a sticker taped on the Eppendorf tube. This unique ID is the same ID given to the bee samples collected from each hive for disease diagnostics. If drone eggs are sampled at a later date than disease analyses sampling, please use the last code generated by the BEEP app for disease analysis. Make sure you clearly state the sampling date on the samples, in addition to the code

- Collect the required 10 drone eggs for each hive in the mini-apiary. Use a new toothpick and Eppendorf tube for each hive.
- Place samples in a -20 freezer as quickly as possible. For remote apiaries samples can be transported in a fridge with cold packs but should be placed in the freezer as soon as possible.
- Samples should be sent to the reference labs along with the bee samples for disease analysis.

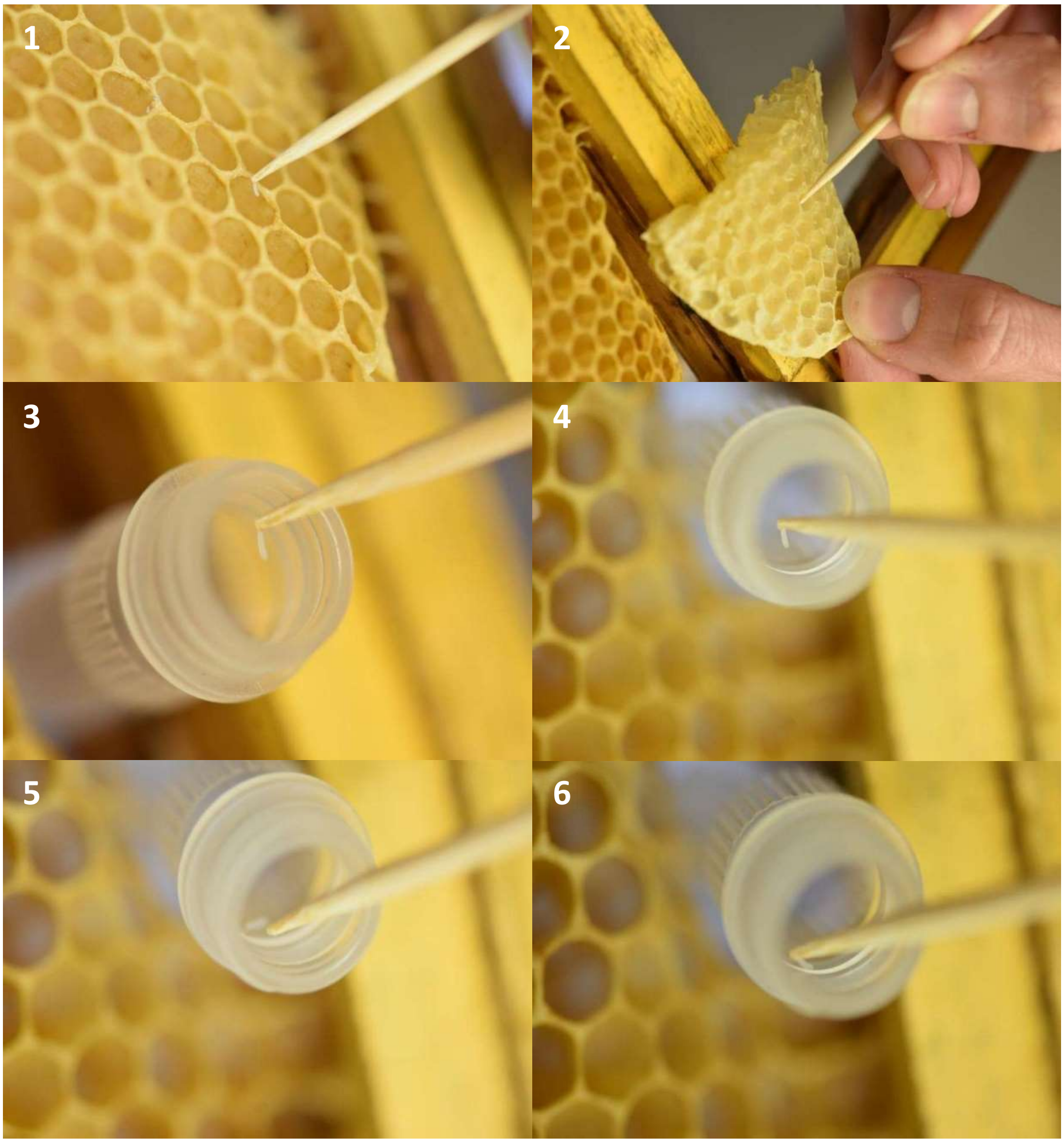

Figure 1 :Sampling drone eggs from a frame, in six steps (images 1-6).

References

De Graaf DC, Laget D, De Smet L, *et al*. (2020). Heritability estimates of the novel trait 'suppressed in ovo virus infection' in honey bees (*Apis mellifera*). Scientific Report 10: 14310.

Acknowledgments

The authors of this protocol thank the B-GOOD Tier 1 and Tier 2 partners for their constructive feedback during the project duration to optimize the content for high quality data collection in the project. The B-GOOD project received funding from the European Union's Horizon 2020 research and innovation programme under grant agreement number 817622.

B-GOOD partners:

PENSOFT
INRA SCIENCE & IMPACT
NOTTINGHAM TRENT UNIVERSITY
USAMV 1869
UNIVERSITEIT GENT
WAGENINGEN UNIVERSITY & RESEARCH
FRIEDRICH-LOEFFLER-INSTITUT FLI Bundesforschungsinstitut für Tiergesundheit Federal Research Institute for Animal Health
UNIVERSITÄT BERN
BEESOURCES BEEKEEPING CONSULTANCY
AARHUS UNIVERSITY
UNIVERSIDADE D COIMBRA
sciensano
SML Suomen Mehiläishoitajain Liitto
BEEP
JAGIELLONIAN UNIVERSITY IN KRAKÓW
SCIPROM Science, Management & Communication

This protocol was developed for B-GOOD project. B-GOOD stands for Giving Beekeeping Guidance by computational-assisted Decision making. The B-GOOD project received funding from the European Union's Horizon 2020 research and innovation programme under grant agreement number 817622.

B-GOOD

WAGENINGEN
UNIVERSITY & RESEARCH

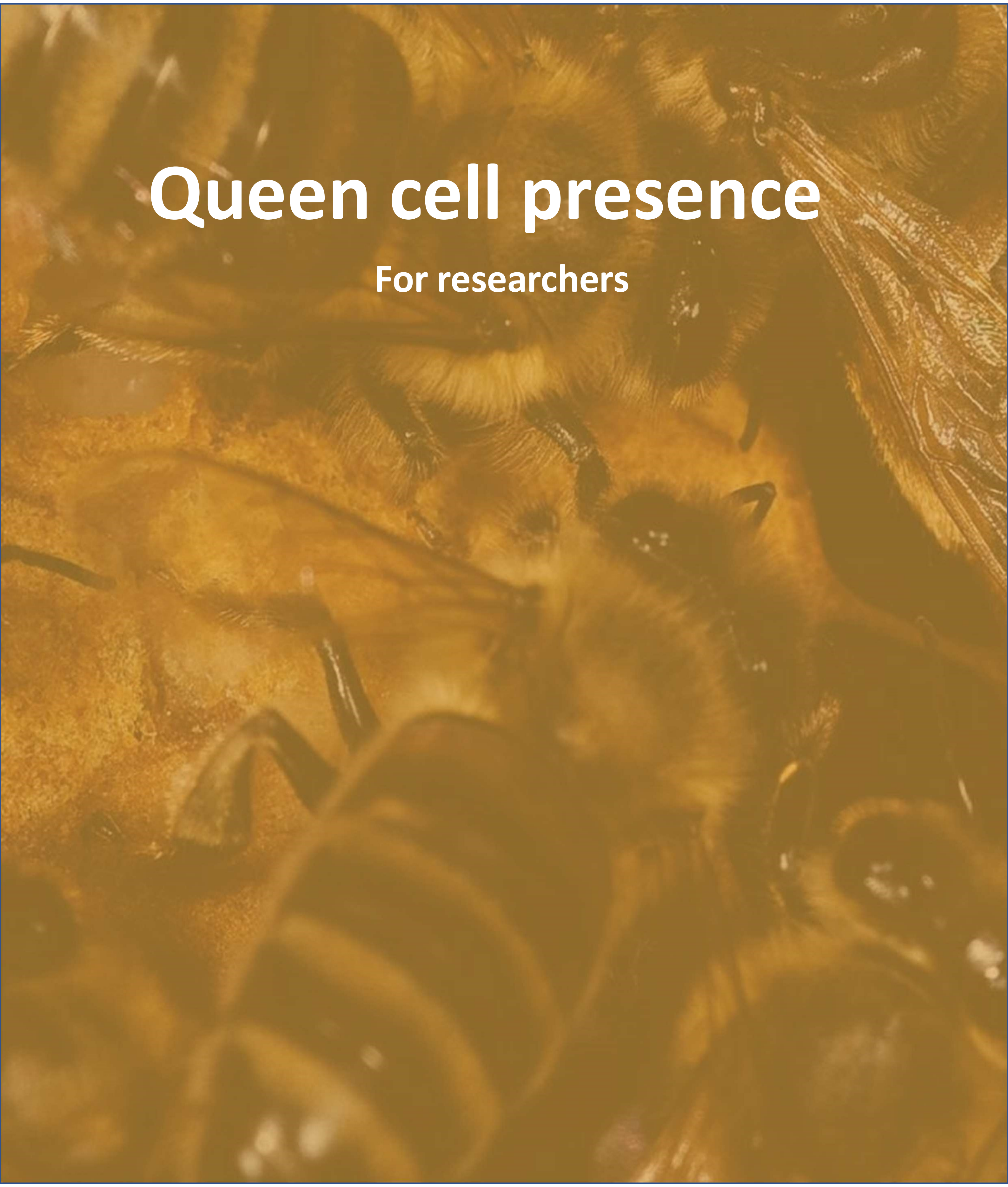

Queen cell presence

For researchers

This protocol was developed for B-GOOD project. B-GOOD stands for Giving Beekeeping Guidance by computational-assisted Decision making. The B-GOOD project received funding from the European Union's Horizon 2020 research and innovation programme under grant agreement number 817622.

B-GOOD protocol title and code: Queen cell presence (P10)

Description: Checking the colony for queen cells and explaining the four different queen cell types

Version: 20220204

B-GOOD Tier: Tier 1

Target group: Researchers

Queen cell presence

Every 20 days during the beekeeping season, check the colonies for presence of queen cells.

Field methods

- Open a colony and sequentially remove frames from the hive.
- Shake or brush every brood comb free of bees and examine carefully for presence of queen cells.
- Record the presence and type of queen cell in BEEP app (queen cup, emergency cell, swarm cell, supersedure cell).
- If queen cells are removed, please include this information as a management action.

Queen cell types:

- Queen cup: It is a small cup, with an opening on the bottom (Figure 1.A). For the purposes of this experiment, we define queen cups as empty queen cells (without eggs or larvae)
- Swarm cells: Are built when the colony is preparing to reproduce and swarm. These cells are usually present on the edges of the comb (Figure 1.B).
- Supersedure cells: Are built when the colony wants to replace the current queen. These cells are generally found on the centre of the comb (Figure 1.C.), but are not created from modified worker cells. Commonly the colony raises 1-3 supersedure cells.
- Emergency cell: Are built if the old queen is dead. Like supersedure cells, they are usually found on the centre of the comb (Figure 1.D), but these cells are created from modified worker cells. Usually, the colony raises a high number of emergency cells (especially compared to supersedure cells).

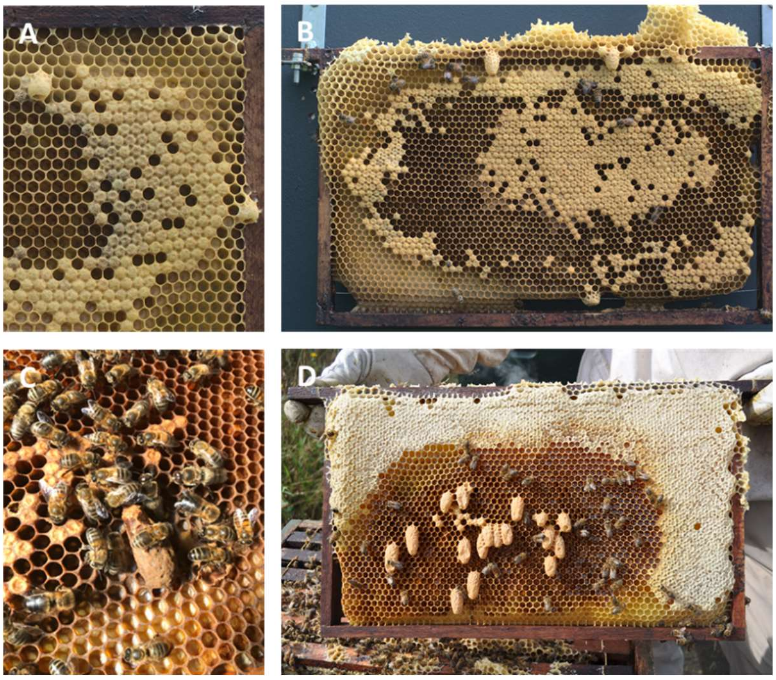

Figure 1*:* Different types of queen cells in honeybee colonies. A) Queen cups; B) Swarm cells; C) Supersedure cells if the queen is alive or emergency cells if the queen is dead; D) Emergency cells. Photo credits: C.van Dooremalen (A,B,D), M. Schoonman (C).

References

Delaplane KS, Van Der Steen J, Guzman-Novoa E (2013). Standard methods for estimating strength parameters of *Apis mellifera* colonies. Journal of Apicultural Research 52: 1-12.

EFSA AHAW Panel (EFSA Panel on Animal Health and Welfare) (2016). Scientific opinion on assessing the health status of managed honeybee colonies (HEALTHY-B): a toolbox to facilitate harmonised data collection. EFSA Journal 14: 4578, 241 pp.

Human H, Brodschneider R, Dietemann V*, et al*. (2013). Miscellaneous standard methods for *Apis mellifera* research. Journal of Apicultural Research 52: 1-53.

.

Acknowledgments

The authors of this protocol thank the B-GOOD Tier 1 and Tier 2 partners for their constructive feedback during the project duration to optimize the content for high quality data collection in the project. The B-GOOD project received funding from the European Union's Horizon 2020 research and innovation programme under grant agreement number 817622.

B-GOOD partners:

This protocol was developed for B-GOOD project. B-GOOD stands for Giving Beekeeping Guidance by computational-assisted Decision making. The B-GOOD project received funding from the European Union's Horizon 2020 research and innovation programme under grant agreement number 817622.

B-GOOD

WAGENINGEN UNIVERSITY & RESEARCH

Brood Pattern

For researchers

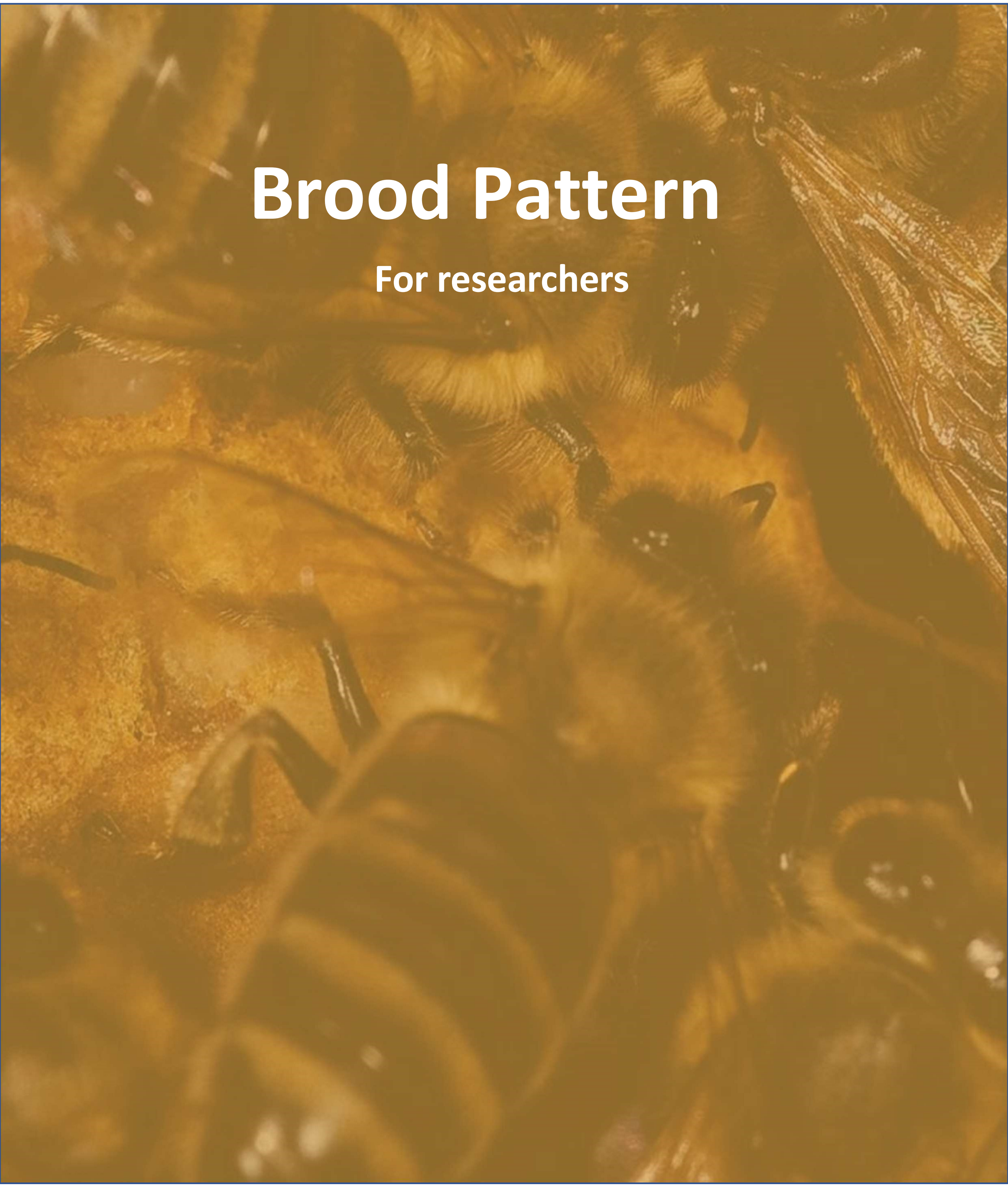

This protocol was developed for B-GOOD project. B-GOOD stands for Giving Beekeeping Guidance by computational-assisted Decision making. The B-GOOD project received funding from the European Union's Horizon 2020 research and innovation programme under grant agreement number 817622.

B-GOOD protocol title and code: Brood pattern (P11)

Description: How to measure brood pattern consistency

Version: 20220204

B-GOOD Tier: Tier 1

Target group: Researchers

Brood pattern consistency

This measurement should be done every 20 days during the beekeeping season. Brood pattern consistency is included as it is rated HH-H in the Healthy-B toolbox, and can provide support for data obtained through other measurements in acquiring a more complete view of the health status of a colony. Currently, we opted for this measurement for a qualitative or semi-quantitative assessment. Applying an accurate quantitative method is very labour intensive. Additionally, photo analysis of brood (P8) will already provide in-depth information on brood status.

Field method

- Open a colony and sequentially remove frames from the hive.
- Check the brood pattern consistency on each frame with brood.
- Estimate by eyesight the average percentage of empty brood cells within the area of the sealed worker and drone brood, compared to the total area of sealed brood cells in the colony.

[Alternative method (for people that prefer a more standardized, but labour-intensive method)

- Overlay the 'Liebefeld grid' on the surface of the frame with brood.
- Select 4 adjacent squares from the Liebefeld grid, thereby creating a 10x10 grid, that encompasses the area on the comb covered by the brood. This area will cover about 100 cells.
- Calculate the percentage brood consistency (100 – estimated empty cells).
- Repeat this procedure 10 times on different patches of brood.
- Calculate the average percentage of brood consistency

BEEP app

- Rate the overall brood pattern consistency based on a 5-point scale in the BEEP app

Rating information:

- 5: for areas with sealed brood with <5% empty cells.
- 4: for 5-10% empty cells,
- 3: for 10-20% empty cells,
- 1 or 2: 20% or more.

- Deduct points for spotty coverage of brood cells like larvae mixed with capped cells, lack of brood cluster across frames with largest brood at the centre of cluster, colour differences between caps, sunken caps.

References

Delaplane KS, Van Der Steen J, Guzman-Novoa E (2013). Standard methods for estimating strength parameters of *Apis mellifera* colonies. Journal of Apicultural Research 52: 1-12.

EFSA AHAW Panel (EFSA Panel on Animal Health and Welfare) (2016). Scientific opinion on assessing the health status of managed honeybee colonies (HEALTHY-B): a toolbox to facilitate harmonised data collection. EFSA Journal 14: 4578, 241 pp.

.

Acknowledgments

The authors of this protocol thank the B-GOOD Tier 1 and Tier 2 partners for their constructive feedback during the project duration to optimize the content for high quality data collection in the project. The B-GOOD project received funding from the European Union’s Horizon 2020 research and innovation programme under grant agreement number 817622.

B-GOOD partners:

This protocol was developed for B-GOOD project. B-GOOD stands for Giving Beekeeping Guidance by computational-assisted Decision making. The B-GOOD project received funding from the European Union’s Horizon 2020 research and innovation programme under grant agreement number 817622.

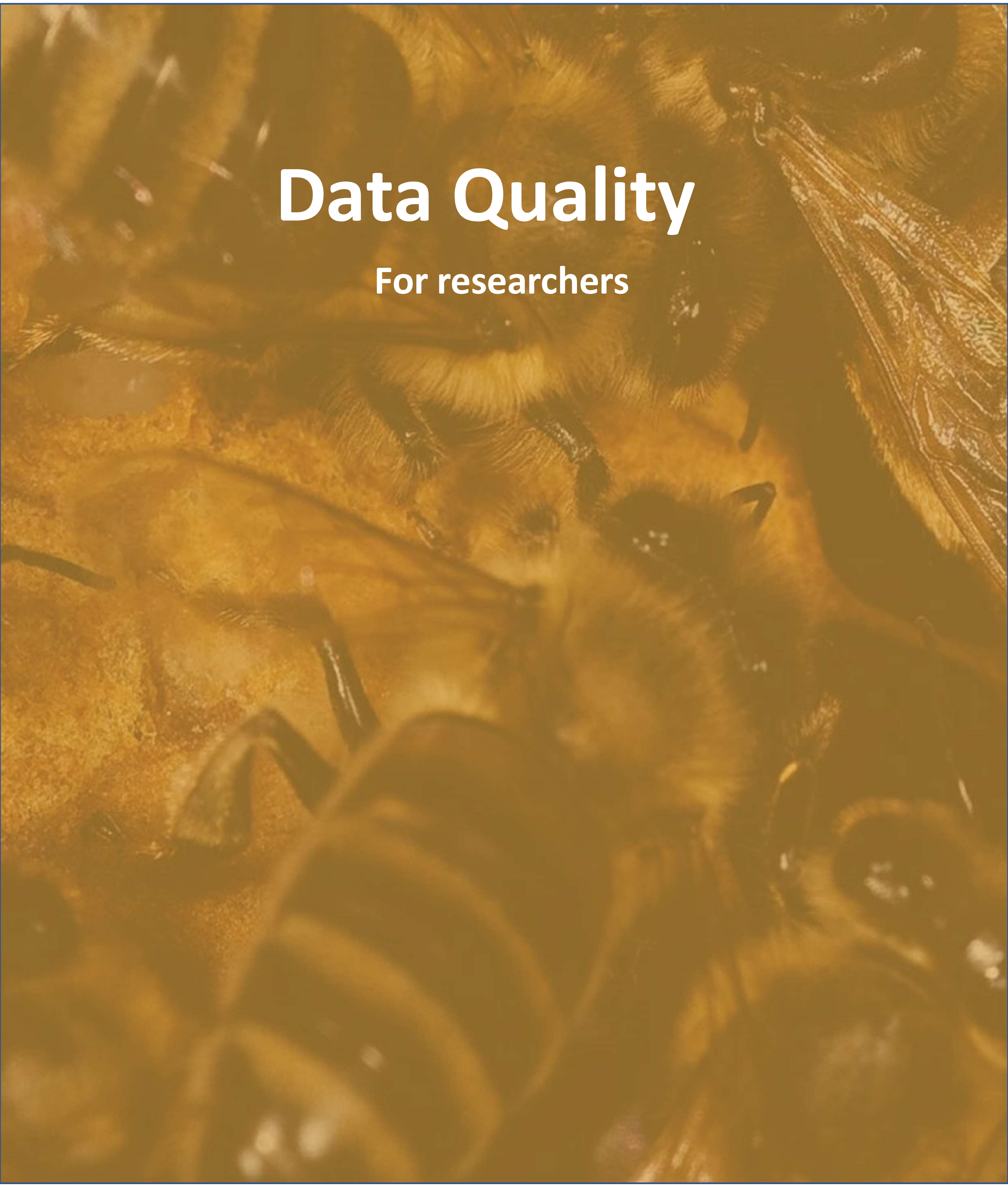

Data Quality

For researchers

This protocol was developed for B-GOOD project. B-GOOD stands for Giving Beekeeping Guidance by computational-assisted Decision making. The B-GOOD project received funding from the European Union's Horizon 2020 research and innovation programme under grant agreement number 817622.

B-GOOD protocol title and code: Data quality (P12)

Description: Checking and cleaning up data on the BEEP app

Version: 20220204

B-GOOD Tier: Tier 1

Target group: Researchers

Data quality and cleaning

To evaluate the data entry in the BEEP app, minimize discrepancies in sensor data and revise as necessary to ensure workable data.

Each month, to carry out the following:

- Within one week of the end of each month the inspection data for that month needs to be uploaded in the BEEP app by apiary managers.
- One week following the end of each month INRAE will generate an Excel report for each apiary manager for each of their colonies to describe the discrepancies, if any present, between automated data and inspection data. The report covers all data entered via the BEEP app as well as the automated data collected via the BEEP base.
- INRAE will arrange an online meeting with all apiary managers to go through any data issues they have experienced, or that are identified in the Excel report. If time allows the same meeting will be used to log interesting/unusual notes coming from the beekeeper(s) with regards to any (of the 8) colony health status.
- INRAE will make corrections to each month's data as necessary and the data will then be uploaded to the data portal where it can be accessed for purposes of B-GOOD. The apiary managers are encouraged to correct the data in the BEEP app as necessary.
- We ask that the apiary managers to check their own data weekly via the BEEP app to see if there are hardware or network errors that can be caught on a faster than monthly cycle.

No references

Acknowledgments

The authors of this protocol thank the B-GOOD Tier 1 and Tier 2 partners for their constructive feedback during the project duration to optimize the content for high quality data collection in the project. The B-GOOD project received funding from the European Union’s Horizon 2020 research and innovation programme under grant agreement number 817622.

B-GOOD partners:

This protocol was developed for B-GOOD project. B-GOOD stands for Giving Beekeeping Guidance by computational-assisted Decision making. The B-GOOD project received funding from the European Union’s Horizon 2020 research and innovation programme under grant agreement number 817622.
