# Supplementary material for: Bridging the Gap between Field Experiments and Machine Learning: The EC H2020 B-GOOD Project as a Case Study towards Automated Predictive Health Monitoring of Honey Bee Colonies
Source: Insects. 2024 Jan 22;15(1):76. doi: 10.3390/insects15010076 (PMC10816039; doi:10.3390/insects15010076)
Supplement: Supplementary file 1 [file insects-15-00076-s001.zip › S1_tier 2_workplans and protocols.pdf]

Workplan Tier 2

For beekeepers

Supplementary information S1 belonging to

Van Dooremalen C, Ulgezen ZN, Dall'Olio R, *et al.* Bridging the gap between field experiments and machine learning: The EC H2020 B-GOOD project as an example method to work towards automated predictive hive monitoring and healthy honeybee colonies.

This workplan was developed for B-GOOD project. B-GOOD stands for Giving Beekeeping Guidance by computational-assisted Decision making. The B-GOOD project received funding from the European Union's Horizon 2020 research and innovation programme under grant agreement number 817622.

B-GOOD workplan title: Workplan for B-GOOD selected beekeepers (TIER2, WP1)

Version: 20220203

B-GOOD Tier: Tier 2

Target group: Beekeepers guided by researchers

Comment: This is an exact copy of the workplan as it was used in the B-GOOD project

Workplans may be updated during the project period. Always ensure you have the latest version of the workplan.

Experiment Set-up

Five B-GOOD partners (Table 1) with a network of beekeepers are involved in TIER2. Each partner will recruit 8 beekeepers to participate in the experiment. Each beekeeper will keep three (presumably) healthy colonies at their apiary. The experiment will run for two years from January 2021 till April 2023.

Field study A (5 institutes x 8 beekeepers) x 3 colonies = 120 colonies (40 beekeepers)

The aim of the study is to:

1) Determine the most relevant parameters for assessing the health status of managed honey bee colonies;
2) Validate new technologies for automated measurements of health status.

This workplan facilitates the methods and protocols for all preparations and measurements during the experiment. As such, in three of your colonies we ask you to do some classical methods of measuring bee health by visual inspection, where colony traits such as colony size are recorded. For automated measurements you will be supplied with a BEEP system (3 BEEP bases and 1 BEEP app per beekeeper) to be used the allocated colonies. BEEP is a system built to support beekeepers, where observations of the hive can be digitally registered using a record keeping app, complemented by automatic observations using sensors.

Table 1: List of participants.

Participant organisation name	code	Country
Wageningen Research	WR	The Netherlands
Martin-Luther-Universitaet Halle-Wittenberg	MLU	Germany
Universitat Bern	UBERN	Switzerland
Suomen Mehiläishoitajain Liitto	SML	Finland
BeeSources di Raffaele Dall’Olio	BSOUR	Italy

BEEP System

BEEP app

We will use the BEEP app to collect and store data throughout the research period January 2021 to April 2023 in a standardized way for:

1. Registration and consent to use your data
2. Information on the apiary level
3. All management actions related to beekeeping
4. All experimental observations

Please go through the following steps related to the BEEP app, once or throughout the research period (depending on the step)

1. **Registration and consent to use your data** (once)

See Appendix 1 how to go to and register in the BEEP app and consent to participate in the research. We will make use of the 'web app' of the BEEP platform to log all information on the apiaries. Web app means that you can open the app on your mobile phone, tablet and computer and that you do not have to download anything.

2. **Entry of information on the apiary level** (once, but keep up to date during the research period)

In the BEEP app you need to store your meta data entry on the apiary level, e.g. location, colony numbers, type of hives, number of brood boxes. Select 3 of your colonies and enter their data into the app. If they are all in one location, you can create one apiary with the three hives*. If you allocate hives in different apiaries, make multiple apiaries in the app.

*you may use the app for all your hives if you wish. It would be very welcomed by us. Do realise that such additional hive (and inspection) information will also be shared automatically for research purposes if the hives are on the same account that you use for the B-GOOD project.

3. **Entering all management actions related to beekeeping** (throughout the research period)

Please record all management actions related to beekeeping that are done throughout the period January 2021 to April 2023 in the BEEP app. For example, this involves adding or removing of brood boxes or honey supers, splitting colonies, queen replacement, feeding, and all other actions. Inspection sheets for management actions should be made by the user. See BEEP manual for creating inspections sheets. All data must be recorded electronically. Use the appropriate fields to enter the data and only use the notes field if there are no other options, in order to ease data analysis. Please write the notes preferably in English. When you click the save button in the BEEP app, the data will be stored on the BEEP server. You can always access your own inspection data (see manual) and sensor data (in app click Menu>Measurements) in the app or by downloading it (see manual). See section *'Beekeeping Management'* in this document, for general instruction about the colony and hive management during the research period.

4. **Entering all experimental observations** (throughout the research period)

All experimental observations need to be entered in the BEEP app. For the experimental observations standard inspection sheets have been prepared for you, e.g. colony size using top photo method, mite fall. The inspection sheets can be found in the BEEP app (Figure 1, where to find them in the app). In the section '*Experimental Observations*' of this workplan, you can find information on when and which protocols and inspection sheets should be used throughout the measurement period January 2021 – April 2023. The inspection sheets can only be seen and used, after consenting to participate in the research (Step 1).

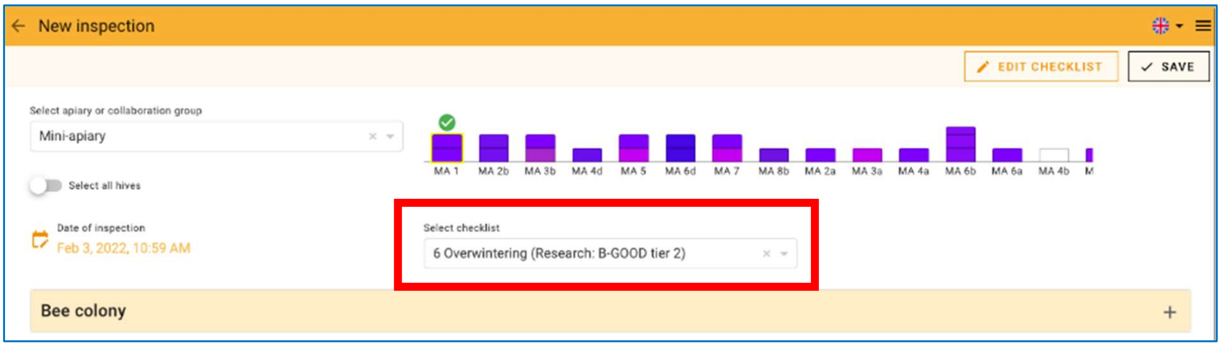

Figure 1: The section for selection of checklist for data entry. Select the appropriate checklist for data collection depending on type of experimental observation and timing of data collection (see Table 2).

5. **Some general remarks about the BEEP app**

When you click the save button in the BEEP app, the data will be stored on the BEEP server. Each participant will have continuous access to their own collected and stored data. WR will have access to all data collected in field study A and will process the data for further use together with the BEEP team.

At each hive inspection, the BEEP app on your phone or computer must be connected to the internet to send inspection data to the server. The BEEP app automatically registers the date and time when you enter data into the system. Information to the app can also be added at a later date. However, it is important to change and correct for the date of inspection, and adjust it to the original moment of data collection if data is entered at a later date (Figure 2).

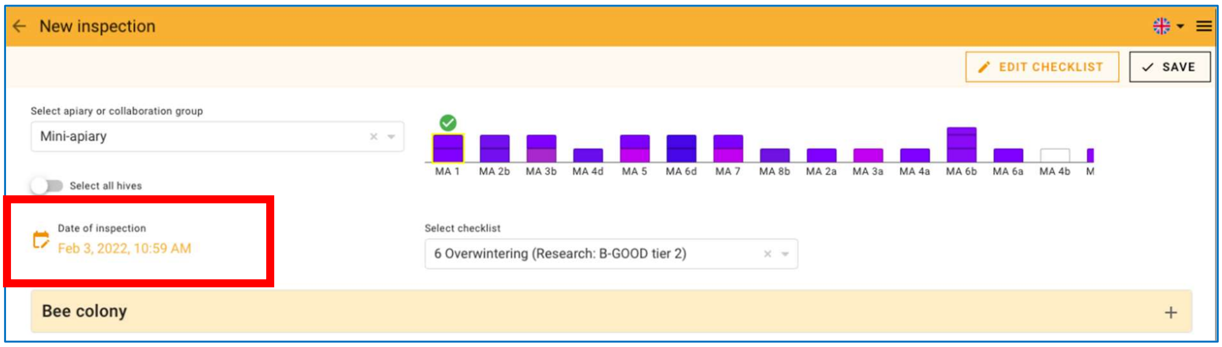

Figure 2: The section for entry of date of inspection (observations and actions) in the BEEP app. If data is entered at a later date adjust it to the original moment of data collection.

BEEP base and sensor placement

At the start of the experiment, you will get 3 BEEP bases (Figure 3) to install under your selected hives. During the experiment we will continuously monitor hives with the BEEP base, which measures weight, temperature and sound in the hive. BEEP will provide an installation manual that includes information on the set-up of the BEEP base (for online version click here). See below for sensor placement in the hive.

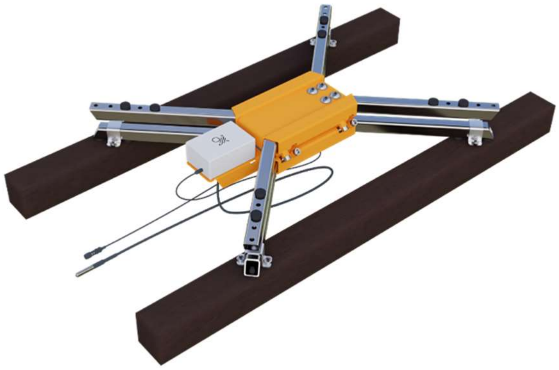

Figure 3: The BEEP base that will be placed under each of the 3 hives in your apiary after you installed the colonies. **Note that the computer (white box) is placed to the side. The BEEP base dimensions are such that most hives fit well and stable when the BEEP base is placed this way. Turning it 45 degrees is not preferred.**

Scale. A weight sensor is at the centre of the steel construction of the BEEP base. The BEEP base needs to be placed underneath the hive for continuous weight measurements (Figure 4).

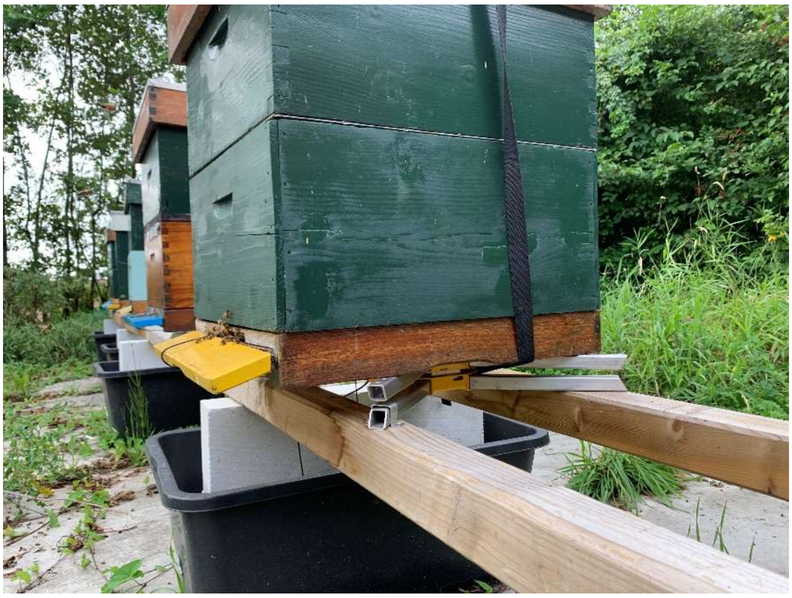

Figure 4: Placement of weight sensor underneath the hive.

Thermo-sensor. A thermo-sensor is connected through a cable to the BEEP base, and will be used for continuous temperature measurements. The sensor needs to be placed in the brood box, on top, between the mid-frames (Figure 5). If the hive has several brood boxes, and there is a queen excluder, keep the temperature sensor in the box with the queen. If there is no queen excluder, always keep the sensor in the top brood box.

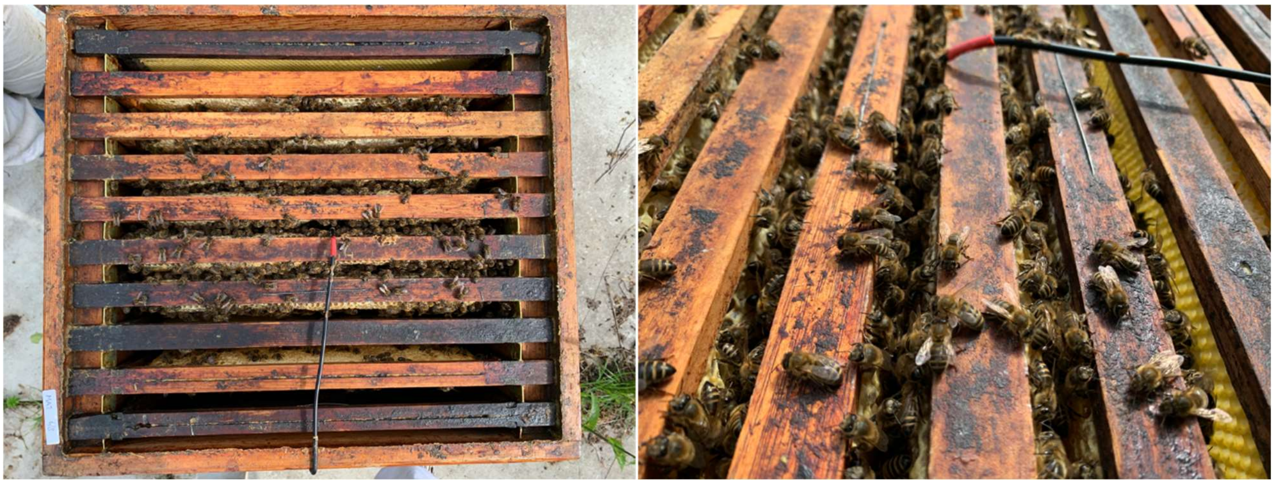

Figure 5: Placement of temperature sensor in the hive. It is approximately 9 cm from the red tape to the tip of the temperature sensor.

Microphone. A microphone is connected through a cable to the BEEP base, and will be used for constant sound measurements. The sensor needs to be placed at the center of the bottom frame, facing the back of the hive, opposite to the hive entrance (Figure 6).

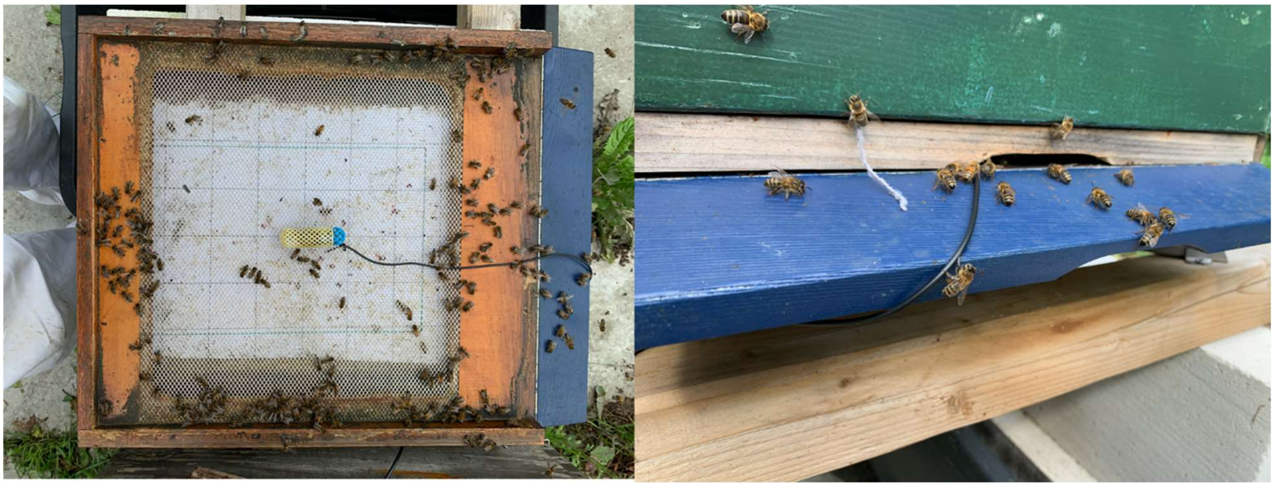

Figure 6: Placement of microphone in the hive. The cable can be placed through the flight entrance.

Beekeeping management

In the beginning of the experiment, three colonies need to be allocated to field study A and need to be kept with the tools (BEEP base) at all times. The criteria for keeping colonies is as follows:

- Colonies can be replaced by a new colony in case they die. This must be clearly indicated in the BEEP app.
- The colonies can be moved to a new location but not sold.
- The colonies should preferably be local honey bees, but the use of imported queens is also fine.
- The colonies should be kept at a location with cellular network and data connection (2G, 3G or 4G) for data collection purposes with BEEP base.

The basic principle of management actions within Field study A will be to: maintain healthy colonies; while at the same time respecting the nature of the bees; and providing care accordingly.

We will not provide you with standard beekeeping protocols or guidelines. The colonies should be managed accordingly with your regular beekeeping practices. However, all decisions made should align with the following criteria:

1) Colonies should be kept healthy
2) Enter all actions and changes in the BEEP app (e.g. swarm prevention, splitting, providing space, queen replacement, merging, reduce brood boxes, removal of brood, addition of bees and provision of nutritional supplements, etc) in inspections for the appropriate hive. Please ensure the correct date and time is entered for actions. See section *'BEEP app'*, step 2 and 3.
3) If a colony dies, take a sample of bees (and perhaps brood also, if brood diseases are expected) and store until the regular sampling will take place (see experimental measurements)
4) If honey is harvested, then record how much honey is harvested per hive and the date of harvest (in Inspection under Production > Honey). You may exclude the weight of wax seals from the calculations (automatically adding that weight to the honey harvest) in case you calculate the kg honey by: kg of the full honey super at harvest - kg of the empty super and empty frames after honey extraction.

Data collection: experimental observations

Table 2 (see page 10) gives the overview of required experimental measurements over time and the related inspection sheets that we have prepared. Please stick to the planning as much as possible. Detailed information about methods for data collection are provided in protocols that will be incorporated in the app. Additional information about the data collection methods that is needed throughout the experiment is detailed below (with the related protocol number between brackets). Try combining activities to minimize colony disturbance.

Please be aware that the experimental measurements detailed below are for the period of January-December 2022. At the end of each year we update the protocols. Based on the outcomes of the previous year, data collection techniques may slightly change in order to improve and fine-tune methods of assessing colony health for the coming year. If after assessments, a protocol is considered to be non-useful or surplus it will be discarded from the experiment.

Presence of queen and brood (P1.2)

Every four weeks starting from the end of the winter period till the end of the beekeeping season (essentially, the period of honey bee foraging activity), the hive comb surface will be checked for the presence of queen and of all stages of brood (BIAS, brood in all stages). Keep a record in BEEP app of queen presence, brood presence and queen supersedure.

Top photo analysis (P3.2)

Colony size will be estimated by taking a photo of the topside of the hive. This will be done throughout the whole year, once a month, preferably the first week of each month. At times of temperatures below -5°C, it may be better to postpone or even skip this measurement.

Sampling bees for lab analyses (P5.2)

Lab analysis on diseases and genotyping will be done through Reference Labs in Germany and Belgium. For this, samples of bees will be collected three times a year. First in spring, when the bees start to forage; second time in summer, when the colonies have reached their maximum size; and a third time in autumn, before the over-wintering. As spring, summer and autumn will be shifted between participating beekeepers, the sampling moments need to be adjusted by participants depending on the climate of country. The first (spring) visit should take place as soon as major pollen producing plants are flowering. Look out e.g. for willow plants *(goat willow or gray willow*). The spring visit should take place within three weeks from the moment these plants start flowering. The second (summer) visit should take place about 2 weeks after midsummer, or if known, the moment of peak colony size that naturally occurs in region. The third (autumn) visit should take place before it gets too cold to sample (< 10°C). Depending on the location of the apiary, the visiting time can be from around the end of September until the end of November.

Clinical signs of disease (P7.2)

During the beekeeping season, check the hive at least once a month for clinical signs of disease. If you have the suspicion that a disease is present in the hive, record the information in the BEEP app (in Inspection under Disorder > Type). Potential diseases that you may observe in colonies are: varroosis (Varroa mites), American Foulbrood (*Paenibacillus larvae*), European Foulbrood (*Melissococcus plutonius)*, nosemosis (*Nosema* spp.), Acute Bee Paralysis Virus or Chronic Bee Paralysis Virus, Black Queen Cell Virus, Deformed Wing Virus, Sacbrood virus and maybe (but hopefully not) small hive beetle (*Aethina tumida*).

Queen cell presence (P10.2)

Presence of queen cells in colonies provides insight on reproduction (swarming tendency) and/or queen quality. Once a month during the beekeeping season, especially after the end of swarming season, check colonies for presence of queen cells. Indicate in the BEEP app the type of queen cell present (queen cup, emergency cell, swarm cell, supersedure cell). If queen cells are removed, please include this information as a management action.

Drone presence (P13.2)

During swarming season, beginning in spring from the start of bee foraging activity, check colonies for presence of drone brood. Continue checking until all of the drones have died and the reproductive phase is finished. The presence of drone brood gives an indication of swarming tendency in colonies. Colonies should be checked for drone brood at least 1x a month or each time the colony is opened.

Colony mortality

Throughout the year, information on colony mortality and observations on dead colonies should be recorded. A colony is considered dead if 1) the hive is absent of any living bees 2) the colony is too weak to recover in spring because (i) less than two frames are occupied by winter bees or (ii) the queen is dead and the hive cannot replace queen by building emergency cells (no brood). In case a colony dies, replace it with a new one and record actions in BEEP app. If possible, take a sample of the remaining dead bees and store until the upcoming sampling round for disease (P5) and include this sample in the batch, with a special marking "Dead" and the (approximate) date it died.

Overall impression

Information on the overall impression of the health of each colony will be collected throughout the whole year. There are three different categories in the BEEP app for the health status (Figure 7). Description or definition of categories will not be provided as this is a subjective measurement by the observer. Entry of data should be done at least once a month, but can also be done more frequently, up to once a week. This measurement is included at the end of each inspection sheet (see Appendix 1). If you use multiple inspection sheets during one inspection, filling in the overall impression on only one of the inspection sheets will suffice.

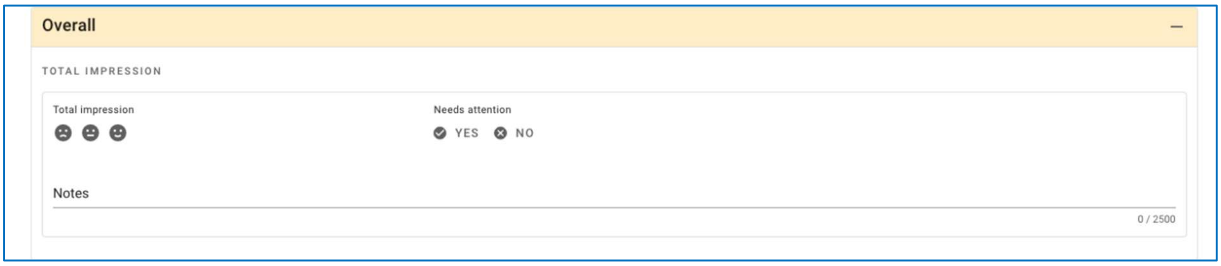

Figure 7: Data entry for overall impression on the health of colonies in the BEEP app.

Colony Health

Information on the health status of the colony will also be collected with the Healthy Colony Checklist (Figure 8). Similar to the *Overall impression*, a description or definition of categories will not be provided as this is a subjective measurement by the observer. Entry of data should be done at least once a month, but preferably should be done as often as possible, up to once a week (Table 2). At some time points you will have more information available (e.g. after checking for queen presence and BIAS) and can give a more reliable beekeeper assessment, compared to other time points (e.g. after top photo). Both types of time points are valuable.

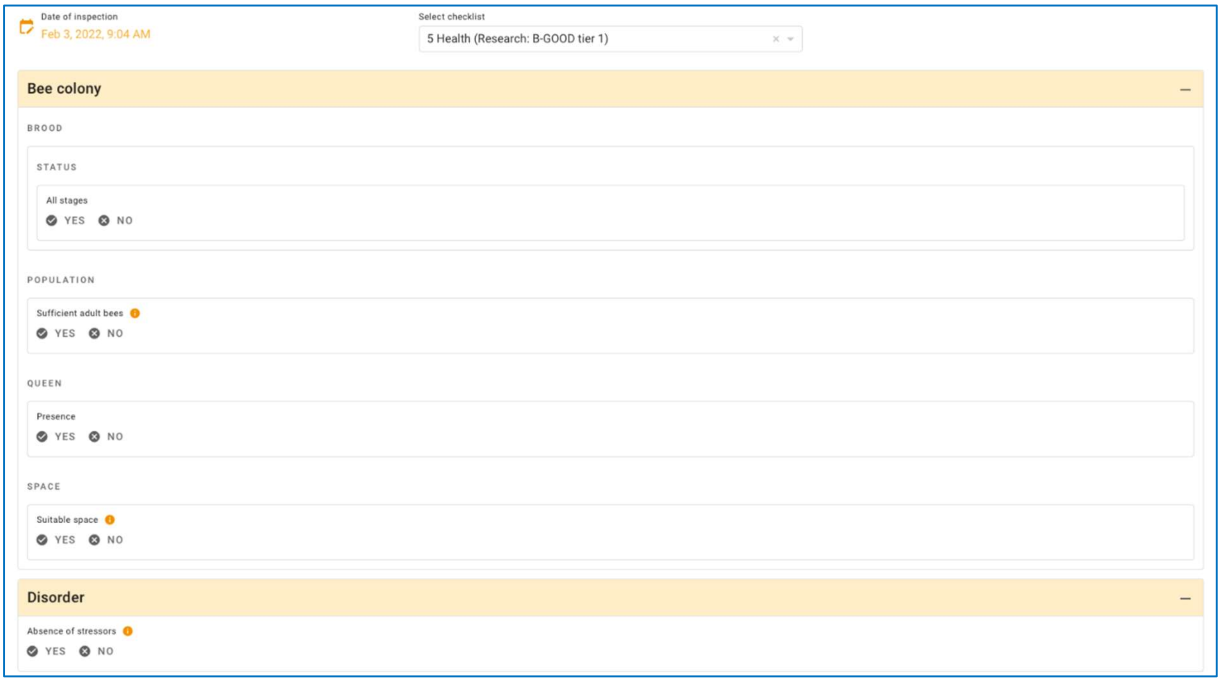

Figure 8: Part of the data entry for data annotation in the BEEP app (inspection sheet Health).

Mite infestation level

Counting Varroa for mite infestation levels in colonies is not mandatory. However, if you regularly count mites in your colonies by natural mite fall or alcohol/soap/sugar method, please enter this data in the BEEP app (in Inspection under Varroa>Disorder>Count).

Acknowledgments

The authors of this workplan thank the B-GOOD Tier 1 and Tier 2 partners for their constructive feedback during the project duration to optimize the content for high quality data collection in the project. The B-GOOD project received funding from the European Union's Horizon 2020 research and innovation programme under grant agreement number 817622.

B-GOOD partners:

This workplan was developed for B-GOOD project. B-GOOD stands for Giving Beekeeping Guidance by computational-assisted Decision making. The B-GOOD project received funding from the European Union's Horizon 2020 research and innovation programme under grant agreement number 817622.

Table 2 – Overview of experimental observations over time

Overview of experimental observations over time. The protocol number, the timing (frequency) of measurements and the months in which they are to be performed are provided for each activity. Coloured cells show in which months experimental observations are expected to be performed. Three inspection sheets were prepared for use in the BEEP app, covering the different protocols: 6 Overwintering; 7 Bee Season; 8 Bee Season+

			**Months**											
**Experimental observation**	**Protocol**	**Timing**	*Jan*	*Feb*	*Mar*	*Apr*	*May*	*Jun*	*Jul*	*Aug*	*Sep*	*Oct*	*Nov*	*Dec*
Presence of queen & brood	P1.2	1 x month *												
Top photo analysis	P3.2	1 x month												
Sampling for lab analyses	P5.2	3 x year *												
Clinical signs of disease	P7.2	1 x month *												
Queen cell presence	P10.2	1 x month *												
Drone presence	P13.2	1 x month *												
Overall impression	NA	1 x week **												
Colony Health	NA	1 x week **												
Colony mortality	NA	1 x month *												
**BEEP app inspection sheets to be used**			**6**	**6**	**6**	**8**	**7**	**7**	**8**	**7**	**7**	**8**	**6**	**6**

** The months may vary between countries, dates are only provided as an outline, and to represent activities that can be combined. Participants should make 'local' decisions on timing of data collection, depending on colony status, phenological state and climate of country. See workplan section on experimental observations for more details.*

*** Preferably should be done once a week, but acceptable to reduce to once a month in case apiary is in a remote area or has difficult access.*

Appendix 1 – BEEP app registration

To start using BEEP for the B-Good project, please follow these steps:

Register in BEEP

- In the manual you can find out how you can place the BEEP app on your phone or tablet. You only need one account and with that account you can access BEEP on multiple devices.
- REGISTER: Go to the BEEP app, using a web browser on a computer or alternatively on a mobile phone via this link for the English version. As a new user, click on the login screen on 'No account yet? Register as a new user'. Register with your work email address and follow the instructions. See the login support article for more information on this step.
- APIARY: When logging in for the first time, you will see the 'Create new apiary' screen where you can add the B-GOOD apiary details. When you are done, click on 'Create new apiary' button to save the data. See the Create a new apiary article for more information on this step.
- HIVES: Open the apiary you created. You can change the settings per hive, by clicking on the hives. You can change the configuration and enter the details on the queens per hive. See this article for more information on this step.
- INSPECTIONS: By clicking on the pen icon under each hive, you can add inspections for that hive. This is also further described here.
- RESEARCH: An important step is to link your account to the B-GOOD research program. You only need to do this once. You can click on 'Research' in the menu on the left and select the B-GOOD program by following the on-screen instructions (Figure 9). This way the data can be accessed for analysis in WP1.
- COLLABORATION: your co-workers can edit the data for your apiaries also if needed. You can see here how you can set this up for your group.

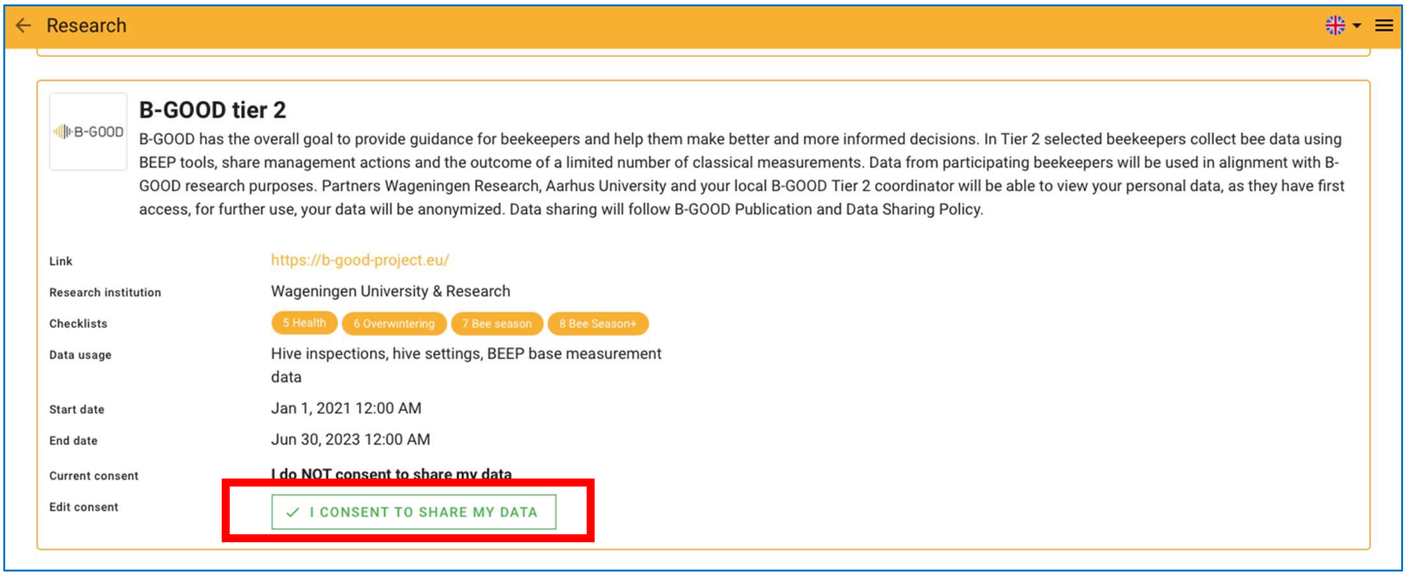

Figure 9: After creating your account on the BEEP platform, you can link your account to the B-GOOD programme by clicking on 'Research' in the menu.

B-GOOD

WAGENINGEN UNIVERSITY & RESEARCH

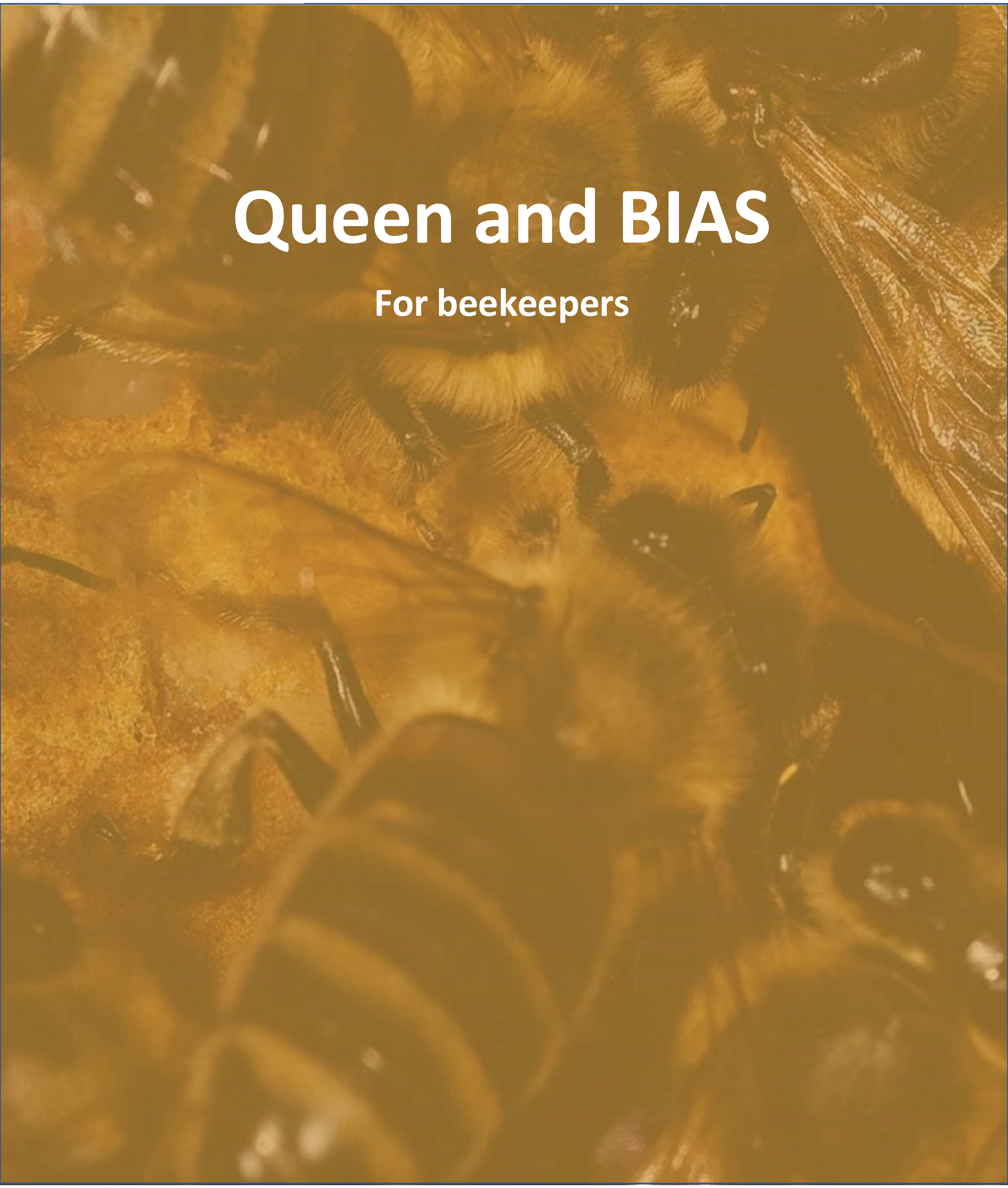

Queen and BIAS

For beekeepers

This protocol was developed for B-GOOD project. B-GOOD stands for Giving Beekeeping Guidance by computational-assisted Decision making. The B-GOOD project received funding from the European Union's Horizon 2020 research and innovation programme under grant agreement number 817622.

B-GOOD protocol title and code: Queen and BIAS (P1.2)

Description: Finding the queen and checking brood iIn all stages

Version: 20220513

B-GOOD Tier: Tier 2

Target group: Beekeepers

Presence of the queen and brood

Every four weeks starting from the end of the winter period till the end of the beekeeping season (essentially, the period of honey bee foraging activity), check the hive comb surface for the presence of queen and of all stages of brood.

Field methods

- Open a colony and sequentially remove frames from the hive.
- Check the hive comb surface until the presence of queen and of all life stages of brood –eggs, larvae, pupae – are verified. The queen should be labelled for easy detection.
- If queen is not found, and/or no open brood is present, queen failure is assumed (after rechecking in 1 week). Queen presence can be presumed if eggs are present.
- Please be aware that the queen might stop laying eggs prior to swarming, in early winter and during extreme weather events.
- Record queen presence, brood presence and any replacement queens in the BEEP app.

References

Delaplane KS, Van Der Steen J, Guzman-Novoa E (2013). Standard methods for estimating strength parameters of *Apis mellifera* colonies. Journal of Apicultural Research 52: 1-12.

EFSA AHAW Panel (EFSA Panel on Animal Health and Welfare) (2016). Scientific opinion on assessing the health status of managed honeybee colonies (HEALTHY-B): a toolbox to facilitate harmonised data collection. EFSA Journal 14: 4578, 241 pp.

Human H, Brodschneider R, Dietemann V, *et al*. (2013). Miscellaneous standard methods for *Apis mellifera* research. Journal of Apicultural Research 52: 1-53.

Acknowledgments

The authors of this protocol thank the B-GOOD Tier 1 and Tier 2 partners for their constructive feedback during the project duration to optimize the content for high quality data collection in the project. The B-GOOD project received funding from the European Union's Horizon 2020 research and innovation programme under grant agreement number 817622.

B-GOOD partners:

This protocol was developed for B-GOOD project. B-GOOD stands for Giving Beekeeping Guidance by computational-assisted Decision making. The B-GOOD project received funding from the European Union's Horizon 2020 research and innovation programme under grant agreement number 817622.

B-GOOD

WAGENINGEN UNIVERSITY & RESEARCH

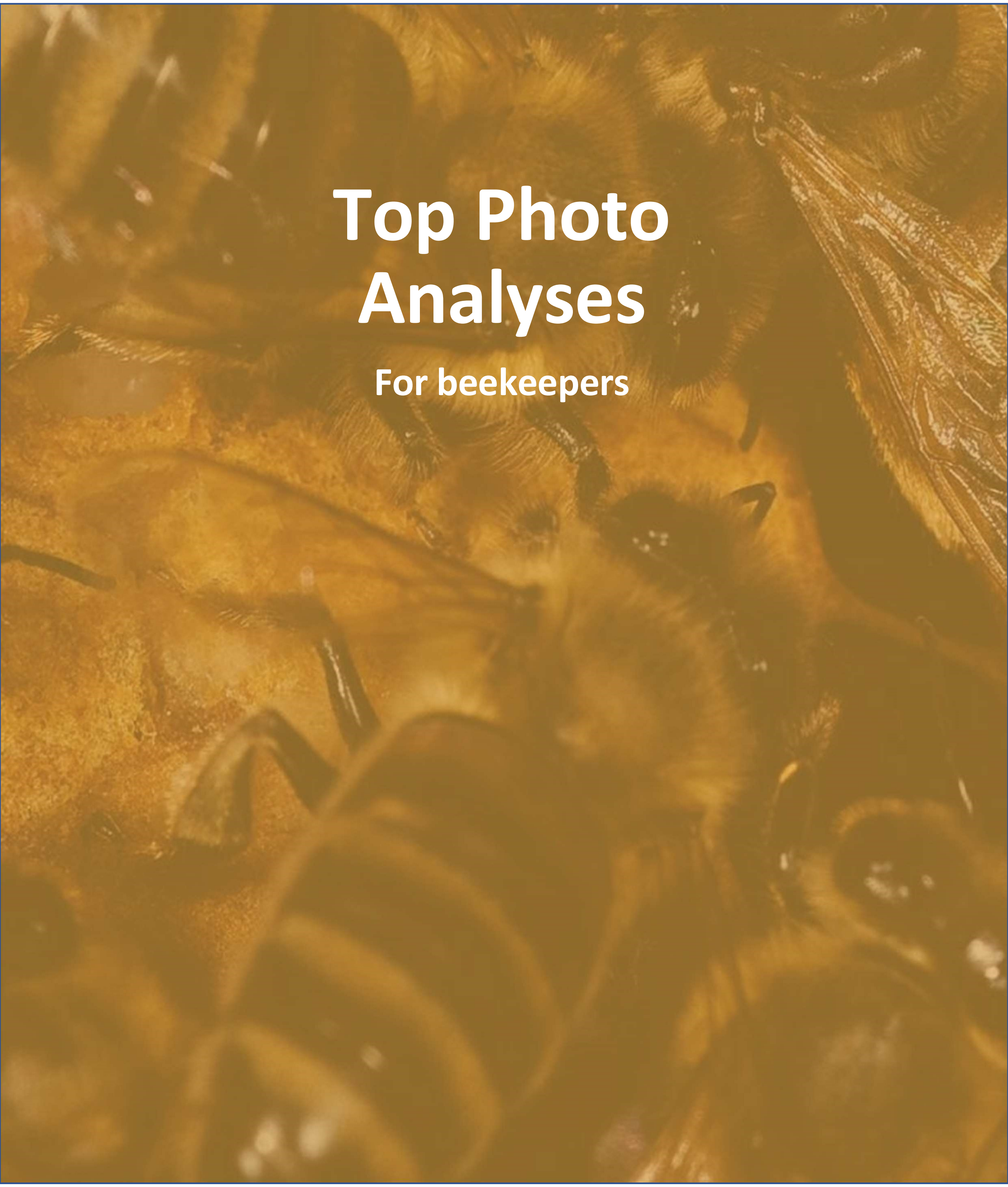

Top Photo Analyses

For beekeepers

This protocol was developed for B-GOOD project. B-GOOD stands for Giving Beekeeping Guidance by computational-assisted Decision making. The B-GOOD project received funding from the European Union's Horizon 2020 research and innovation programme under grant agreement number 817622.

B-GOOD protocol title and code: Top photo analyses (P3.2)

Description: Analyses of colony sizes by taking pictures of broodbox from the top

Version: 20220513

B-GOOD Tier: Tier 2

Target group: Beekeepers

Top photo analysis

Colony size will be estimated by taking a photo of the topside of the hive. This will be done every first week of each month throughout the whole year.

Materials

- Camera – preferably DSLR
- Bee smoker

Field methods

- Blow a puff of smoke into the hive from below.
- After a minute, remove the lid and take first a high-resolution photo of your label/number/code of the hive and then of the top-side. For the accuracy of the photo analysis, take care to include the entire set of top frames in the photo, and use a standard angle lens (Figure 1).
- If there is more than one brood box:
    - Only take a photo of the top box and make sure that the number of boxes entered in the BEEP app is correct.
    - If you suspect that one of the brood boxes is empty of bees, then reduce and adjust the number accordingly in the app.

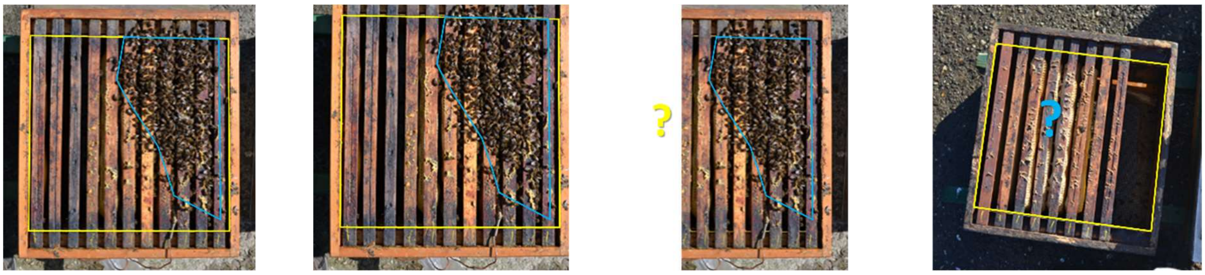

CORRECT | CORRECT | WRONG (does not include all the frames) | WRONG (bees are too deep and not visible)

Figure 1: Correct position of hive top in photograph and potential mistakes to avoid. Yellow lines show size of the top side of the box and blue lines show subjective estimation of top side of the bee cluster, if possible to estimate. Both lines are necessary to estimate the number of bees in the colony.

Computer Analysis

- Measure the available area and area occupied by bees using the software ImageJ. (https://imagej.nih.gov/ij/). Calculate the fraction covered in bees based on the number of pixels in a colony. To do so, calculate the ratio between the number of pixels of the area covered with bees (on top and visible between the frames) and the overall number of pixels of the top area of the box that represents the inner side of the box (see also yellow markings in Figure 1).
- Please see B-GOOD tutorial for a how-to video. (click here for tutorial)
- Enter the information of 'pixels with bees' and 'pixels total top' into the BEEP app.
- Upload the photo when entering data in the BEEP app.

References

Van Dooremalen C, Cornelissen B, Poleij-Hok-Ahin C, *et al*. (2018). Single and interactive effects of *Varroa destructor*, *Nosema spp*., and imidacloprid on honey bee colonies (*Apis mellifera*). Ecosphere 9: e02378.

Van Dooremalen C, Van Langevelde F (2021). Can colony size of honeybees (*Apis mellifera*) be used as predictor for colony losses due to *Varroa destructor* during winter? Agriculture 11: 529.

Acknowledgments

The authors of this protocol thank the B-GOOD Tier 1 and Tier 2 partners for their constructive feedback during the project duration to optimize the content for high quality data collection in the project. The B-GOOD project received funding from the European Union's Horizon 2020 research and innovation programme under grant agreement number 817622.

B-GOOD partners:

This protocol was developed for B-GOOD project. B-GOOD stands for Giving Beekeeping Guidance by computational-assisted Decision making. The B-GOOD project received funding from the European Union's Horizon 2020 research and innovation programme under grant agreement number 817622.

B-GOOD

WAGENINGEN UNIVERSITY & RESEARCH

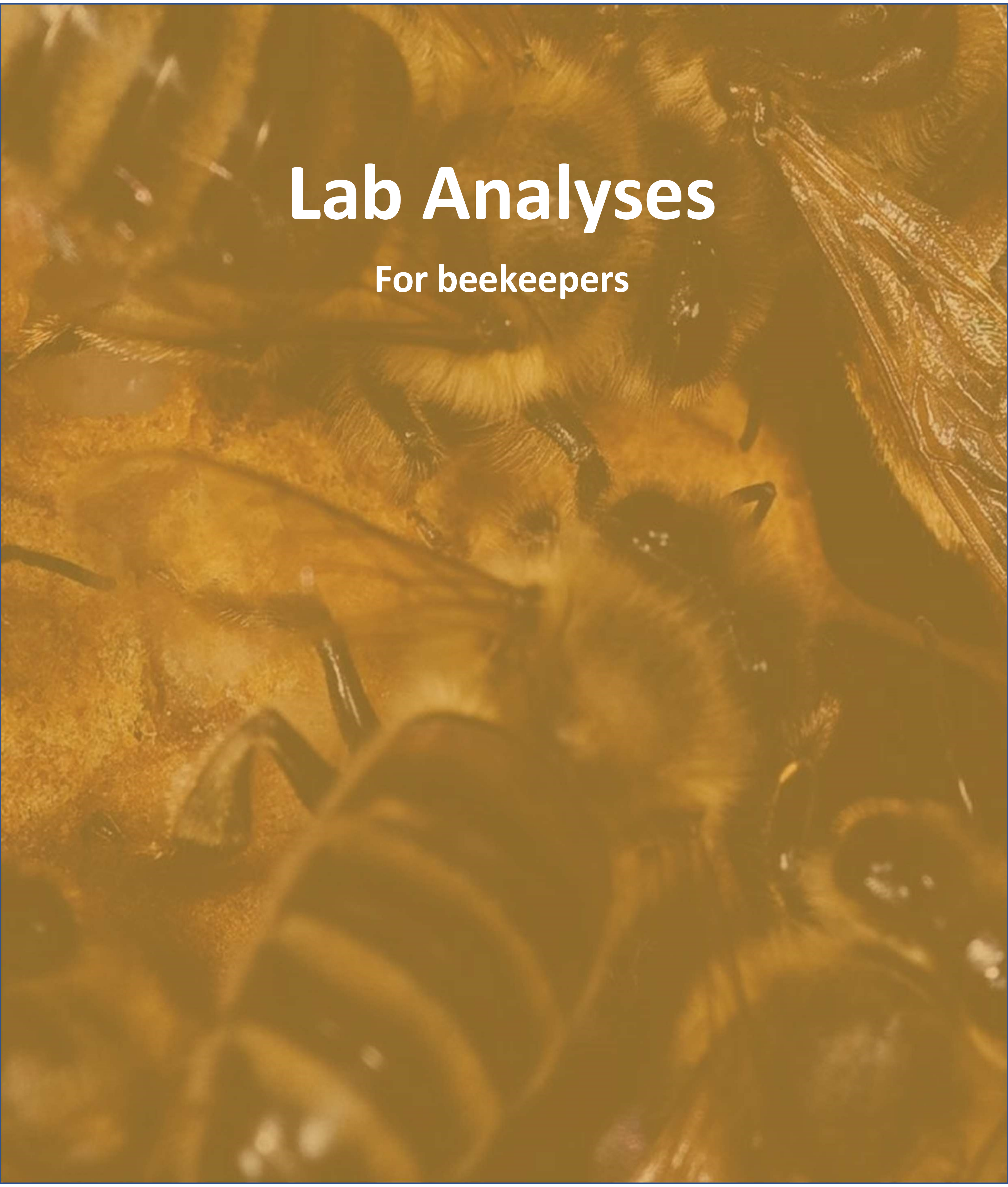

Lab Analyses

For beekeepers

This protocol was developed for B-GOOD project. B-GOOD stands for Giving Beekeeping Guidance by computational-assisted Decision making. The B-GOOD project received funding from the European Union's Horizon 2020 research and innovation programme under grant agreement number 817622.

B-GOOD

WAGENINGEN UNIVERSITY & RESEARCH

B-GOOD protocol title and code: Lab analyses (P5.2a)

Description: How to sample bees sent for lab analysis for diagnostic purposes (beekeepers)

Version: 20220513

B-GOOD Tier: Tier 2

Target group: Beekeepers

Sampling bees for lab analyses - beekeepers

For diagnosis of bee diseases each colony will be sampled three times per year: 1) first in spring, when the bees start to forage; 2) second in summer, when the colonies have reached their maximum size; 3) and third in autumn before the over-wintering.

BEEP app

- For anonymization purposes, the BEEP app will generate a unique identifier per sample. The samples should be labelled with these IDs. Please label the samples on the container and additionally place a label inside the container (on paper with a pencil), to ensure identification of the sample in case the outside label gets damaged or disappears during transport.
- Make sure the samples collected are correctly linked to the IDs to ensure correct feedback from the lab.
- Record the date of sample collection in the BEEP app.
- Check that the date and time of the inspection containing the sample code in the BEEP app corresponds with the actual sample collection date and time.

Materials

- 3 x 0.5 L freezer containers with drilled holes (max. 2 mm) (Figure 1)
- carton queen shipping box (with ventilation openings)
- 3 x queen cages with sugar dough in it

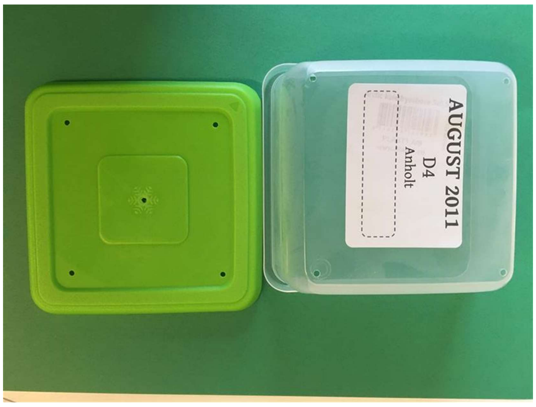

Figure 1: Example of freezer container with holes.

Collection of samples

- To collect samples, first open the colony and check the combs starting with the frames on the outer edge.
- Remove the first frame fully occupied by bees (most likely this will be a frame at the periphery of the brood nest).
- Make sure that the queen is not present on the comb, if present return her to the hive (or take another frame).
- Fill the freezer container with about 300 bees (a 100ml cup or 40g without container are about 300 bees).
- To keep bees alive, make sure that there are holes in the container and/or on the lid.
- One of the best methods to do this is to shake the bees from the frame onto a sheet, then bend or fold the sheet and use it to fill the bees into the cup. Many colonies have a covering foil on top underneath the lid. One can use this sheet but you can also bring a sheet or use a sheet of newspaper. The cup should be placed on the top of the open colony so all bees that spill out of the cup fall directly back into the colony. With this method, you can easily fill the cup to the top.
- Alternatively, if the bees are calm, fill the cup by scraping the bees off the comb, holding the cup vertically and the comb at 45°.
- It is fine if drones are included in the sample but less is better.
- Place the queen cage with sugar dough into the container to equip bees with some food prior to transport.

Transport

- Place the freezer containers with bees into a queen shipping box to prepare for transport. Please make sure that the containers are firmly closed and cannot open during transport.
- Coordinate the dates of transfer for samples with the B-GOOD partner organisation in your country before arranging transport.
- The samples should be sent the same day of sampling in the field.

References

Human H, Brodschneider R, Dietemann V*, et al*. (2013). Miscellaneous standard methods for *Apis mellifera* research. Journal of Apicultural Research 52: 1-53.

Matthijs S, Sciensano, BE-NRL Bee diseases. Pers. Comm. 2020

Schäfer MO, Friedrich-Loeffler-Institut, DE-NRL für Bienenkrankheiten. Pers. Comm. 2020

Van Dooremalen C, Wageningen Research. Pers. Comm. 2020

Acknowledgments

The authors of this protocol thank the B-GOOD Tier 1 and Tier 2 partners for their constructive feedback during the project duration to optimize the content for high quality data collection in the project. The B-GOOD project received funding from the European Union's Horizon 2020 research and innovation programme under grant agreement number 817622.

B-GOOD partners:

This protocol was developed for B-GOOD project. B-GOOD stands for Giving Beekeeping Guidance by computational-assisted Decision making. The B-GOOD project received funding from the European Union's Horizon 2020 research and innovation programme under grant agreement number 817622.

Lab Analyses

For researchers

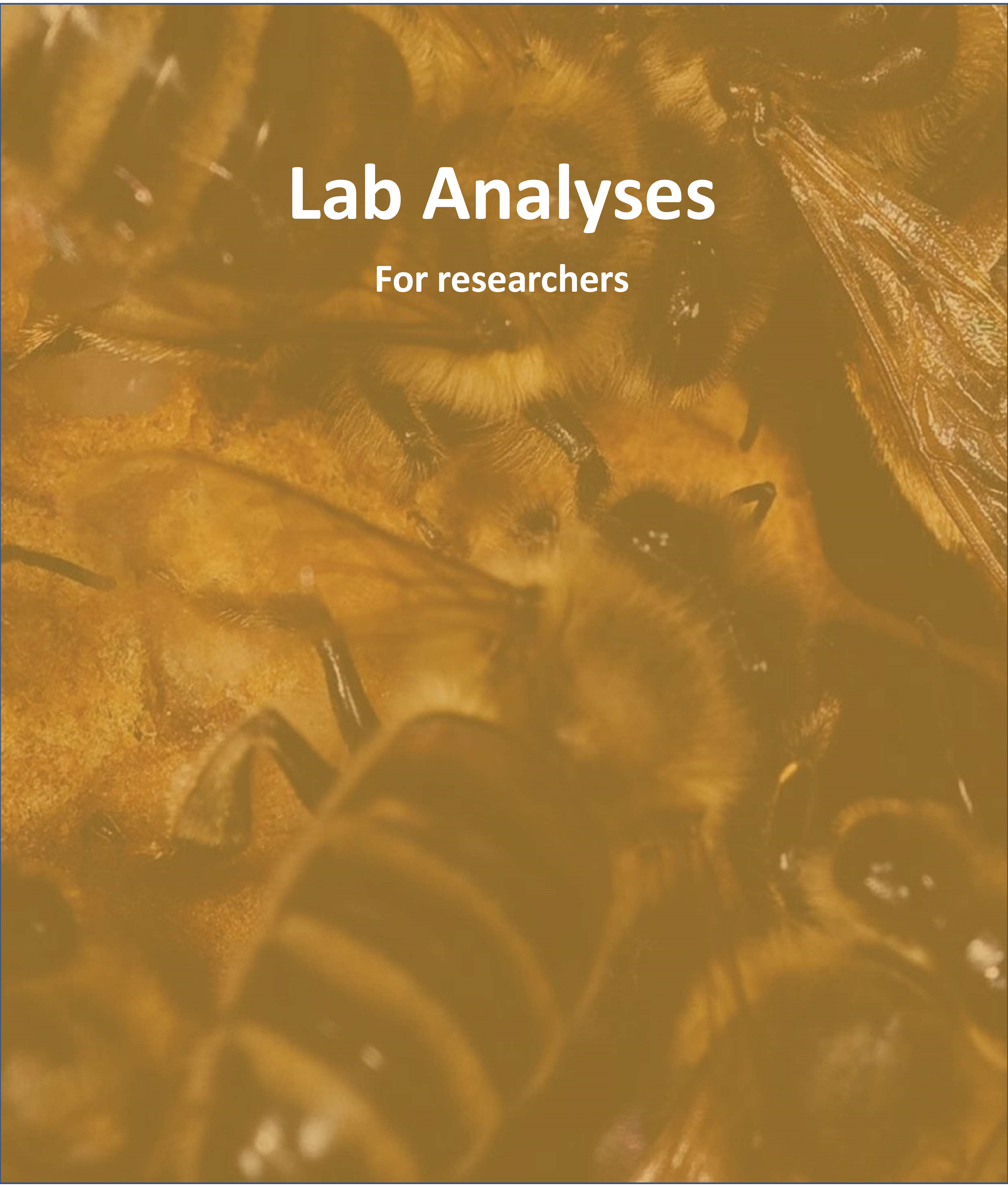

This protocol was developed for B-GOOD project. B-GOOD stands for Giving Beekeeping Guidance by computational-assisted Decision making. The B-GOOD project received funding from the European Union's Horizon 2020 research and innovation programme under grant agreement number 817622.

B-GOOD protocol title and code: Lab analyses (P5.2b)

Description: How to sample bees for lab analysis for diagnostic purposes (TIER2 partners)

Version: 20220513

B-GOOD Tier: Tier 2

Target group: Researchers that guide beekeepers

Sampling bees for lab analyses – TIER2 partners

For diagnosis of bee diseases each colony will be sampled three times per year: 1) first in spring, when the bees start to forage; 2) second in summer, when the colonies have reached their maximum size; 3) and a third in autumn before the over-wintering.

Storage & transport

- Once you receive samples from beekeeper, note if the shipping was successful and whether the bees are still alive.
- Immediately freeze the bees by either using liquid nitrogen, dry ice or placing containers in a freezer at preferably -80°C until dry-ice shipping. If -80°C is not available, the samples can alternatively be stored at -20°C.
- After freezing, place a solid lid (no holes) on the container or exchange the container for one with a solid lid to prepare samples for storage and transport. Please make sure that the labelling is correct and will withstand the freezing (best is label inside the container on paper with a pencil). Please also write the code on the lid.
- As soon as the sample is frozen, it should never defrost until the moment of RNA or DNA extraction. Therefore, it is important to establish a continuous cold chain for the samples.
- The transfer of samples for lab analysis must be done on dry ice, by courier services that enable fast transport. Arrival of samples should be within 48 h from the moment that they are sent.
- Send all samples (8 beekeepers x 3colonies = 24samples) from each sampling instant together.
- Partners should coordinate the sending date of samples with the B-GOOD labs before arranging transport. Make sure that samples do not arrive at the labs on weekends.
- Samples should be sent together with the signed MTAs.

References

Human H, Brodschneider R, Dietemann V, *et al*. (2013). Miscellaneous standard methods for *Apis mellifera* research. Journal of Apicultural Research 52: 1-53.

Matthijs S, Sciensano, BE-NRL Bee diseases. Pers. Comm. 2020

Schäfer MO, Friedrich-Loeffler-Institut, DE-NRL für Bienenkrankheiten. Pers. Comm. 2020

Van Dooremalen C, Wageningen Research. Pers. Comm. 2020

Acknowledgments

The authors of this protocol thank the B-GOOD Tier 1 and Tier 2 partners for their constructive feedback during the project duration to optimize the content for high quality data collection in the project. The B-GOOD project received funding from the European Union's Horizon 2020 research and innovation programme under grant agreement number 817622.

B-GOOD partners:

This protocol was developed for B-GOOD project. B-GOOD stands for Giving Beekeeping Guidance by computational-assisted Decision making. The B-GOOD project received funding from the European Union's Horizon 2020 research and innovation programme under grant agreement number 817622.

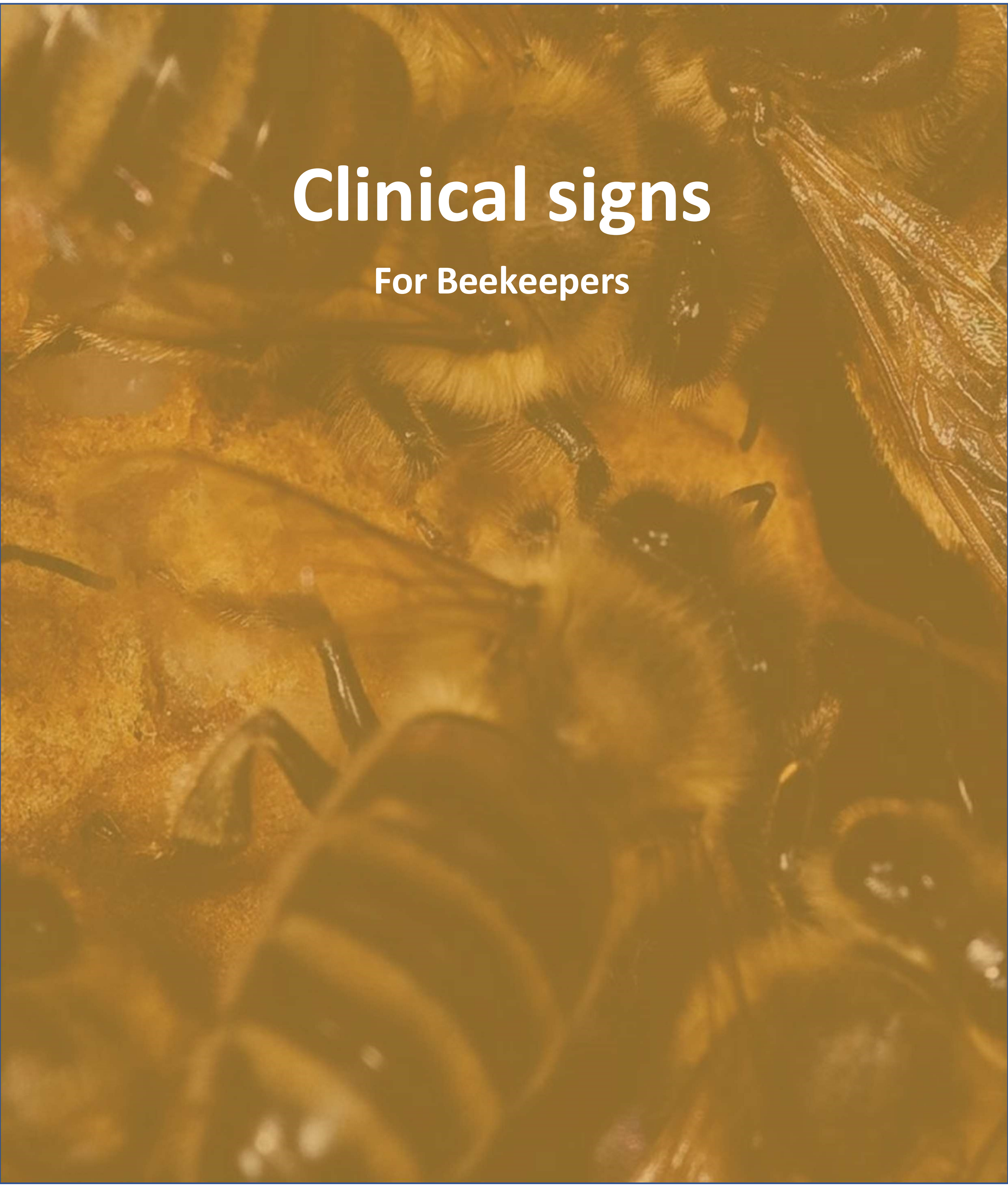

Clinical signs

For Beekeepers

This protocol was developed for B-GOOD project. B-GOOD stands for Giving Beekeeping Guidance by computational-assisted Decision making. The B-GOOD project received funding from the European Union's Horizon 2020 research and innovation programme under grant agreement number 817622.

B-GOOD protocol title and code: Clinical signs (P7.2)

Description: How to visually check colonies for clinical signs of disease

Version: 20220202

B-GOOD Tier: Tier 2

Target group: Beekeepers

Clinical signs

During the beekeeping season, check the hive at least once a month for clinical signs of disease.

Field methods

- Visually observe colonies to assess the presence of clinical signs in brood and adult bees. A decision-making tree for clinical signs of diseases observed in European honey bee colonies can be found below (page 2 for brood and page 3 for adult bees). If you have the suspicion that a disease is present in the hive, record the type of disease in the BEEP app.

References

EFSA AHAW Panel (EFSA Panel on Animal Health and Welfare) (2016). Scientific opinion on assessing the health status of managed honeybee colonies (HEALTHY-B): a toolbox to facilitate harmonised data collection. EFSA Journal 14: 4578, 241 pp.

Kunneke A. Diagnoseboom. Imkerpedia. Published April 2022. Website: https://www.imkerpedia.nl/wiki/index.php/Bijenziekten_en_plagen

Kunneke A. Diagnoseboom. Newsletter Bees@wur, published April 2022. Website: http://mymeasuremail.com/880/Actions/Newsletter.aspx?historymessageid=30982

Diagnostic tree of honeybee and brood disease

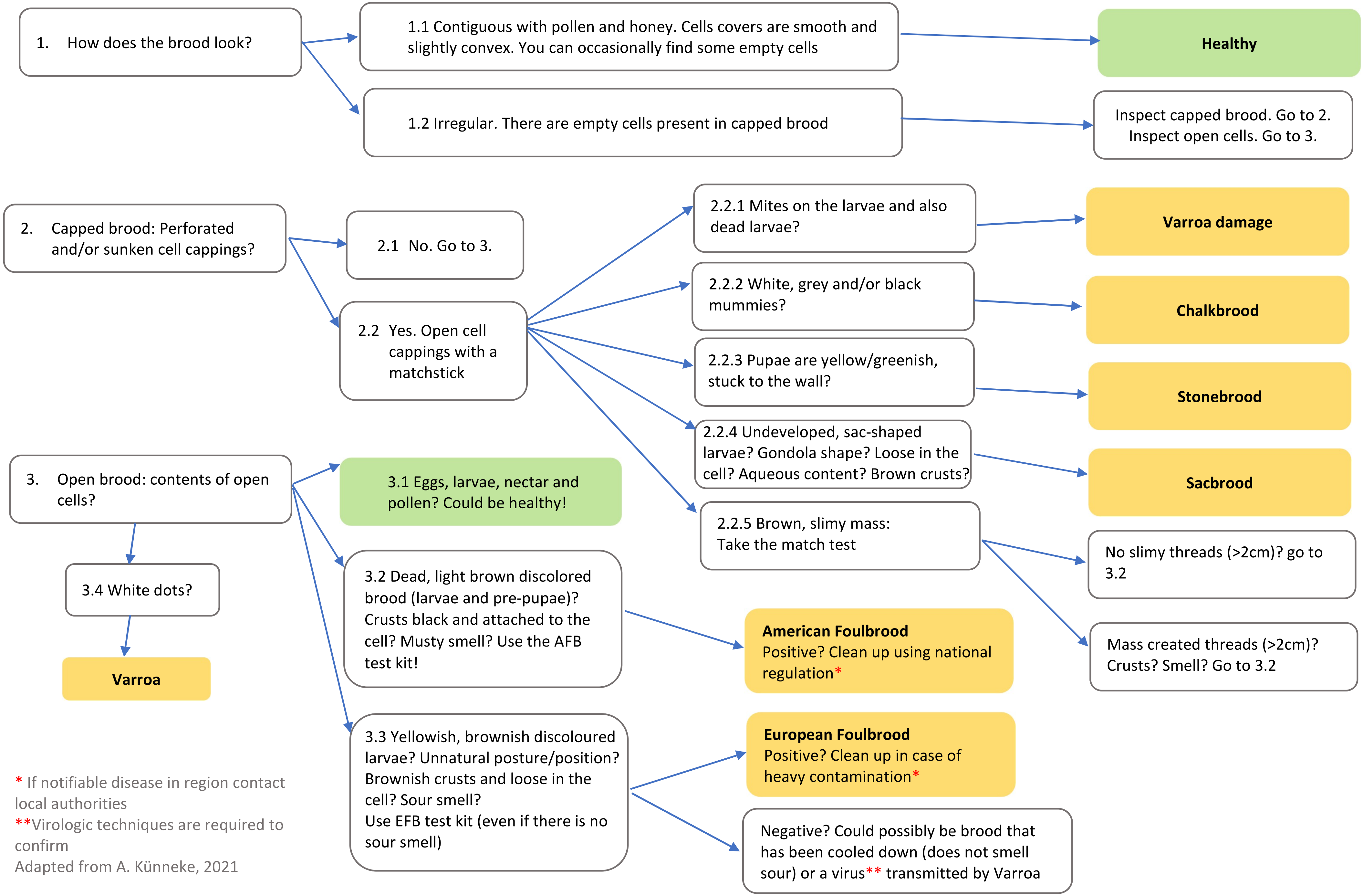

* If notifiable disease in region contact local authorities

**Virologic techniques are required to confirm

Adapted from A. Künneke, 2021

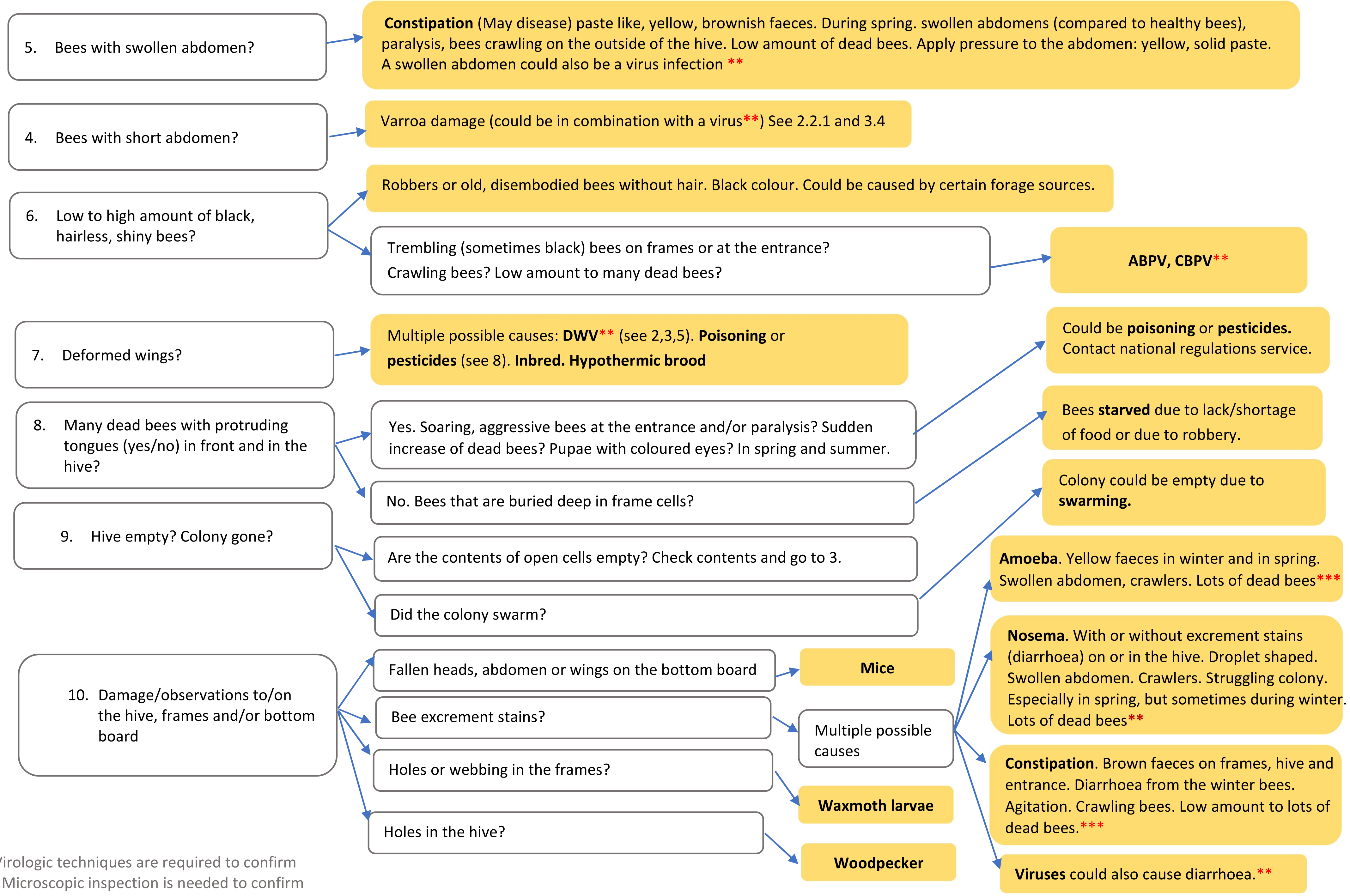

** Virologic techniques are required to confirm
*** Microscopic inspection is needed to confirm
Adapted from A. Künneke, 2021

Acknowledgments

The authors of this protocol thank the B-GOOD Tier 1 and Tier 2 partners for their constructive feedback during the project duration to optimize the content for high quality data collection in the project. The B-GOOD project received funding from the European Union’s Horizon 2020 research and innovation programme under grant agreement number 817622.

B-GOOD partners:

This protocol was developed for B-GOOD project. B-GOOD stands for Giving Beekeeping Guidance by computational-assisted Decision making. The B-GOOD project received funding from the European Union’s Horizon 2020 research and innovation programme under grant agreement number 817622.

Queen cell presence

For beekeepers

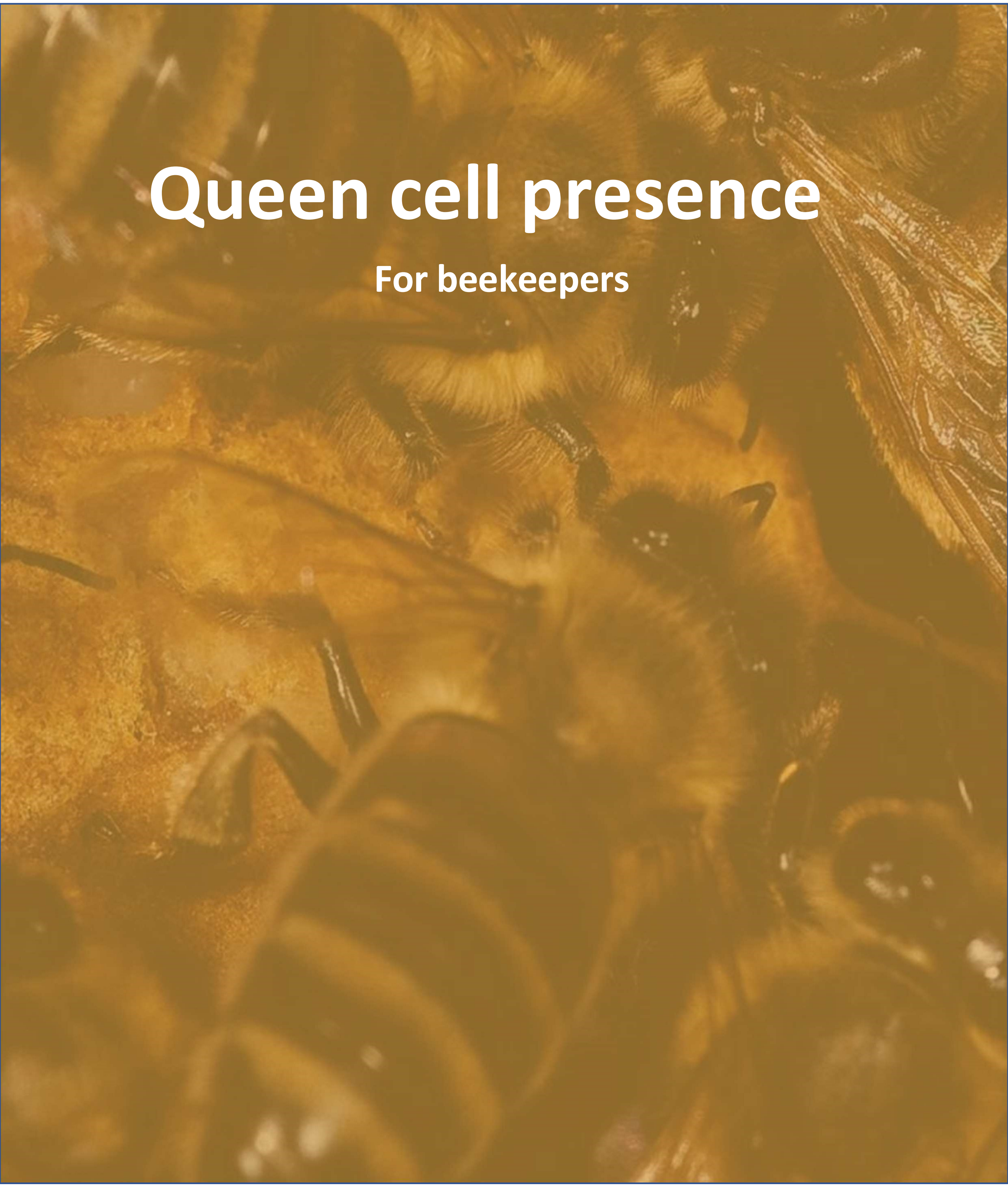

This protocol was developed for B-GOOD project. B-GOOD stands for Giving Beekeeping Guidance by computational-assisted Decision making. The B-GOOD project received funding from the European Union's Horizon 2020 research and innovation programme under grant agreement number 817622.

B-GOOD protocol title and code: Queen cell presence (P10.2)

Description: Checking the colony for queen cells and explaining the four different queen cell types

Version: 20220224

B-GOOD Tier: Tier 2

Target group: Beekeepers

Queen cell presence

During beekeeping season, especially after the swarming period has ended, check the colonies each time that they are opened or at least 1x a month for the presence of queen cells.

Field methods

- Open a colony and sequentially remove frames from the hive.
- Shake or brush every brood comb free of bees and examine carefully for presence of queen cells.
- Record the presence and type of queen cell in BEEP app (queen cup, emergency cell, swarm cell, supersedure cell).
- If queen cells are removed, please include this information as a management action.

Queen cell types:

- Queen cup: It is a small cup, with an opening on the bottom (Figure 1.A). For the purposes of this experiment, we define queen cups as empty queen cells (without eggs or larvae)
- Swarm cells: Are built when the colony is preparing to reproduce and swarm. These cells are usually present on the edges of the comb (Figure 1.B).
- Supersedure cells: Are built when the colony wants to replace the current queen. These cells are generally found on the centre of the comb (Figure 1.C.), but are not created from modified worker cells. Commonly the colony raises 1-3 supersedure cells.
- Emergency cell: Are built if the old queen is dead. Like supersedure cells, they are usually found on the centre of the comb (Figure 1.D), but these cells are created from modified worker cells. Usually, the colony raises a high number of emergency cells (especially compared to supersedure cells).

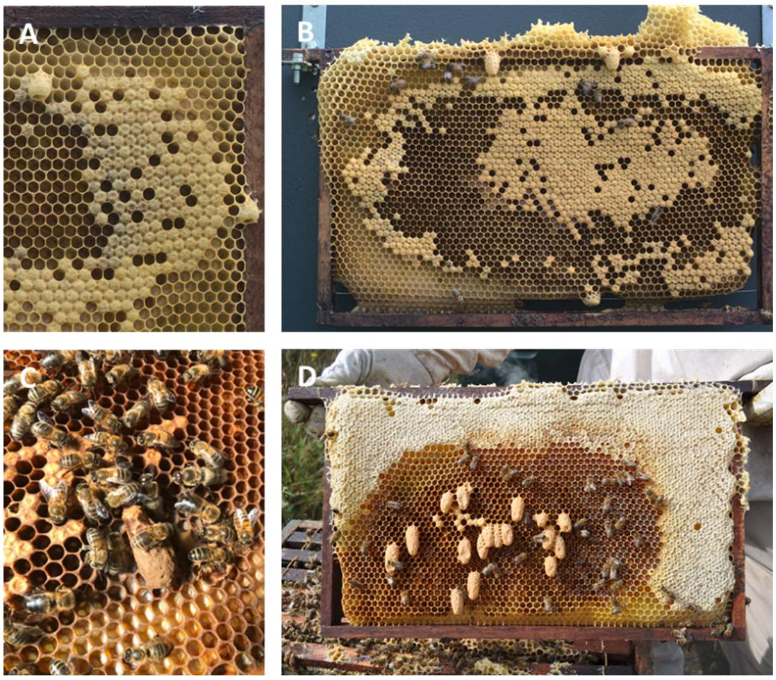

Figure 1: Different types of queen cells in honeybee colonies. A) Queen cups; B) Swarm cells; C) Supersedure cells if the queen is alive or emergency cells if the queen is dead; D) Emergency cells. Photo credits: C.van Dooremalen (A,B,D), M. Schoonman (C).

References

Delaplane KS, Van Der Steen J, Guzman-Novoa E (2013). Standard methods for estimating strength parameters of *Apis mellifera* colonies. Journal of Apicultural Research 52: 1-12.

EFSA AHAW Panel (EFSA Panel on Animal Health and Welfare) (2016). Scientific opinion on assessing the health status of managed honeybee colonies (HEALTHY-B): a toolbox to facilitate harmonised data collection. EFSA Journal 14: 4578, 241 pp.

Human H, Brodschneider R, Dietemann V*, et al*. (2013). Miscellaneous standard methods for *Apis mellifera* research. Journal of Apicultural Research 52: 1-53.

Acknowledgments

The authors of this protocol thank the B-GOOD Tier 1 and Tier 2 partners for their constructive feedback during the project duration to optimize the content for high quality data collection in the project. The B-GOOD project received funding from the European Union's Horizon 2020 research and innovation programme under grant agreement number 817622.

B-GOOD partners:

This protocol was developed for B-GOOD project. B-GOOD stands for Giving Beekeeping Guidance by computational-assisted Decision making. The B-GOOD project received funding from the European Union's Horizon 2020 research and innovation programme under grant agreement number 817622.

Drone Presence

For beekeepers

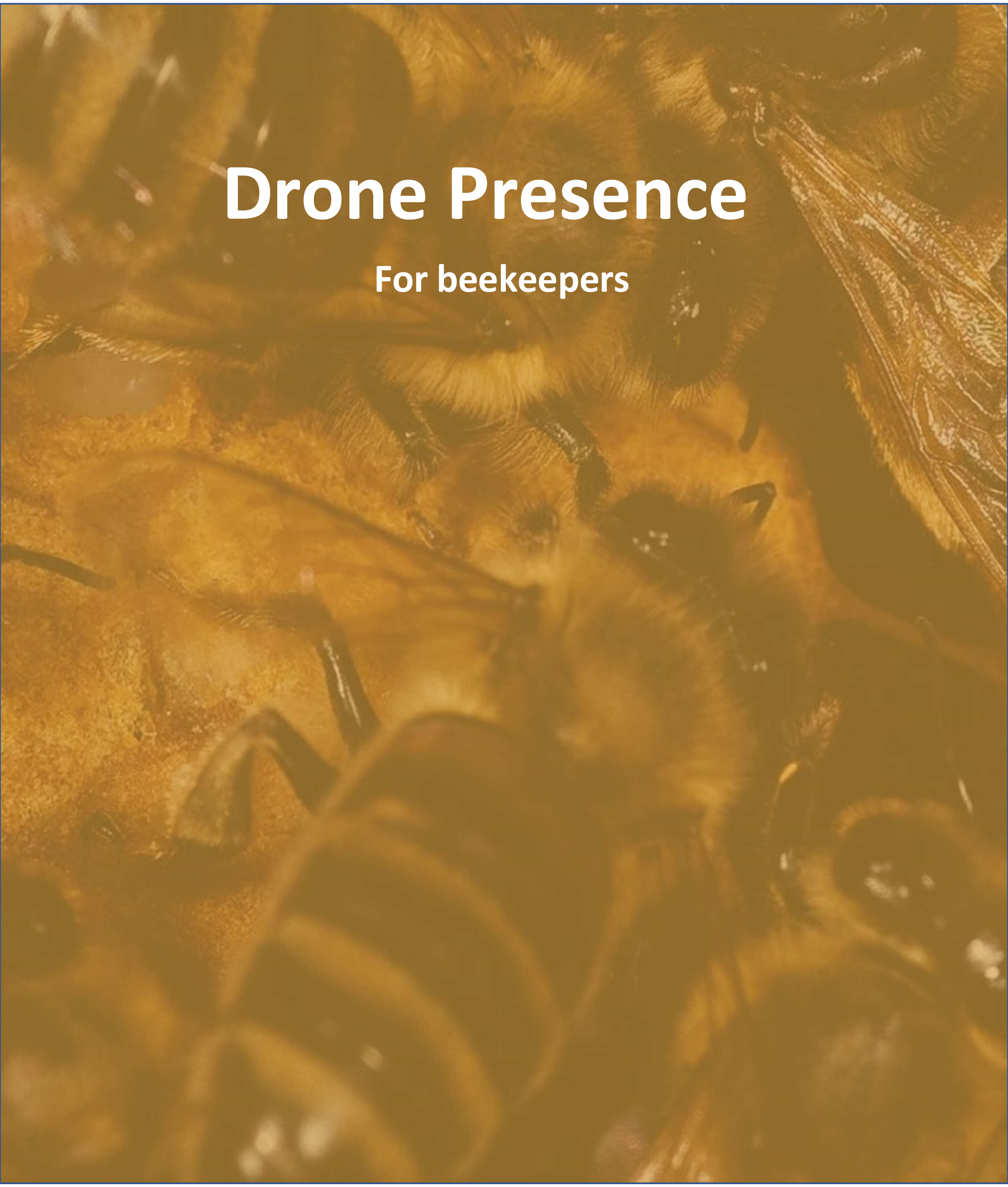

This protocol was developed for B-GOOD project. B-GOOD stands for Giving Beekeeping Guidance by computational-assisted Decision making. The B-GOOD project received funding from the European Union's Horizon 2020 research and innovation programme under grant agreement number 817622.

B-GOOD protocol title and code: Drone presence (P13.2)

Description: Checking colonies for presence of drone brood.

Version: 20220513

B-GOOD Tier: Tier 2

Target group: Beekeepers

Drone presence

During swarming season, beginning in spring from the start of bee foraging activity, check colonies for presence of drone brood. Continue checking until all of the drones have died and the reproductive phase is finished. The presence of drone brood gives an indication of swarming tendency in colonies and also of colony health. Healthy colonies are willing to reproduce, and able to invest resources in drones.

Colonies should be checked for drone brood at least 1x a month or each time the colony is opened.

Field methods

- Open a colony and sequentially remove frames from the hive.
- Check the hive comb surface to see for the presence of drone brood.
- If drone brood present, record it in the BEEP app.

References

Delaplane KS, Van Der Steen J, Guzman-Novoa E (2013). Standard methods for estimating strength parameters of *Apis mellifera* colonies. Journal of Apicultural Research 52: 1-12.

EFSA AHAW Panel (EFSA Panel on Animal Health and Welfare) (2016). Scientific opinion on assessing the health status of managed honeybee colonies (HEALTHY-B): a toolbox to facilitate harmonised data collection. EFSA Journal 14: 4578, 241 pp.

Acknowledgments

The authors of this protocol thank the B-GOOD Tier 1 and Tier 2 partners for their constructive feedback during the project duration to optimize the content for high quality data collection in the project. The B-GOOD project received funding from the European Union's Horizon 2020 research and innovation programme under grant agreement number 817622.

B-GOOD partners:

This protocol was developed for B-GOOD project. B-GOOD stands for Giving Beekeeping Guidance by computational-assisted Decision making. The B-GOOD project received funding from the European Union's Horizon 2020 research and innovation programme under grant agreement number 817622.
