# Supplementary material for: Bridging the Gap between Field Experiments and Machine Learning: The EC H2020 B-GOOD Project as a Case Study towards Automated Predictive Health Monitoring of Honey Bee Colonies
Source: Insects. 2024 Jan 22;15(1):76. doi: 10.3390/insects15010076 (PMC10816039; doi:10.3390/insects15010076)
Supplement: Supplementary file 1 [file insects-15-00076-s001.zip › S1_tier 3_workplans and protocols.pdf]

B-GOOD

WAGENINGEN
UNIVERSITY & RESEARCH

Workplan Tier 3

For beekeepers

Supplementary information S1 belonging to

Van Dooremalen C, Ulgezen ZN, Dall'Olio R, *et al.* Bridging the gap between field experiments and machine learning: The EC H2020 B-GOOD project as an example method to work towards automated predictive hive monitoring and healthy honeybee colonies.

This workplan was developed for B-GOOD project. B-GOOD stands for Giving Beekeeping Guidance by computational-assisted Decision making. The B-GOOD project received funding from the European Union's Horizon 2020 research and innovation programme under grant agreement number 817622.

B-GOOD workplan title: Workplan for B-GOOD selected beekeepers (TIER3, WP1)

Version: 20220524

B-GOOD Tier: Tier 3

Target group: Beekeepers

Comment: This is an exact copy of the workplan as it was used in the B-GOOD project

Workplans may be updated during the project period. Always ensure you have the latest version of the workplan.

Experiment Set-up

B-GOOD is an European Union project. The B-GOOD project has the overall goal to provide guidance for beekeepers and help them make better and more informed decisions. For the recruitment of beekeepers willing to participate in the project a selection survey was made and spread throughout Europe. Based on the outcome of this survey 58 beekeepers across Europe were selected to participate in this study.

Each beekeeper will keep three (presumably) healthy colonies at their apiary/apiaries generating data using the BEEP base. 58 beekeepers are included in the project each with 3 colonies, 174 colonies in total. The experiment will run for one year (2022-2023).

You were selected; hence we share this workplan with you to participate in this Study of the B-GOOD project.

The aim of the study is to:

1) Validate new technologies for automated measurements of health status.
2) Develop a system that provides beekeepers with automated beekeeping guidance.

This workplan facilitates the methods and protocols for all preparations and measurements during the experiment. As such, in three of your colonies we ask you to do some classical methods of measuring bee health by visual inspection. For automated measurements you will be supplied with a BEEP system (3 BEEP bases and 1 BEEP app account per beekeeper) to be used the allocated colonies. BEEP is a system built to support beekeepers, where observations of the hive can be digitally registered using a record keeping app (BEEP app).

BEEP System

BEEP app

We will use the BEEP app to collect and store data throughout the research period 2022 in a standardized way for:

1. Registration and consent to use your data
2. Information on the apiary level
3. All management actions related to beekeeping
4. All experimental observations

Please go through the following steps related to the BEEP app, once or throughout the research period (depending on the step)

1. **Registration and consent to use your data** (once)

See Appendix 1 how to go to and register in the BEEP app and consent to participate in the research. We make use of the 'web app' of the BEEP platform to log all information on the apiaries. Web app means that you can open the app on your mobile phone, tablet and computer and that you do not have to download anything.

2. **Entry of information on the apiary level** (once, but keep up to date during the research period)

In the BEEP app you need to store your meta data entry on the apiary level, e.g. location, colony numbers, type of hives, number of brood boxes. Select 3 of your colonies and enter their data into the app. If they are all in one location, you can create one apiary with the three hives*. If you allocate hives in different apiaries, make multiple apiaries in the app.

*you may use the app for all your hives if you wish. It would be very welcomed by us. Do realise that such additional hive (and inspection) information will also be shared automatically for research purposes if the hives are on the same account that you use for the B-GOOD project.

3. **Entering all management actions related to beekeeping** (throughout the research period)

Please record all management actions related to beekeeping that are done throughout the research period in the BEEP app. For example, this involves adding or removing of brood boxes or honey supers, splitting colonies, queen replacement, feeding, varroa treatment and all other actions. To enter management actions, the user is asked to create a customized inspection sheet. This custom inspection sheet can be adapted according the beekeeper specific actions and time of season. See the following BEEP link for a manual for creating such customized inspections sheets. All data must be recorded electronically. Use the appropriate fields to enter the data and only use the notes field if there are no other options, in order to ease data analysis. Please write the notes in English. When you click the save button in the BEEP app, the data will be stored on the BEEP server. You can always access your own inspection data (see this manual how to access) and sensor data (in app click Menu>Measurements) in the app or by downloading it (see this manual how to download your data). We prefer you to manage your bees in your own way. How to deal with changes regarding colonies throughout the study period from a data collection perspective, is explained in the section *'Beekeeping Management'* in this document.

4. **Entering all experimental observations** (throughout the research period)

All experimental observations need to be entered in the BEEP app. For the experimental observations standard inspection sheets have been prepared for you. The inspection sheets can be found in the BEEP app (Figure 1, where to find them in the app). In the section '*Experimental Observations'* of this workplan, you can find information on when and which protocols and inspection sheets should be used throughout

the measurement period in 2022/2023. The inspection sheets can only be seen and used, after consenting to participate in the research (Step 1).

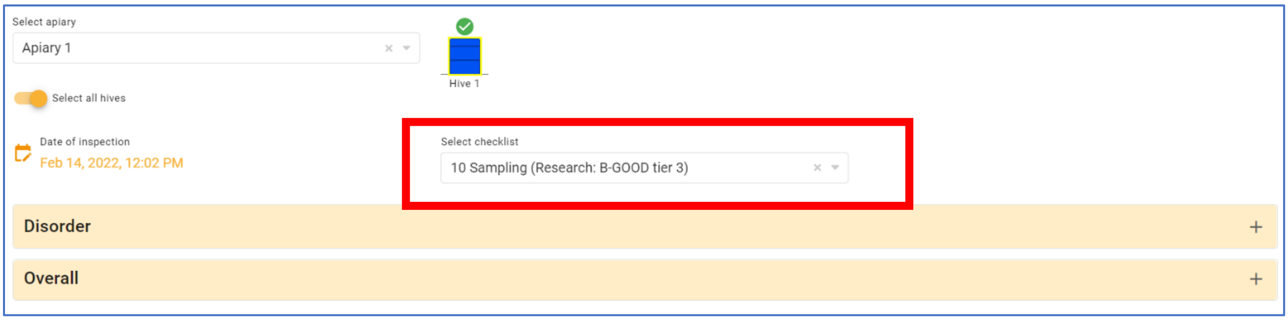

Figure 1: The section for selection of checklist for data entry. Select the appropriate checklist for data collection depending on type of experimental observation and timing of data collection (see Table 1). The bottom checklist, with your email address included is the list that can be customized for management actions.

5. Some general remarks about the BEEP app

When you click the save button in the BEEP app, the data will be stored on the BEEP server. Each participant will have continuous access to their own collected and stored data. WR will have access to all data collected in this study and will process the data (including pseudonymisation) for further use within the B-GOOD project together with the BEEP team.

At each hive inspection, the BEEP app on your phone or computer must be connected to the internet to send inspection data to the server. The BEEP app automatically registers the date and time when you enter data into the system. Information to the app can also be added at a later date. However, it is important to change and correct for the date of inspection, and adjust it to the original moment of data collection if data is entered at a later date (Figure 2).

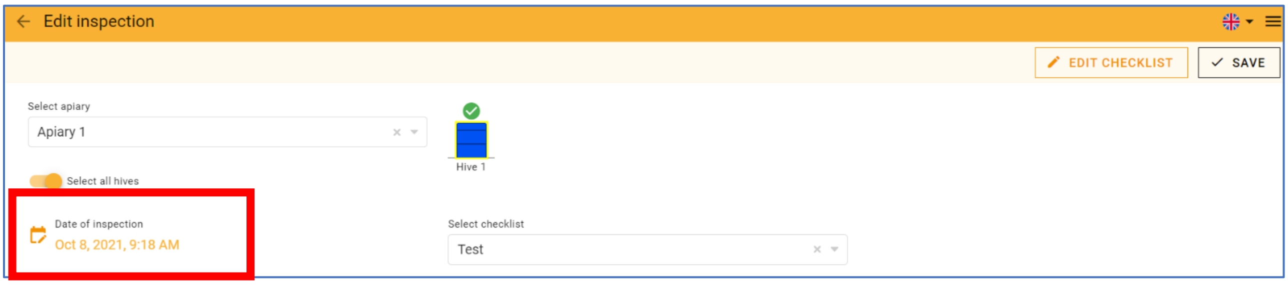

Figure 2: The section for entry of date of inspection (observations and actions) in the BEEP app. If data is entered at a later date adjust it to the original moment of data collection. Date can be changed by just clicking on the date in the red box.

Please note that it is possible to enter data into the BEEP app in bulk. This recently included function was developed within the B-GOOD project, based on feedback by B-GOOD users. Before this function was included you could only enter data per colony. For instance, if you did oxalic acid treatment for all colonies within a day, you had to enter the information for each colony individually. Now, you can enter it once and select all colonies. So, it will be recorded for all colonies.

BEEP base and sensor placement

At the start of the experiment, you will get 3 BEEP bases (Figure 3) to install under your selected hives. During the experiment we will continuously monitor hives with the BEEP base, which measures weight, temperature and sound in the hive. BEEP will provide an installation manual that includes information on the set-up of the BEEP base (for an online version of the installation manual click here). The BEEP bases are in the box that you receive in the beginning of the experiment, which also includes screws (for BEEP base) and sample cups (further information in the protocol Sampling for lab analyses) . The additional screws are stainless steel. Please use these screws for the BEEP base battery compartment, instead of the original ones provided in the BEEP base box (Figure 5).

See below for set-up of BEEP base and sensor placement in the hive.

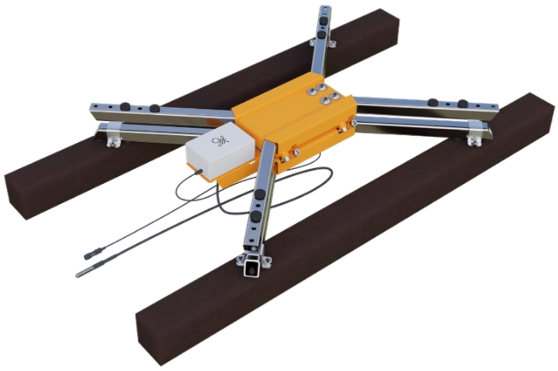

Figure 3: The BEEP base that will be placed under each of the 3 hives in your apiary after you installed the colonies. **Note that the computer (white box) is placed to the side of the hive. The BEEP base dimensions are such that most hives fit well and stable when the BEEP base is placed this way. Turning it 45 degrees is not preferred.**

Scale. A weight sensor is at the centre of the steel construction of the BEEP base. The BEEP base needs to be placed underneath the hive for continuous weight measurements (Figure 4).

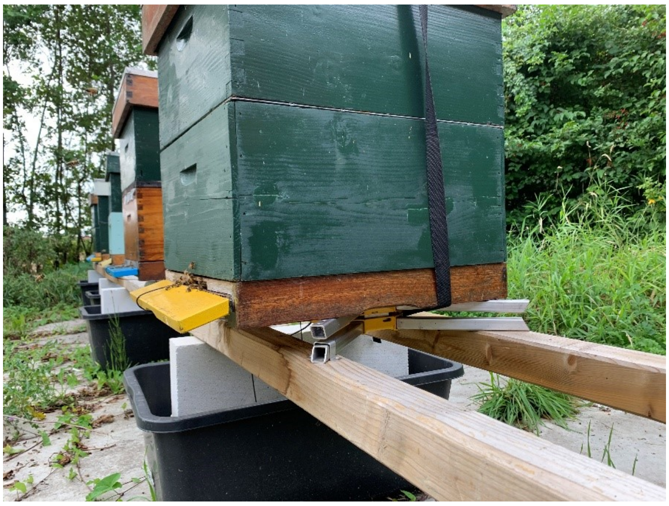

Figure 4: Placement of weight sensor underneath the hive.

Thermo-sensor. A thermo-sensor is connected through a cable to the BEEP base, and will be used for continuous temperature measurements. You received a wooden template as a holder for the temperature sensor (Figure 5). Please use this to fix the temperature sensor in the hive. Just put the cable through and place it on top of the frames to stop the cable and sensor from movement. The sensor needs to be placed in the brood box, on top, between the mid-frames (Figure 6). If the hive has several brood boxes, and there is a queen excluder, keep the temperature sensor in the box with the queen. If there is no queen excluder, always keep the sensor in the top brood box.

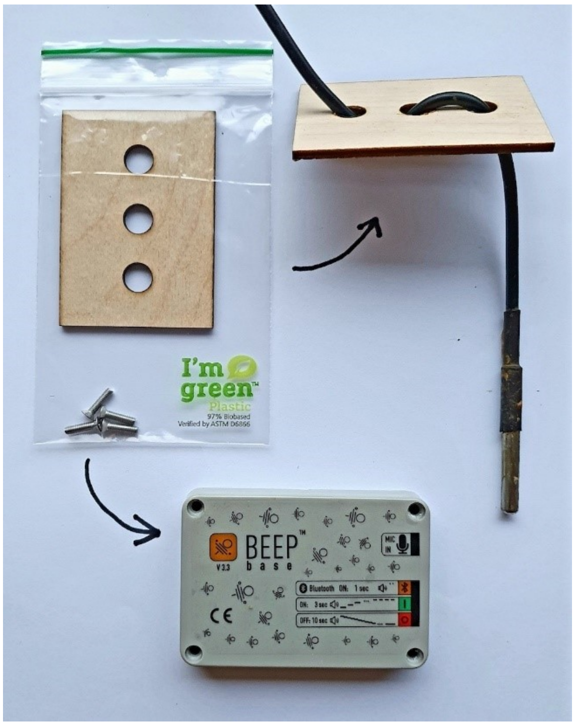

Figure 5: Placement of stainless-steel screws and wooden template for the temperature sensor.

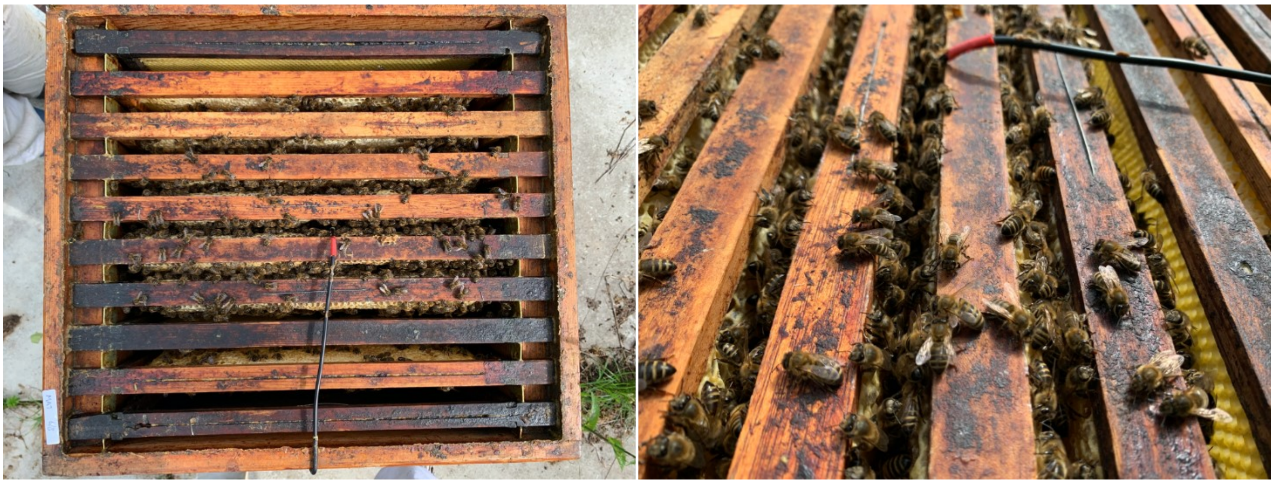

Figure 6: Placement of temperature sensor in the hive. It is approximately 9 cm from the red tape to the tip of the temperature sensor.

Microphone. A microphone is connected through a cable to the BEEP base, and will be used for constant sound measurements. The sensor needs to be placed at the center of the bottom frame, facing the back

of the hive, opposite to the hive entrance (Figure 7). Please be careful with the sensor and cable, in case you vaporize oxalic acid, using a heating plate at the bottom of the hive. The cable and sensor cannot handle the heat well.

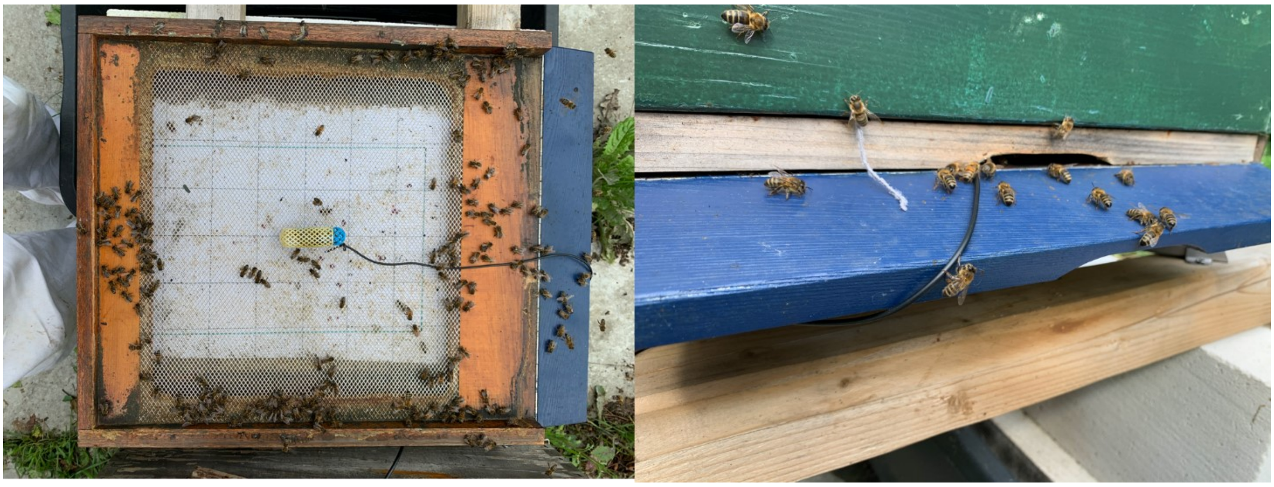

Figure 7: Placement of microphone in the hive. The cable can be placed through the flight entrance.

Beekeeping management

In the beginning of the experiment, three colonies need to be allocated to B-GOOD TIER3 and need to be kept with the tools (BEEP base) at all times. The criteria for keeping colonies are as follows:

- Colonies can be replaced by a new colony in case they die. This must be clearly indicated in the BEEP app.
- The colonies can be moved to a new location but not sold during the study period.
- The colonies should be kept at a location with cellular network and data connection (2G, 3G or 4G) for data collection purposes with BEEP base.

The basic principle of management actions within this study will be to: maintain healthy colonies; while at the same time respecting the nature of the bees; and providing care accordingly.

We will not provide you with standard beekeeping protocols or guidelines. The colonies should be managed accordingly with your regular beekeeping practices. However, all decisions made should align with the following criteria:

1) Colonies should be kept healthy (please, give it your best effort)
2) Enter all actions and changes in the BEEP app (e.g. swarm prevention, splitting, providing space, queen replacement, merging, reduce brood boxes, removal of brood, addition of bees and provision of nutritional supplements, varroa treatments, etc) in inspections for the appropriate hive. Please ensure the correct date and time is entered for actions. See section *'BEEP app'*, step 2 and 3.
3) If a colony dies, take a sample of bees (and perhaps brood also, if brood diseases are expected) and store until the regular sampling will take place (see experimental measurements). See also

the section colony mortality below (in Data collection), on how to deal with a lost colony in the web app.

4) If honey is harvested, then record how much honey is harvested per hive and the date of harvest (in BEEP app Inspection under Production > Honey). You may exclude the weight of wax seals from the calculations (automatically adding that weight to the honey harvest) in case you calculate the kg honey by: kg of the full honey super at harvest - kg of the empty super and empty frames after honey extraction.

Data collection: experimental observations

Table 1 (see page 14) gives the overview of required experimental observation we ask you to conduct over time, and the related inspection sheets that we have prepared to log those observations. Please stick to the planning as much as possible. Detailed information about methods for data collection are provided in protocols that will be incorporated in the app. Additional information about the data collection methods that is needed throughout the experiment is detailed below (with the related protocol number between brackets). Try combining activities to minimize colony disturbance.

In this study (TIER3 of the B-GOOD project), we ask you to conduct the following:

- Checking for the presence of queen and brood,
- Sampling bees for lab analyses.
- Providing subjective measures on colony health in two ways ('Overall Impression' and 'Colony Health')
- Recording colony mortality

These measurements are explained in further detail below. At the bottom of the document, we give an overview on the timing of these measurements (Table 1).

Presence of queen and brood

Every time you open the colony for regular beekeeping actions, starting from the end of the winter period till the end of the beekeeping season (essentially, the period of honey bee foraging activity), check the hive comb surface for the presence of queen and of all stages of brood. The presence of worker brood gives information on queen fecundity, viability of worker force and the ability of the colony to rear the eggs until adulthood.

Field methods

- Open a colony and sequentially remove frames from the hive.
- Check the hive comb surface until the presence of queen and of all life stages of brood –eggs, larvae, pupae – are verified. The queen should be labelled for easy detection.
- If queen is not found and/or no open brood is present, queen failure is assumed (after rechecking in 1 week). Queen presence can be presumed, if open brood is still present.
- Please be aware that the queen might stop laying eggs prior to swarming, in early winter and during extreme weather events.

- Record queen presence, brood presence and any replacement queens in the BEEP app in Inspection sheet “9 Health” as indicated in Table 1.

Sampling bees for lab analyses

Lab analysis on diseases and genotyping will be done through Reference Labs. For this, samples of bees will be collected three times a year. First in spring, when the bees start to forage; second time in summer, when the colonies have reached their maximum size; and a third time in autumn, before the over-wintering. As spring, summer and autumn will be shifted between participating beekeepers, the sampling moments need to be adjusted by participants depending on the climate of country. The first (spring) visit should take place as soon as major pollen producing plants are flowering. Look out e.g. for willow plants *(goat willow or gray willow*). The spring visit should take place within three weeks from the moment these plants start flowering. The second (summer) visit should take place about 2 weeks after midsummer, or if known, the moment of peak colony size that naturally occurs in region. The third (autumn) visit should take place before it gets too cold to sample (< 10°C). Depending on the location of the apiary, the visiting time can be from around the end of September until the end of November.

We ask you to sample (alive) bees for diseases, as explained, below and send the samples to either a local partner in your country, or directly to the reference laboratory responsible for processing your samples. As details about shipment may differ between countries, we will inform you about this on a personal basis. From the reference laboratories, the samples are further processed and shipped for the additional B-GOOD analyses that are performed. We need to process a lot of samples, you will get the results, but it may take some time, before you get them (especially for the genotyping).

BEEP app

- For anonymization purposes, the BEEP app will generate a unique identifier per sample. The samples should be labelled with these IDs. Use Inspection sheet “10 sampling” as indicated in Table 1. Under the tab “Disorder” the unique sample code can be generated.
- Please label the samples on the container and additionally place a label inside the container (on paper with a pencil), to freezer proof label and ensure identification of the sample, even if the outside label gets damaged or disappears during transport.
- Make sure the samples collected are correctly linked to the IDs to ensure correct feedback from the lab.
- Record the date of sample collection in the BEEP app.
- Check that the date and time of the inspection containing the sample code in the BEEP app corresponds with the actual sample collection date and time.

Materials

- 3 x freezer containers with drilled holes (Figure 8)
- 3 x halved queen cages with sugar dough in it
- Labels

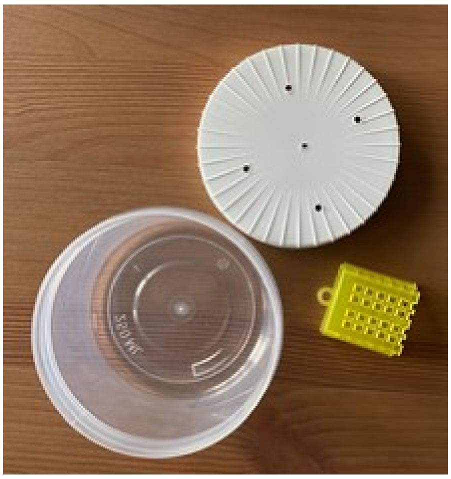

Figure 8: Example of freezer container with holes.

Collection of bees from the colonies

- Before sampling, place the halved queen cage with sugar dough into the container to equip bees with some food prior to transport.
- To collect samples, first open the colony and check the combs starting with the frames on the outer edge.
- Remove the first frame fully occupied by bees (preferably one at the periphery of the brood nest).
- Make sure that the queen is not present on the comb, if present return her to the hive (or take another frame).
- Fill the freezer container with about 300 bees (as an indication, a sample container with 300 bees (but without queen cage) will way a minimum of 64 grams, if you also included a queen cage with dough, the container with bee and dough cage should weigh a minimum of 90 gram).
- One of the best methods to do this is to shake the bees from the frame onto a sheet, then bend or fold the sheet and use it to fill the bees into the cup. Many colonies have a covering foil on top underneath the lid. One can use this sheet but you can also bring a sheet or use a sheet of newspaper. The cup should be placed on the top of the open colony so all bees that spill out of the cup fall directly back into the colony.
- Alternatively, if the bees are calm, fill the cup by scraping the bees off the comb, holding the cup vertically and the comb at 45°.
- It is fine if drones are included in the sample but less is better.

Transport

- Place the freezer containers with live bees into a shipping box to prepare for transport. Make sure the transport box has ventilation holes as well. Please make sure that the containers are firmly closed and cannot open during transport.
- The samples should be sent the same day of sampling in the field because the bees should arrive alive.
- Transport details will be communicated to you personally as these may differ between countries.

- Coordinate the dates of transfer for samples with the B-GOOD partner organisation in your country before arranging transport. This in order to make sure the receiving person is available for the package and further processing.

Overall impression

Information on the overall impression of the health of each colony will be collected throughout the whole year. There are three different categories in the BEEP app for the health status (Figure 9). A description or definition of categories will not be provided, as this is a subjective measurement by the observer. Entry of data should be done at least once a month, but can also be done more frequently, up to once a week. This measurement is included at the end of each inspection sheet (see Appendix 1). If you use multiple inspection sheets during one inspection, filling in the overall impression on only one of the inspection sheets will suffice.

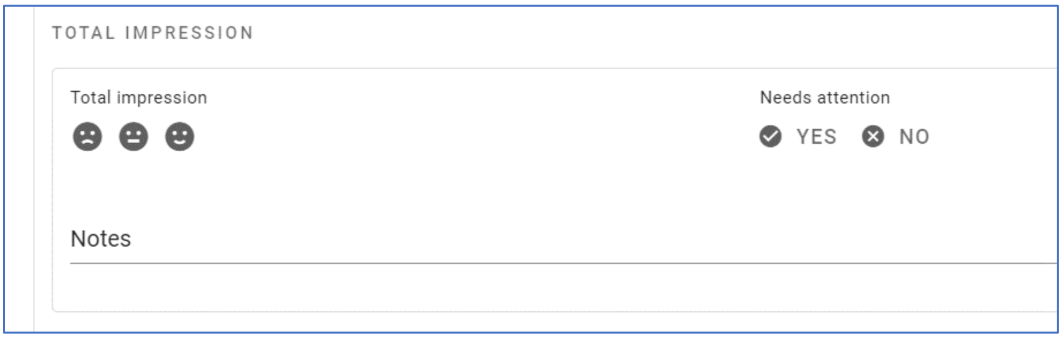

Figure 9: Data entry for overall impression on the health of colonies in the BEEP app.

Colony Health

Information on the health status of the colony will also be collected with a checklist on the categories: sufficient adult bees (yes/no), suitable space (yes/no), absence of stressors (yes/no), sufficient nutrition (yes/no) (Figure 10). Similar to the *Overall impression*, a description or definition of categories will not be provided as this is a subjective measurement by the observer. Entry of data should be done at least once a month, but preferably should be done as often as possible, every time the hive was opened.

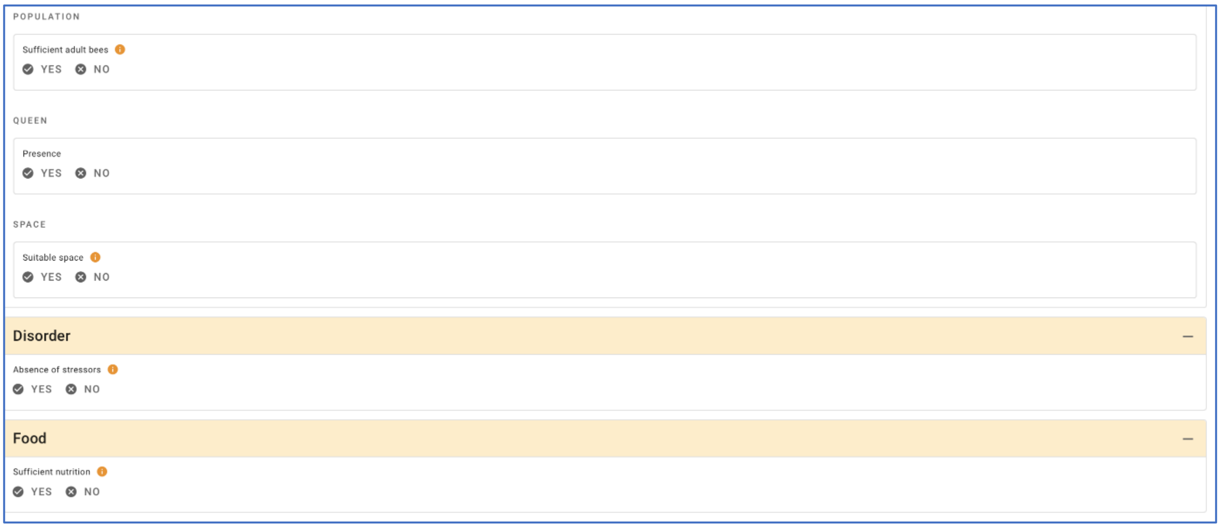

Figure 10: Part of the data entry for data annotation in the BEEP app (inspection sheet Health).

Colony mortality

Throughout the year, information on colony mortality and observations on dead colonies should be recorded. A colony is considered dead if 1) the hive is absent of any living bees 2) the colony is too weak to recover in spring because (i) less than two frames are occupied by winter bees or (ii) the queen is dead and the colony cannot replace queen by building emergency cells (no brood).

In case a colony dies, replace it with a new one and record actions in BEEP app. If possible, take a sample of the remaining dead bees and store until the upcoming sampling round for disease and include this sample in the batch, with a special marking “Dead” and the (approximate) date it died.

If a colony dies, please record it in the BEEP app by following these steps:

1. Create new hive for the new colony on the BEEP base and add 'b' to name. Do this also, if you repair the ‘old’ colony with a newly imported mated queen or e.g. when you introduce a frame of eggs of another colony to help emergency queen cell formation.
2. Add 'a' to dead colony
3. Change colour (white) of dead colony to help ensure it stands out and no new inspections are added
4. Add inspection to indicate that it is dead (under Colony loss in BEEP app), why, when and add red smiley
5. Add inspection to new colony that it is new and when
6. Move base to new hive (previous sensor data is connected to the base). The dates in the inspections help in data processing to figure out which colony the sensor data belongs to and when it was switched between the dead and the new colony.

Acknowledgments

The authors of this workplan thank the B-GOOD Tier 1 and Tier 2 partners for their constructive feedback during the project duration to optimize the content for high quality data collection in the project. The B-GOOD project received funding from the European Union's Horizon 2020 research and innovation programme under grant agreement number 817622.

B-GOOD partners:

This workplan was developed for B-GOOD project. B-GOOD stands for Giving Beekeeping Guidance by computational-assisted Decision making. The B-GOOD project received funding from the European Union's Horizon 2020 research and innovation programme under grant agreement number 817622.

Table 1. Overview of experimental observations over time. The protocol number, the timing (frequency) of measurements and the months in which they are to be performed are provided for each activity. Coloured cells show in which months experimental observations are expected to be performed. Two inspection sheets were prepared for use in the BEEP app, covering the protocols: 9 Health; 10 Sampling.

		**Months**											
**Experimental observation**	**Timing**	*Jan*	*Feb*	*Mar*	*Apr*	*May*	*Jun*	*Jul*	*Aug*	*Sep*	*Oct*	*Nov*	*Dec*
Presence of queen & brood	1 x month *												
Sampling for lab analyses	3 x year *												
Overall impression	1 x week **												
Colony Health	1 x week **												
Colony mortality	1 x month *												
**BEEP app inspection sheets to be used**		**9**	**9**	**9**	**10**	**9**	**9**	**10**	**9**	**9**	**10**	**9**	**9**

** The months may vary between countries; dates are only provided as an outline, and to represent activities that can be combined. Participants should make 'local' decisions on timing of data collection, depending on colony status, phenological state and climate of country. See workplan section on experimental observations for more details. Preferably every time the hive is being opened.*
*** Preferably every time the hive is being opened, if the hives are not often opened then at least once a month.*

Appendix 1 – BEEP app registration

To start using BEEP for the B-Good project, please follow these steps:

Register in BEEP

- In the manual you can find out how you can place the BEEP app on your phone or tablet. You only need one account and with that account you can access BEEP on multiple devices.
- REGISTER: Go to the BEEP app, using a web browser on a computer or alternatively on a mobile phone via this link for the English version. As a new user, click on the login screen on 'No account yet? Register as a new user'. Register with your work email address and follow the instructions. See the login support article for more information on this step.
- APIARY: When logging in for the first time, you will see the 'Create new apiary' screen where you can add the B-GOOD apiary details. When you are done, click on 'Create new apiary' button to save the data. See the Create a new apiary article for more information on this step.
- HIVES: Open the apiary you created. You can change the settings per hive, by clicking on the hives. You can change the configuration and enter the details on the queens per hive. See this article for more information on this step.
- INSPECTIONS: By clicking on the pen icon under each hive, you can add inspections for that hive. This is also further described here.
- RESEARCH: An important step is to link your account to the B-GOOD research program. You only need to do this once. You can click on 'Research' in the menu on the left and select the B-GOOD program by following the on-screen instructions (Figure 11). This way the data can be accessed for analysis in WP1. Please consent to share your data with B-GOOD Tier 3, and adjust the consent date to 01/01/2022.
- COLLABORATION: your co-workers can edit the data for your apiaries also if needed. You can see here how you can set this up for your group.

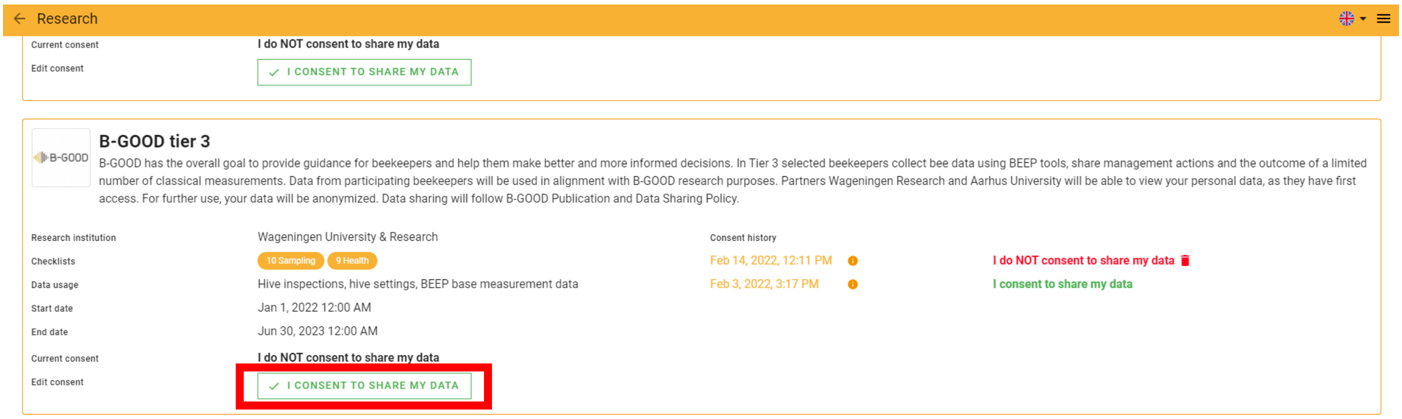

Figure 11: After creating your account on the BEEP platform, you can link your account to the B-GOOD programme by clicking on 'Research' in the left menu.
